# Assembling genomes of non‐model plants: A case study with evolutionary insights from *Ranunculus* (Ranunculaceae)

**DOI:** 10.1111/tpj.70390

**Published:** 2025-09-19

**Authors:** Kevin Karbstein, Nancy Choudhary, Ting Xie, Salvatore Tomasello, Natascha D. Wagner, Birthe H. Barke, Claudia Paetzold, John P. Bradican, Michaela Preick, Axel Himmelbach, Nils Stein, Argyris Papantonis, Iker Irisarri, Jan de Vries, Boas Pucker, Elvira Hörandl

**Affiliations:** ^1^ Albrecht‐von‐Haller Institute for Plant Sciences, Department of Systematics, Biodiversity and Evolution of Plants (with Herbarium) University of Göttingen Göttingen Germany; ^2^ Department of Biogeochemical Integration Max Planck Institute for Biogeochemistry Jena Germany; ^3^ Data‐Intensive Systems and Visualization Group (dAI.SY) Technical University Ilmenau Ilmenau Germany; ^4^ Institute for Cellular & Molecular Botany (IZMB) University of Bonn Bonn Germany; ^5^ Institute of Pathology University Medical Center Göttingen Göttingen Germany; ^6^ Senckenberg Naturhistorische Sammlungen Dresden Germany; ^7^ Institute for Biochemistry and Biology University of Potsdam Potsdam Germany; ^8^ Leibniz Institute of Plant Genetics and Crop Plant Research (IPK) Seeland Germany; ^9^ Department of Crop Sciences Center of integrated Breeding Research (CiBreed) Göttingen Germany; ^10^ Department of Biodiversity and Evolutionary Biology Museo Nacional de Ciencias Naturales (MNCN‐CSIC) Madrid Spain; ^11^ Institute for Microbiology and Genetics, Department of Applied Bioinformatics University of Göttingen Göttingen Germany; ^12^ Campus Institute Data Science (CIDAS) University of Göttingen Göttingen Germany; ^13^ Göttingen Center for Molecular Biosciences (GZMB), Department of Applied Bioinformatics University of Göttingen Göttingen Germany

**Keywords:** *de novo* assembly strategies, gene evolution, Illumina vs. Nanopore vs. PacBio sequencing, large non‐model plant genomes, mitogenome, nuclear genome, plastome, Ranunculaceae, *Ranunculus auricomus* species complex

## Abstract

Whereas genome sequencing and assembly technologies are improving, cost can still be prohibitive for plant species with large, complex genomes. As a consequence, genomics work on some taxa in evolutionarily pivotal positions in the vascular plant tree of life has been hampered. The species‐rich genus *Ranunculus* (Ranunculaceae) is an important angiosperm group for the study of polyploidy, apomixis, and reticulate evolution. However, neither mitochondrial nor high‐quality nuclear genome sequences are available. This limits phylogenomic, functional, and taxonomic analyses thus far. Here, we tested Illumina short‐read, Oxford Nanopore Technology (ONT) and PacBio (HiFi) long‐read, and hybrid‐read assembly strategies. We sequenced the diploid progenitor species *R. cassubicifolius* (*R. auricomus* species complex) and selected the best assemblies in terms of completeness, contiguity, and quality scores. We first assembled the plastome (156 kbp, 85 genes) and mitogenome (1.18 Mbp, 40 genes) sequences using Illumina and Illumina‐PacBio‐hybrid strategies, respectively. We also present an updated plastome and the first mitogenome phylogeny of Ranunculaceae, including studies of gene loss (e.g., *infA*, *ycf15*, or *rps*) with evolutionary implications. For the nuclear genome sequence, we favored a PacBio‐based assembly polished three times with filtered short reads and subsequently scaffolded into eight pseudochromosomes by chromatin conformation data (Hi‐C). We obtained a haploid genome sequence of 2.69 Gbp, with 94.1% complete BUSCO genes found and 35 482 annotated genes, and inferred ancient gene duplications compared to existing Ranunculales genomes. The genomic information presented here will enable advanced evolutionary‐functional analyses for the species complex, but also for the genus and beyond Ranunculaceae.

## Significance Statement

The genus *Ranunculus* is an important group of flowering plants for polyploidy, apomixis, and evolutionary research. We present the first nuclear and mitochondrial genome sequence, and the plastome evolution of Ranunculaceae. Using short‐ and long‐reads and state‐of‐the‐art bioinformatic tools, we found assembly and evaluation strategies that can also be applied to other non‐model plants. The results presented here are useful for improving phylogenomic, but also functional gene analyses at the species, genus, and family level.

## INTRODUCTION

Over the past decade, the scientific community has experienced a massive expansion in accessible sequencing technologies. For constructing genome assemblies, short‐reads (e.g., Prjibelski et al., 2020; Weisenfeld et al., 2018), long‐reads (e.g., Cheng et al., 2021; Koren et al., 2017), or a combination of both (hybrid‐read assemblers; e.g., Di Genova et al., 2021; Gatter et al., 2021; Zimin & Salzberg, 2022) have frequently been used. Nevertheless, Illumina short‐read sequencing (100–250 bp) often results in incomplete assemblies. Short‐read assemblies lack the contiguity needed to resolve repetitive regions in large genomes, leading to highly fragmented and incomplete de novo genome assemblies (Gatter et al., 2021; Rhie et al., 2021; Zimin & Salzberg, 2022). Today, long‐read technologies, such as Oxford Nanopore Technologies (ONT) or PacBio HiFi single‐molecule real‐time sequencing (SMRT; Pacific Biosciences), can bridge such gaps and improve the quality of genome assemblies. High‐fidelity (HiFi) reads derived from SMRT sequencing typically exhibit high base‐call accuracy (>99.5%) and up to 25 kbp in length. In contrast, ONT sequencing just crossed the 99% base‐call accuracy threshold, reaches >1 Mbp read length, and can be easily performed in any laboratory (Pucker et al., 2022).

PacBio (HiFi) or ONT sequencing, combined with proximity information obtained by chromosome conformation capture sequencing (e.g., Hi‐C), has started to become prevalent for chromosome‐scale, high‐quality genome assemblies for model and non‐model plant species (e.g., Hoencamp et al., 2021; Mascher et al., 2017; Qu et al., 2023; Schwacke et al., 2025; Xie et al., 2020). The quality and completeness of the assembly can be improved by polishing strategies using available, quality‐filtered Illumina, ONT, or PacBio reads (e.g., Chang et al., 2023; Dmitriev et al., 2021; Zimin & Salzberg, 2020). The combination of low‐ to medium‐sequencing coverage long‐read data with highly accurate, often already available Illumina sequences can be particularly attractive for improving genome assemblies when resources are limited.

Producing “high‐quality” genome sequences, that is, genome assemblies characterized by >90% completeness with respect to expected genome size, >1–10 Mbp contiguity (N50), and >90% recovery rate of conserved protein‐coding genes (BUSCO; see assembly standards of the Earth Biogenome Project, Lawniczak et al., 2022), represents an important target for research groups. However, some metrics are rather adapted for animals. For example, flowering plants have undergone many ancient or recent polyploidization events (Hörandl, 2022; Leebens‐Mack et al., 2019; Van De Peer et al., 2017) and are characterized by quite large, repetitive genomes with duplicated sequence content. Consequently, a BUSCO “embryophyta_odb10” score of >90% single‐copy genes can often not be met because genes are often duplicated in the genome (Dmitriev et al., 2021), or it is not informative for quality assessment in non‐coding, repetitive DNA regions (e.g., long terminal repeat [LTR] assembly index; Mokhtar et al., 2023). Irrespective of chosen metrics, efficient strategies in terms of laboratory effort, cost, and assembly quality are needed to reliably reconstruct large and complex high‐quality genome sequences of non‐model plants. These high‐quality genomes are specifically necessary to study phylogenomic, biogeographic, and ecological relationships within groups characterized by intricate evolutionary processes (Ennos et al., 2005; Karbstein et al., 2024).

*Ranunculus* represents a cosmopolitan genus with approximately 600 species, of which about 40% are polyploid (Hörandl et al., 2005). The *Ranunculus auricomus* complex is one of the largest polyploid apomictic plant species groups in Eurasia, with more than 840 described apomictic taxa (Hörandl & Raab‐Straube, 2015). The group is an important system for understanding the evolution and biogeography of young flowering plants formed by hybridization or allopolyploidy (Bradican et al., 2023, 2024; Hodač & Karbstein et al., 2023; Hörandl et al., 2009; Karbstein et al., 2022; Paun et al., 2006; Tomasello et al., 2020). It has also been frequently studied for the cytogenetic, environmental, and evolutionary mechanisms of apomixis (that is, asexual reproduction via seeds; Hörandl et al., 2024). The *R. auricomus* complex contains five newly circumscribed, less than 830 kyr‐old, and predominantly diploid sexual progenitor species (Karbstein, Tomasello, et al., 2020; Tomasello et al., 2020). The diploid genome size (2n = 16) ranges between 6.12 and 6.30 pg DNA (recalculated as 5.998–6.174 Gbp; Hörandl & Greilhuber, 2002; Leitch et al., 2019). Therefore, *R. auricomus* genomes are large even at the diploid level. Increasing proportions of repetitive regions are responsible for genome size inflation in plants (Bennetzen, 2005; Guo et al., 2021; Jayakodi et al., 2023; Peška et al., 2019), which could also be relevant for *R. auricomus* genomes.

However, so far, gene‐annotated reference genome sequences for the species complex and for the entire genus *Ranunculus* are missing. In Ranunculaceae, the only other reference genome sequences are available from diploid *Aquilegia* and *Coptis* species (Friedhoff et al., 2024; Liu et al., 2021; Mokhtar et al., 2023; Xie et al., 2020), but these genomes are much smaller (<1 Gbp) and polyploid species complexes have not been reported in these genera (TROPICOS database v3.4.2, http://www.tropicos.org; CCDB database v1.66, https://ccdb.tau.ac.il; Rice et al., 2015).

A gene‐annotated reference genome sequence of diploid *Ranunculus* would be a major step forward in improving the reconstruction of the species relationships by facilitating allele phasing and subgenome assignments in hybrids for multilabeled species trees (e.g., Dauphin et al., 2018) or allopolyploid network reconstructions (e.g., Jones, 2017; Šlenker et al., 2021). Further, a reference genome assembly would enable comparative genomic analyses of nucleotide sequence and structural variation among *Ranunculus* species and to study the origin and regulatory mechanisms of apomixis (e.g., Brukhin et al., 2019). Organellar genome reconstructions are also valuable targets for studying gene loss in evolutionary contexts, nuclear‐organelle compatibility, or the chimerism of cyto‐nuclear complexes (Sloan et al., 2018). Little is known, for instance, about the evolution of plant mitochondrial genomes, although mitochondrial‐nuclear compatibility is important for speciation, the rise of crossing barriers, and the emergence of cytoplasmic male sterility (Havird et al., 2015; Hörandl, 2024; Møller et al., 2021; Park et al., 2021; Postel & Touzet, 2020). In *Ranunculus* and Ranunculaceae, such large‐scale organellar genome studies are missing.

Here, we present gene‐annotated organellar and nuclear genome assemblies of the diploid species *R. cassubicifolius* for use in comparative analyses across the Ranunculales. Different assembly and polishing strategies were applied using short‐ and long‐read DNA sequencing technologies, with the best nuclear genome assembly being chromosome‐scaffolded. These genomic resources will be foundational for future work at the species complex, genus, and family level.

## RESULTS AND DISCUSSION

More than 504 million filtered Illumina reads (150 bp) were generated of *R. cassubicifolius*, resulting in 70.7 Gbp raw data and a genome coverage of 22× (mean base‐call accuracy of 99.97%), assuming a haploid genome size of 3.2 Gbp. The ONT long‐read dataset contains 51.6 Gbp and 5.83 million reads (N50 = 22.6 kbp), yielding a coverage of 16× (96.62%). The final PacBio (HiFi) long‐read dataset harbors 79.1 Gbp and 4.40 million reads (N50 = 17.9 kbp), resulting in a coverage of 25× (99.98%).

### Plastome features and gene evolution within *Ranunculus* and Ranunculaceae

**Figure 1:**
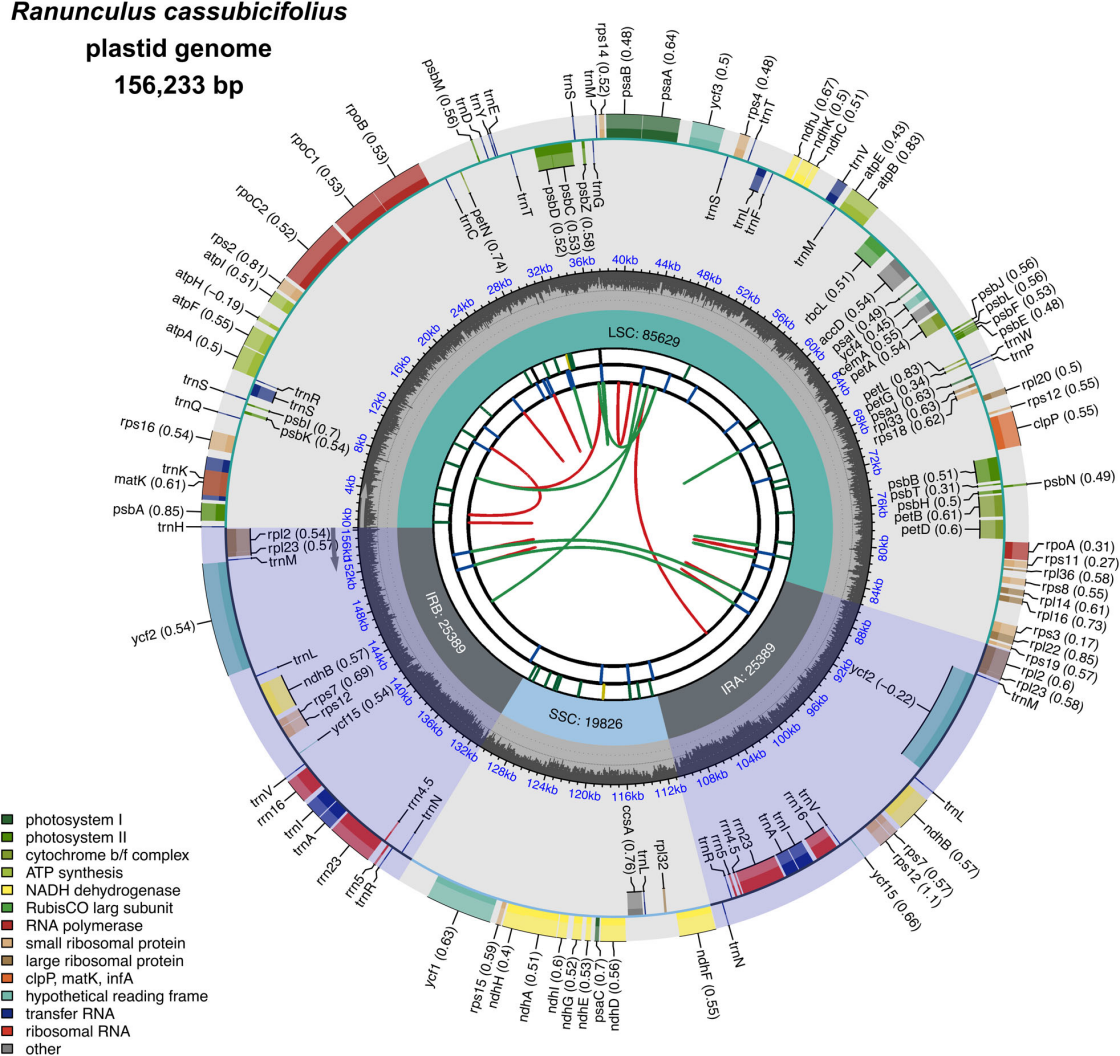
The annotated plastome sequence of *Ranunculus cassubicifolius*. The plastome assembly is a 156 233 bp‐long single circular contig. It contains 85 (78 unique) protein‐coding genes, 37 (29 unique) tRNAs, and 8 (4 unique) rRNAs, as shown in the outer circle. Genes and t/rRNAs on the inside and outside of the circle are transcribed in the clockwise and counterclockwise directions, respectively. The gene codon usage bias derived from CPGView is given in brackets. The first inner circle represents GC content (%), and the second inner circle the plastome structure, that is, a small single‐copy region (SSC) and a large single‐copy region (LSC) separated by two inverted repeat (IRA/B) regions. The third inner circle reflects repeat content, that is, microsatellites (1–6 bp per repeat), tandem repeats (>6 bp per repeat), and dispersed repeats (transposable elements or satellite DNA). See Shi et al. (2019) for more details.

**Figure 2:**
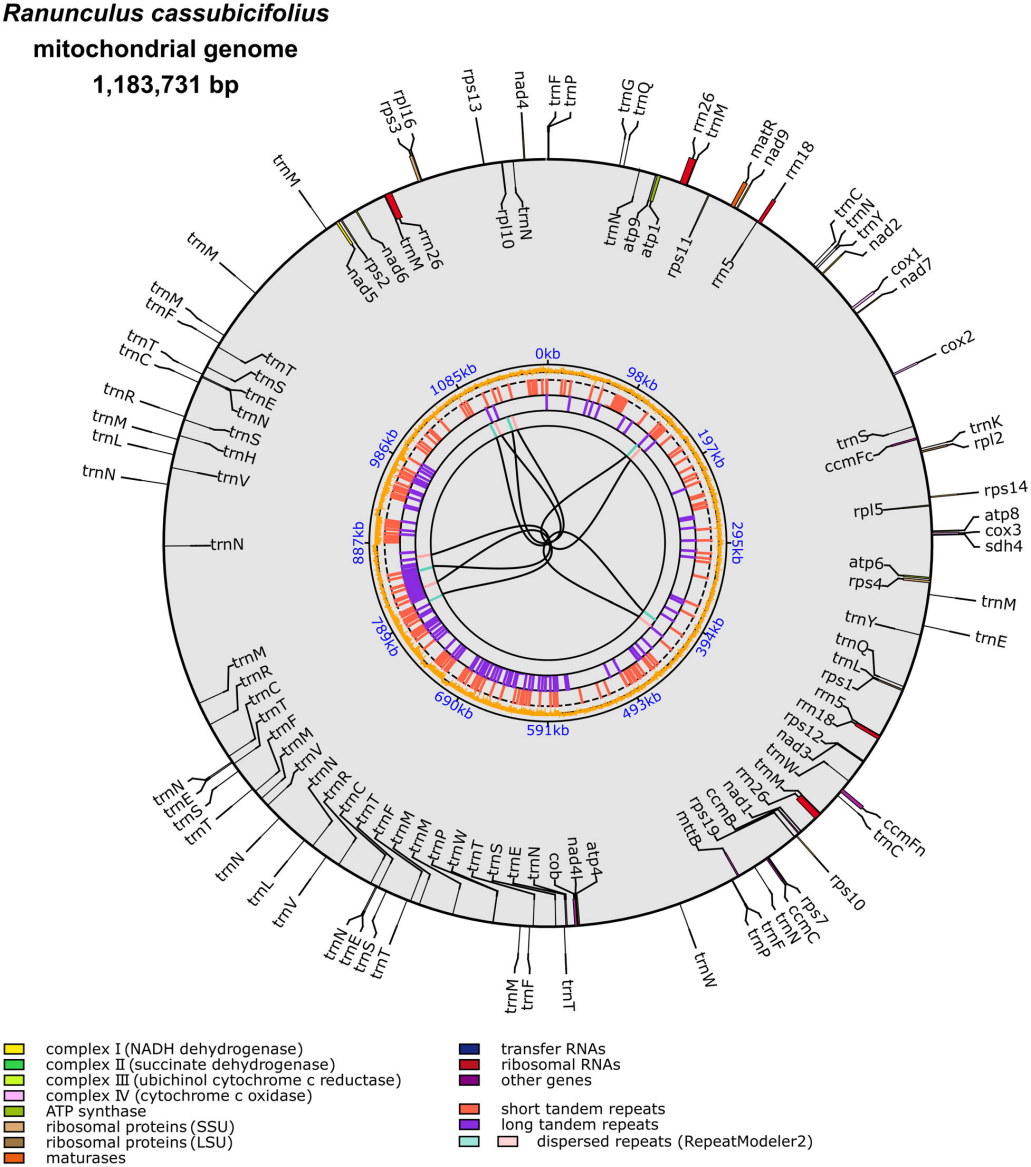
The mitogenome sequence of *Ranunculus cassubicifolius*. The mitogenome is 1 183 731 bp in size represented in a single, circular contig, and contains 40 (40 unique) protein‐coding genes, 77 (unique 18) tRNAs, and 7 (unique 3) rRNAs, as shown in the outer circle. Genes and t/rRNAs on the inside and outside of the circle are transcribed in the clockwise and counterclockwise directions, respectively. The first inner circle represents GC content (%). The subsequent inner circles reflect repeat content, that is, short tandem repeats/microsatellites (1–6 bp per repeat), long tandem repeats (>6 bp per repeat), and dispersed repeats detected by RepeatModeler2. See Zhang, Chen, et al. (2024) for more details.

Based on Illumina reads, we retained a circular plastid genome sequence with 156 233 bp (Figure 1, see mitogenome in Figure 2, Table 1). It shows a typical tetrapartite structure of a small single‐copy (SSC, length: 19 826 bp) and a large single‐copy region (LSC, length: 85 629 bp) separated by two inverted repeat regions (IR, length: 2 × 25 389 bp). The plastome contains 85 (78 unique) protein‐coding genes, 37 (29 unique) tRNAs, and 8 (4 unique) rRNAs, resulting in a coding content of 53.2%. In comparison with closely related *R. sceleratus* and more distantly related *R. repens*, *R. cassubicifolius* shows a similar number of unique protein‐coding genes (78/77/78), tRNAs (29/28/29), and rRNAs (4/3/4). Slight plastome size differences among these species, but also among examined Ranunculaceae accessions, are mainly due to mono‐, oligo‐, or polynucleotide insertions or deletions between protein‐coding genes (e.g., at 15 754 bp or 126 944 bp in the alignment). Within Ranunculaceae, *R. cassubicifolius* is quite average, considering the longest (167 kbp; *Helleborus atrorubens*) and shortest (150 kbp; *Myosurus apetalus*) available plastome sequences. A clear phylogenetic trend of genome size evolution in Ranunculaceae has not been identified.

**Table 1:** Assembly statistics of the annotated plastome, mitogenome, and chromosome‐scaffolded nuclear genome sequences of *Ranunculus cassubicifolius*

Metric	Plastome	Mitogenome	Nuclear Genome
Assembly strategy	Illumina (GetOrganelle)	Illumina (filtered by GetOrganelle) assembled with previously mapped HiFi reads (MaSuRCA)	*Best strategy*: PacBio (Hifiasm) + 3× Polishing [HiFi (Racon), HiFi (polishCLR), Illumina (POLCA)] + Hi‐C chromosome‐level scaffolding
Genome size	156 233 bp	1 183 731 bp	2 691 150 139 bp
Number of Contigs	1 (circular)	1 (circular)	8 (pseudochromosomes)
N50 of Contigs	—	—	355 Mbp
Largest Contig	156 233 bp	1 183 731 bp	447 Mbp
BUSCO Genes (S+D / S)	—	—	94.1% / 78.8%
Protein‐coding genes	85 (78 unique)	40 (40 unique)	35 482 (31 322 unique)
tRNA genes	37 (29 unique)	77 (unique 18)	984
rRNA genes	8 (4 unique)	7 (unique 3)	6448
GC‐content (%)	37.9%	43.5%	42.1%
Repeat content (%)	2.15%	2.13%	86.0%
Gene content (%)	53.2%	7.99%	4.1%

Statistics were calculated with Geneious and R. See Table 2 for a detailed report on repeat content of the nuclear genome, and the Methods and Results sections for more details.

**Table 2:** Repeat content of the chromosome‐scaffolded nuclear genome sequence of *Ranunculus cassubicifolius*

Class	Total sequences	Total length	%Masked
	320	2 691 150 139	
	Count	bpMasked	
Class I (retrotransposons)			
LINE			
L1	31 842	21 678 526	0.81%
LTR			
Copia	147 394	80 810 425	3.00%
Gypsy	1 105 626	654 391 560	24.3%
Unknown	2 075 881	1 353 935 235	50.3%
SINE			
tRNA	309	114 573	0.00%
Unknown	347	444 295	0.02%
Class II (DNA transposons)			
TIR			
CACTA	85 851	32 242 719	1.20%
Mutator	134 007	38 209 632	1.42%
PIF_Harbinger	86 910	30 275 684	1.13%
Tc1_Mariner	32 184	12 038 336	0.45%
hAT	133 119	40 436 252	1.50%
nonTIR			
Helitron	123 174	38 735 549	1.44%
rDNA			
45S	191	42 204	0.00%
Repeat fragment	44 541	11 822 897	0.44%
Satellite DNA	186	83 887	0.00%
Total	4 001 562	2 315 261 774	86.0%

Long Interspersed Nuclear Elements (LINEs), Short Interspersed Nuclear Elements (SINEs), Long Terminal Repeats (LTRs), and Terminal Inverted Repeats (TIR). A detailed explanation of repeat classes can be found in Wicker et al. (2007). Annotation reports are deposited in GitHub (https://github.com/NancyChoudhary28/Ranunculus‐genomics).

**Figure 3:**
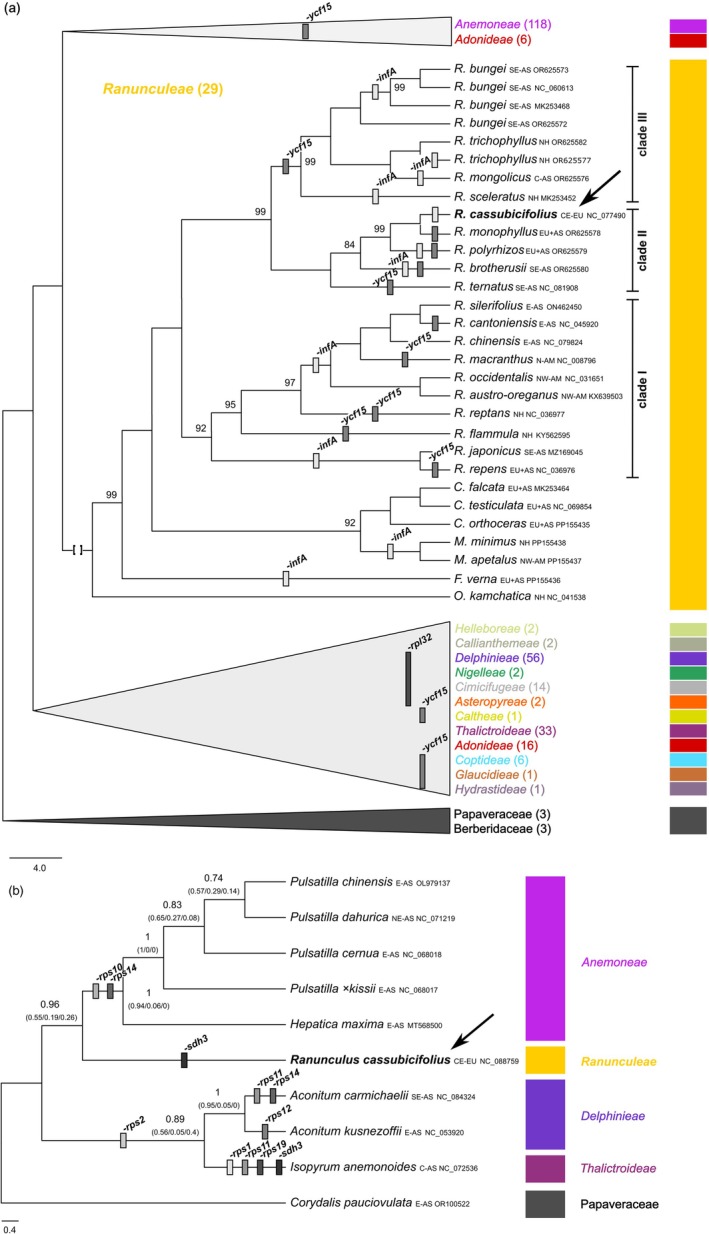
Phylogenetic relationships and gene evolution within Ranunculaceae. (a) MaxLik phylogeny based on 308 plastome sequences (295 taxa) and the min90 alignment (126 kbp). Branches of tribes except for *Ranunculeae* were collapsed, and gained or lost genes are shown in different colored gray bars along the tree. See also the complete phylogeny and Quartet Sampling (QS) metrics in Figures S3a,b and S4. All branches received full (100) Felsenstein bootstrap proportion (FBP) values, unless otherwise shown. (b) Coalescent‐based phylogeny of 10 mitogenome sequences and 42 genes of Ranunculaceae (see Figure S6 for the concatenation‐based phylogeny). Local posterior probabilities (PP) are shown with quartet support values in brackets (*q*_1_ = main topology, *q*_2_ = first alternative, and *q*_3_ = second alternative). All NCBI species names were checked by gbif.org, and synonyms were replaced by accepted names. The color of clades corresponds to tribe/subfamily names (tribal classification follows Wang et al., 2016; Zhai et al., 2019). *Ranunculus cassubicifolius* is highlighted in bold with an arrow. Abbreviations for native distribution ranges of taxa: AM, America; AS, Asia; C, Central; E, Eastern; EU, Europe; N, Northern; NH, Northern Hemisphere; S, Southern; and W, Western.

The Maximum Likelihood (MaxLik) tree, including 292 taxa, shows a well‐supported phylogeny of the Ranunculaceae (TBE ~85‐100%; Figure 3a, Figure S4, Text S4). Low branch support is seen within species‐rich genera such as *Anemone* or *Aconitum* and is probably due to fast radiation and the slow evolutionary rates of plastomes (Wang et al., 2016; Zhai et al., 2019). Almost all subfamilies or tribes are monophyletic, and branching is widely consistent with previous studies on plastomes (35 taxa) or nuclear and plastid loci (105 taxa; Wang et al., 2009; Zhai et al., 2019). Within *Ranunculeae*, the genera *Oxygraphis*, *Ficaria*, *Myosurus*, and *Ceratocephala* represent close relatives of *Ranunculus*, in agreement with Emadzade et al. (2010). Compared to Zhai et al. (2019) and Ji et al. (2023), the plastome phylogeny presented here is the most up‐to‐date and taxon‐rich for Ranunculaceae. *Ranunculus* is split into a Eurasian‐American (clade 1), European–Asian clade including *R*. sect. *Auricomus* (clade 2), and a predominant Asian clade including aquatic taxa (clade 3). The phylogeny is also largely congruent with Hörandl and Emadzade (2012), but based on a smaller taxon sampling.

Regarding gene evolution within Ranunculaceae, particularly *Ranunculus*, we observed that *infA*, *pbf1*, *rpl32*, *rps16*, or *ycf15* have frequently been lost in the examined plastome sequences (Figure 3a; feature tables on FigShare [Item 02]). The plastid gene *infA* is present in all tribes of Ranunculaceae but appears to have been lost in 9 out of 23 (39%) examined *Ranunculus* accessions, including *R. cassubicifolius*. Loss and transfer of plastid genes to the nuclear genome are documented for various angiosperm lineages, making *infA* one of the most mobile plastome genes in plants (Millen et al., 2001). In the case of *R. cassubicifolius*, we were able to confirm the loss of *infA* and its transfer to the nuclear genome (two presumably functional, ca. 150–160 bp size reduced copies exist at Chr1 and Chr4), a result not previously reported. The gene *infA* is important for ribosome assembly, translation initiation, and cell viability (Cummings & Hershey, 1994). Transfer to the nucleus may reduce the metabolic burden of the chloroplast or enhance the functional regulation of plastid‐ and nucleus‐encoded proteins; it may also escape from the accumulation of deleterious mutations in organellar genomes of asexual organisms (Millen et al., 2001). The gene *infA*, among others, can play a crucial role in plant adaptation to different temperatures (slipper orchids; Hu et al., 2022). In *R. auricomus*, it may also promote the adaptation of allopolyploid apomicts distributed from Arctic to Mediterranean climates.

Following its loss in *Asteropyreae*, the ribosomal gene *rpl32* is present again in *Callianthemeae* and all more derived clades. Its putative loss and functional transfer to the nuclear genome have been observed in other clades within Ranunculaceae (Park et al., 2015; Park & Park, 2020; Zhai et al., 2019). The gene *rpl32* may be involved in rapid adaptation to novel environments, such as in *Myosurus* shifting from mesophytic to wet habitats during climate change over the last 5 Ma (Long et al., 2024). In contrast to *Myosurus*, where *rpl32* is reduced to 138 bp, the gene shows a typical length of 162 bp in *R. cassubicifolius*, suggesting that it is probably not under strong selective pressure.

The gene *ycf15* appears also to have been frequently lost in the evolution of Ranunculaceae (e.g., in *Hydrastideae,* *Caltheae*, or *Anemoneae*), and in 10 out of 23 (43%) examined accessions in *Ranunculus* including *R. cassubicifolius*. For the genus *Ranunculus*, the complete loss of this gene had been assumed, but the results presented here contradict this assumption. Although the gene is only 54 bp long and apparently degraded in *R. cassubicifolius* (e.g., compared to 198 bp in *R. austro‐oreganus*), and seemingly without essential function to survival (Li et al., 2021; Shi et al., 2013), we found two copies of *ycf15* in the IR regions with regular start and stop codons. The function of *ycf15* remains unclear; however, the co‐transcribed *ycf2* and *accD* influence plastid structure and competitiveness and cyto‐nuclear coevolution, which in turn may favor the ability for hybridization (Drescher et al., 2000; Liu et al., 2017; Postel & Touzet, 2020; Shi et al., 2013; Sobanski et al., 2019). Its role in the hybridization of sexual progenitors of *R. auricomus*, which produced hundreds of apomictic taxa, requires further investigation.

Other plastid genes frequently known to be transferred into the nuclear genome like *accD, ycf1*, or *ycf2* are also found with regular start codons and similar sizes in the nuclear genome of *R. cassubicifolius* (e.g., at different positions of Chr1). Noteworthy, we generally observed that annotations of (pseudo)genes are sometimes missing in published plastomes (e.g., *clpp*, *psbG*, *rpl22*, or *rpl32*), which complicates gene comparisons. We thus recommend careful annotation of plastome sequences through cross‐species alignments of homologous genes and better quality control of online databases during the genome submission steps.

### Mitogenome features and gene evolution within *Ranunculus* and Ranunculaceae

Based on the Illumina‐PacBio assembly, we retrieved a mitochondrial *R. cassubicifolius* genome sequence of 1 183 731 bp (Figure 2, Table 1). It contains 40 (40 unique) genes, 77 (18 unique) tRNAs, and 7 (3 unique) rRNAs, resulting in a coding content of 7.99%. The mitogenome of the closest available reference *Hepatica maxima* shows similar numbers of genes (40/39), t‐RNAs (18/18), and rRNAs (3/3), but is slightly shorter (‐61 185 bp). In Ranunculaceae, variation in mitogenome size is high, ranging from 0.207 Mbp in *Isopyrum anemonoides* to 1.184 Mbp in our species. In angiosperms, mitogenomes can even range from 66 kbp to 11.7 Mbp (Putintseva et al., 2020; Skippington et al., 2015). Our mitogenome is thus relatively small, but one of the largest known for Ranunculaceae.

Size differences in Ranunculaceae can only be explained to a limited extent by variation in repeat content. In *R. cassubicifolius*, 2.1% repeat content was detected. This is lower than in *H. maxima* (6.9%) and substantially lower than in *I. anemonoides* (56%), which has the smallest known mitogenome. Unlike plastomes, plant mitogenomes are recombinationally active, can exist as alternative structures even within individuals, and can be organized in noncircular molecules (Møller et al., 2021; Sloan, 2013). Sequence differences, despite similar gene content, largely resulted from recombination and rearrangements in non‐coding regions (Figure S5a). Structural differences between PacBio and ONT assemblies (Figure S5b) may reflect sequencing of different individuals rather than differing technologies.

The coalescent‐based mitogene phylogeny of Ranunculaceae was well‐supported (local posterior probabilities ~0.9–1, Figure 3b), marking the first one for the family and one of the first for plants (e.g., Lin et al., 2022). Like in the plastome tree, low branch support occurs especially within the fast‐radiating, species‐rich clade *Anemoneae*. Branching of infrageneric taxa (*Thalictroideae*, *Delphinieae*, *Ranunculeae*, and *Anemoneae*) is also consistent with the plastome MaxLik trees (Figure 3a; Wang et al., 2009; Zhai et al., 2019). Regarding gene evolution within Ranunculaceae, we observed that the mitogenes *rps1*, *rps2*, *rps10*, *rps11*, *rps12*, *rps14*, *rps19*, or *sdh3* were occasionally absent (Figure 3b; see feature table on FigShare [Item 03]). For *rps* genes and *sdh3*, loss and transfer to the nuclear genome have been frequently observed in flowering plants (Adams et al., 2000, 2001; Bi et al., 2020).

In our study, the genes *rps2* and *rps11* were not found in *Isopyrum anemonoides* and *Aconitum carmichaelii*, and *rps10* and *rps14* appeared to be lost in the examined species of *Anemoneae*, and putatively transferred to the nuclear genome (no nuclear genomes available). The *rps* genes are essential for mitochondrial ribosome function, as well as for vegetative and reproductive plant development (Adams et al., 2000, 2001; Robles & Quesada, 2017). These genes have been found to be under positive selection in certain plant groups (e.g., *rps1*, *rps10, rps14* in legumes: Bi et al., 2020; or *rps13* in rosids: Liu & Adams, 2008).

Most mitochondrial genes have already been transferred to the nuclear genome in plants. This enhances regulatory host control and has important consequences for ATP synthesis, metabolism, and (a)biotic stress responses (Adams et al., 2000; Bonen & Calixte, 2006; Fakih et al., 2023; Saha et al., 2017; Woodson & Chory, 2008). The *rps* genes are known to be regulated under temperature heat and cold stress (Alafari & Abd‐Elgawad, 2021; Fakih et al., 2023). Selfing, in the absence of deleterious mutation accumulation in the mitogenome, is known to increase the gene transfer to the nuclear genome and fix co‐adapted gene combinations (Brandvain & Wade, 2009). Interestingly, only in self‐incompatible *R. cassubicifolius* (Hörandl, 2008; Karbstein, Rahmsdorf, et al., 2020), all *rps* genes are present. In contrast, *Anemone*, *Pulsatilla*, and *Aconitum* are known to be largely self‐compatible (Liao et al., 2009; Lindell, 1998; Zhigang et al., 2006). It would be worth investigating whether the self‐compatible, widely distributed apomicts (Hörandl, 2008; Karbstein et al., 2021) retain as many *rps* genes in their mitogenomes as their sexual *R. auricomus* progenitors, such as *R. cassubicifolius*.

The gene *sdh3* was not detected in *R. cassubicifolius* and *I. anemonoides*. It encodes the subunit 3 of the succinate dehydrogenase and plays a role in electron transfer in complex II of the respiratory chain (Gebert et al., 2011). Loss and/or transfer to the nuclear genome have often been observed (Adams et al., 2001; Choi et al., 2019; Huang et al., 2019). Surprisingly, transfer to the nuclear genome could not be confirmed in *R. cassubicifolius*. Loss of *sdh3* might reflect optimization of cellular efficiency, and that it is not essential in the investigated lineages.

### The nuclear genome features

**Figure 4:**
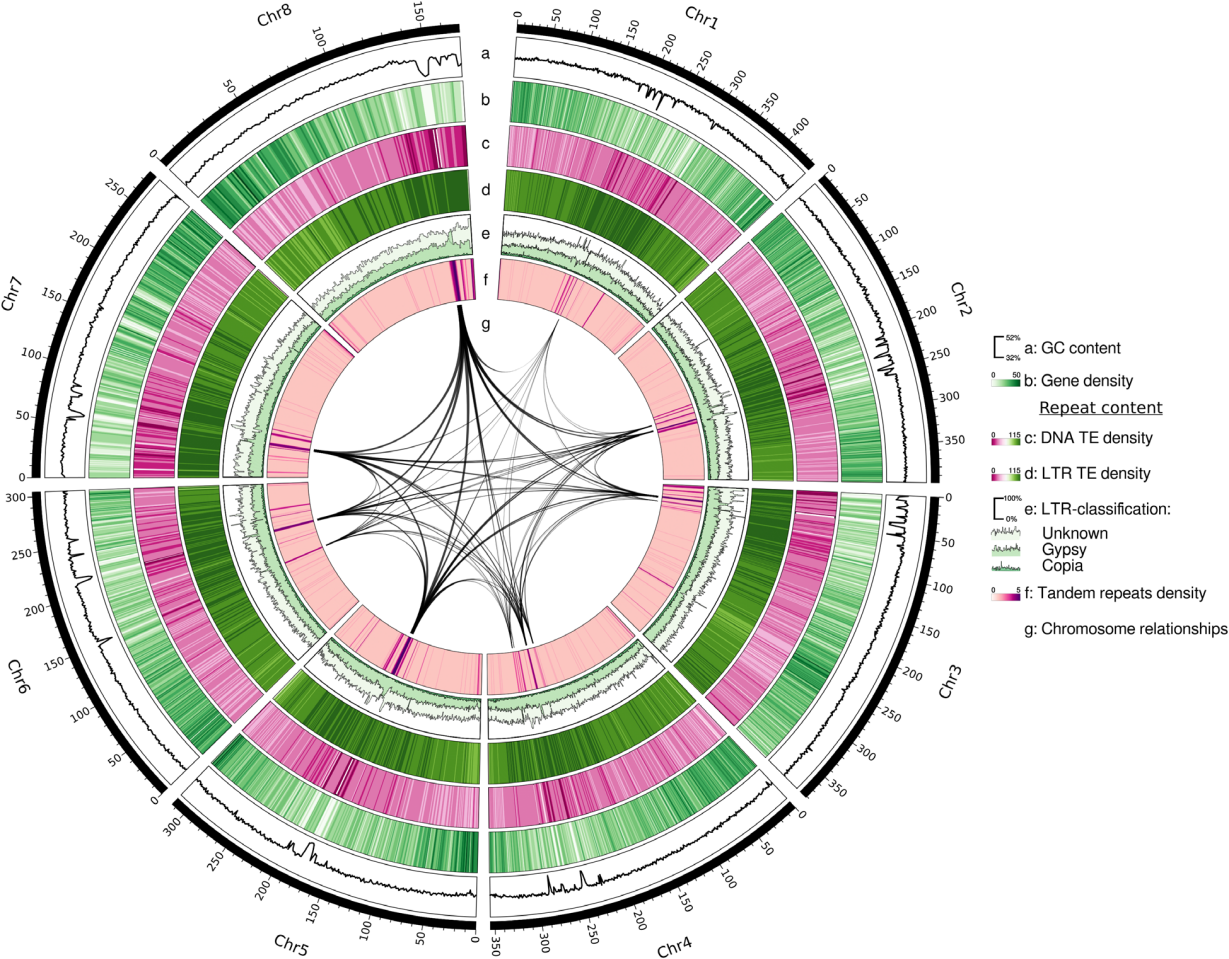
Circos plot of the chromosome‐scaffolded nuclear genome sequence of *Ranunculus cassubicifolius*. The genome is 2.69 Gbp in size, has 8 pseudochromosomes, and 35 482 (31 322 unique) annotated genes. Illustrated are (from the outer to the inner circle): Chromosome number, chromosome size in Mbp, GC content (a), protein‐coding gene density (b), DNA transposon element density (c), long terminal repeat (LTR) retrotransposon density (d), percentage of unknown, Gypsy, and Copia LTR‐type TEs (e), tandem repeat density (f), and major interchromosomal synteny (g).

Based on the best PacBio assembly, we obtained a nuclear genome sequence of 2 691 150 139 bp (Tables 1 and 3). Ca. 98.4% (2.65 Gbp) of the scaffolds were anchored into eight pseudochromosomes using Hi‐C data. The assembly has an N50 of 355 Mbp, with the longest chromosome spanning 447 Mbp (Figure 4), and 94.1% (78.8% single copy, 15.3% duplicated) BUSCO genes present. It exceeds the recommended standard of the Earth Biogenome Project in terms of continuity (scaffolds >10 Mbp), functional completeness (>90% BUSCO genes), and chromosome status (>80% contigs assigned to chromosomes; Lawniczak et al., 2022).

**Table 3:** QUAST statistics for different long‐read genome assembly strategies of *Ranunculus cassubicifolius*

Metric	Canu (ONT 16× / PacBio 16×)	Canu (ONT 16× / PacBio 16×) + 3× Polish	Flye (ONT 16× / PacBio 16×)	Flye (ONT 16× / PacBio 16×) + 3× Polish	Hifiasm (PacBio 25× /PacBio 16×)	Hifiasm (PacBio 25×) + 3× Polish
Total assembly length (genome completeness), Mbp	0.80 (25%) / 5.05 (158%)	0.81 (25%) / 5.04 (157%)	3.58 (112%) / 4.90 (153%)	3.45 (108%) / 4.90 (153%)	3.22 (101%) / 3.62 (113%)	3.21 (100%)
No. contigs	30 612 / 16 262	32 959 / 15 711	65 232 / 12 514	54 310 / 12 356	1 453 / 3 449	1319
No. contigs >1 Mbp	30 612 / 16 262	31 072 / 15 711	64 618 / 12 506	53 572 / 12 356	1 453 / 3 449	1 319
Largest contig, Mbp	0.540 / 14.3	0.542 / 14.3	0.976 / 6.57	0.974 / 6.57	84 / 32	84
GC, %	40 / 42	40 / 42	43 / 42	43 / 42	42 / 42	42
N50, Mbp	0.037 / 0.617	0.038 / 0.618	0.103 / 0.853	0.104 / 0.852	20.0 / 2.89	20.0
L50	6935 / 2374	6937 / 2366	10 305 / 1728	9889 / 1727	45 / 350	45

Canu, Flye, and Hifiasm used ONT (LH040/02) or PacBio (LH040/06) long read data. Coverage is given per read type. Polishing included Racon, Medaka / polishCLR, and POLCA steps. N50 describes the shortest contig length required to summarize at least half (50%) of the bases of the entire assembly; L50 is defined as the minimum number of contigs that encompass half (50%) of the bases of the entire assembly. See Tables S4 and S5 for short‐ and hybrid‐read assemblies based on 16× vs. 25× PacBio data.

The nuclear genome sequence contains 35 482 (31 322 unique) protein‐coding genes with an average and coding sequence length of 3548 bp and 1169 bp, respectively. Analyses predicted 984 tRNA, 99 spliceosomal RNA, and 6448 rRNA gene models. Compared to *T. thalictroides* (243 Mbp), *Aquilegia kansuensis* (293 Mbp), *A. coerulea* (293 Mbp), *Coptis chinensis* (959 Mbp), and *Coptis teeta* (932 Mbp), the *R. cassubicifolius* genome is up to 11‐fold larger (+1.78–2.92 Gbp) but shows a similar chromosome count (8 vs. 7 or 9). *Ranunculus* has mostly the long, strongly winding R‐type chromosomes of the family (Tamura, 1993). Our target species exhibits an ancestral karyotype with Chr1, Chr2, Chr5, and Chr6 as four metacentric and Chr3, Chr4, Chr7, and Chr8 as four submetacentric‐subtelocentric chromosomes (visualized in ModDotPlots, Figure S8a–h; Baltisberger & Hörandl, 2016). In angiosperms, genome size varies from 0.063 to 149 Gbp (median 0.6 / mean 5.7 Gbp, Dodsworth et al., 2015). The *R. cassubicifolius* genome is among the larger ones in flowering plants and the largest yet assembled in Ranunculaceae. Genome sizes in diploid *Ranunculus* species range from 1.86 to 8.43 Gbp based on karyological estimates (Leitch et al., 2019). The genome of *Ranunculus cassubicifolius* is thus of medium size compared to other species in the genus.

**Figure 5:**
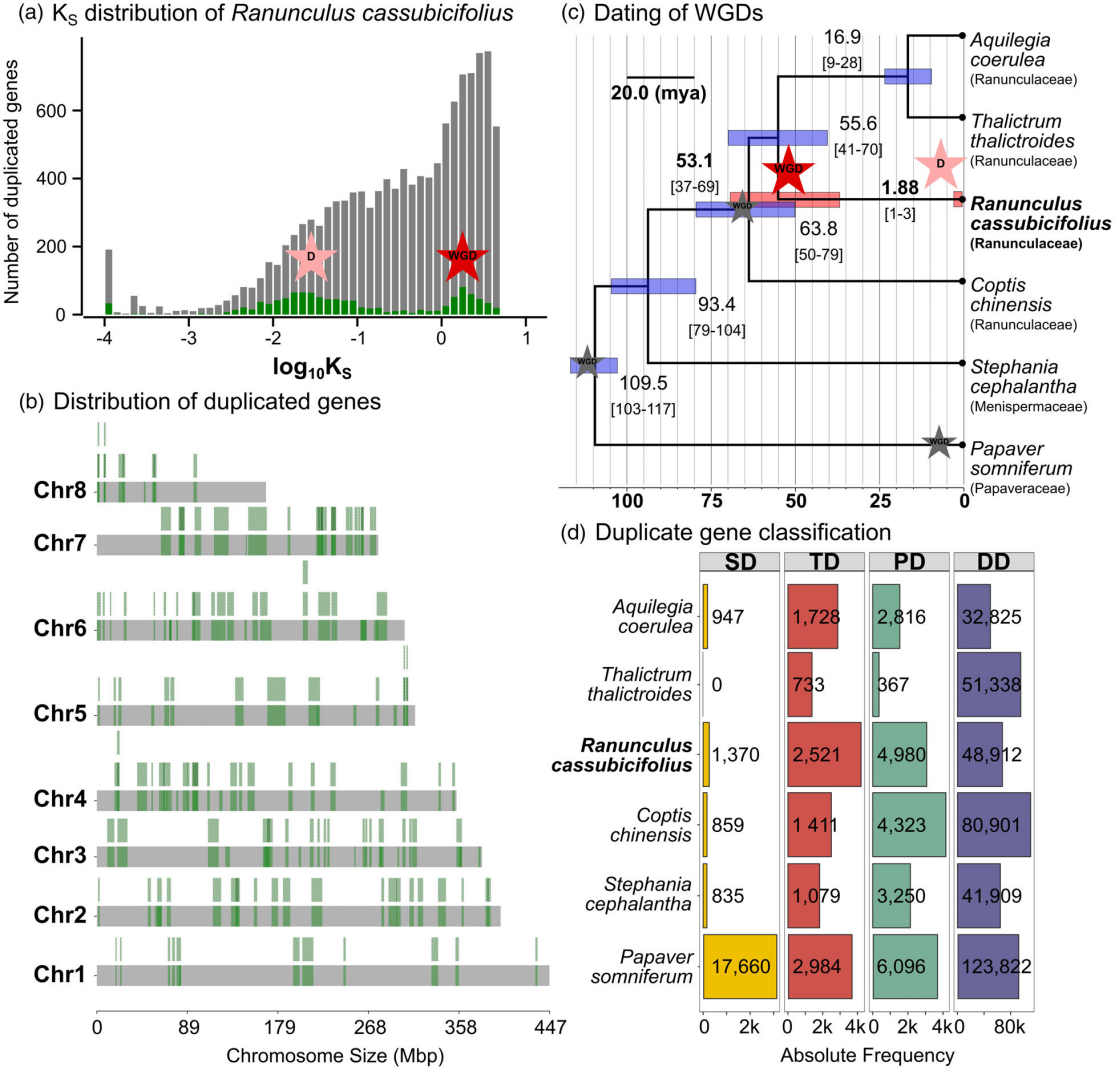
Detection of WGD events in *Ranunculus cassubicifolius*. While the ancient WGD is supported by other studies, the more recent duplication (D) likely results from small‐scale duplications. (a–c) wgd v2: (a) Anchor K_S_ distribution (x‐axis) of duplicated/paralogous genes (y‐axis) indicates a more recent (left peak) and a more ancient (right peak) duplication event. *K*_S_, synonymous substitutions per site; anchor pairs, pairs of duplicates from a WGD event (identified in syntentic blocks) are highlighted in green. (b) Gene synteny inference: the dupStack plot shows the distribution of duplicated gene segments (anchor pairs) across the chromosomes. Many regions were characterized by duplicated collinear segments, sometimes by triplicates (highlighted in green) in *R. cassubicifolius*. (c) Inferred dates of WGD/D events based on the peptide concatenation‐based dating and the coalescent‐based starting tree including secondary calibration points are indicated by red/light red stars. The black arrow indicates that the inferred WGD is shared by *Coptis*, *Ranunculus*, *Thalictrum*, and *Aquilegia* (see details in Figure S9a,b for interspecies K_S_ and syndepth plots). WGD events from previous studies cited in main text are indicated by gray stars. Confidence intervals of mean time estimates are highlighted by light blue or red bars. Divergence times and interval estimates were averaged for the two duplication events. Branch lengths indicate divergence times in mya. Doubletrouble: (d) Bar plot showing classified paralogous genes of the investigated Ranunculales species. Duplications (D) were classified into segmental duplications (SD) and small‐scale duplications (SSD), that is tandem (TD), proximal (PD), or dispersed duplications (DD). See FigShare [Item 04] for all results, and the Results and Discussion section for more details.

In ancestral angiosperms, a baseline of 12 000–14 000 genes is postulated (Sterck et al., 2007). In Guo et al. (2018), Liu et al. (2021), He et al. (2022), and Zhang, Ge, et al. (2024), ancient whole genome duplication (WGD) events were detected at the base of Ranunculales and Ranunculaceae, as well as tribe‐specific ones (e.g., *Delphinieae*). Our wgd v2 analysis based on gene regions that likely represent duplicated blocks retained after a WGD and a dated phylogenomic tree of six Ranunculales genomes also detected signatures of WGD events in *R. cassubicifolius* (Figure 5a–c, intermediate results on FigShare [Item 04]). The more ancient WGD occurred 36.8 to 69.1 mya (mean 53.1 mya) and is likely shared with *Aquilegia*, *Thalictrum*, and *Coptis* as indicated by the larger Ks anchor pair peak of *R. cassubicifolius* compared to the other Ranunculaceae species (Figure 5a, Figure S9a). The peaks represent the synonymous substitutions (*K*_s_) between anchor gene pairs, which are duplicated genes retained in syntenic blocks indicative of a WGD event. Also, interspecies syndepth plots (depth of synteny between two genomes) supported this by showing low levels of two corresponding blocks (homeologs, 2:2) for *Ranunculus*:*Aquilegia*, *Ranunculus*:*Thalictrum*, and *Ranunculus*:*Coptis* chromosome segments. In contrast, *Stephania* exhibits slightly lower levels, and no triplicated segments (Figure S9b). Consequently, the inferred WGD event likely occurred before the split between *Ranunculus* and *Coptis* (mean 63.8 mya, Figure 5c) and is in line with the phylotranscriptome study of He et al. (2022) that estimated a WGD event 66.7 mya at the base of Ranunculaceae. The more recent duplication happened 1.88 mya [0.87, 3.0] before the origin of the *R. auricomus* species complex (Tomasello et al., 2020) and is placed within the diversification time of the Eurasian‐North American clade of *Ranunculus* sect. *Auricomus* (Emadzade & Hörandl, 2011; Hörandl & Emadzade, 2012), representing a new finding for this plant genus.

Nevertheless, *R. cassubicifolius* shows the typical haploid chromosome number for diploids (*n* = 8) in *Ranunculus*, and a relatively low proportion of duplicated BUSCO genes (15%). Consequently, other processes such as small‐scale chromosomal mutations (e.g., segmental duplications, translocations or insertions) due to unequal crossing over or retrotransposon activity due to sudden climatic changes could have generated the pattern obtained. This is corroborated by results from doubletrouble, a tool that identifies and classifies duplicated genes (Almeida‐Silva and Van de Peer, 2025). The analysis shows substantially higher numbers of small (SSD = 56 413) than large‐scale duplications (SD = 1370; Figure 5d). In addition, *R. cassubicifolius* has slightly more SD and small‐scale tandem duplications (TD = 2521) and partly proximal duplications (PD = 4980) compared to the other Ranunculales species likely due to the observed duplications 2 mya. In contrast, the lineage of *P. somniferum*, which underwent a WGD 8 mya, is characterized by extraordinarily high SD (17 660; Guo et al., 2018). To contribute further to this topic, more high‐quality annotated genomes in Ranunculales and *Ranunculus* are needed.

Almost 86% of the *R. cassubicifolius* genome consists of repetitive elements (Table 2), among the highest known in flowering plants (85% in *Zea mays*; Schnable et al., 2009; Wang et al., 2021). LTR‐RTs are most abundant, accounting for up to 78%. Additionally, DNA transposons, long interspersed nuclear elements (LINEs) and short interspersed nuclear elements (SINEs) account for 5.7, 0.81, and 0.02% of the genome, respectively. In angiosperms, genome size correlates with repetitive elements and particularly with LTR‐RT content but not with WGD (Li et al., 2017; Wang et al., 2021). Huge size differences between Ranunculaceae genomes (10‐fold) can be largely explained by variation in LTR‐RT content. In *R. cassubicifolius*, LTR‐RTs account for 78%, whereas they constitute 36% of the small genomes of *Aquilegia kansuensis* and *Coptis teeta* (Wang et al., 2024; Xie et al., 2020), and in *Coptis chinensis*, it is 50% (Chen et al., 2021). Interestingly, LTR‐RT bursts during the past three million years (Pleistocene) likely aided adaptation to climatic variability in angiosperms by introducing novel genomic functions and altering expression patterns (Wang et al., 2021). Progenitor species of *R. auricomus* also speciated by vicariance processes during cold and warm phases 830–580 kya in Europe (Tomasello et al., 2020). Therefore, climatic variability and the associated need for adaptation may be linked to elevated LTR‐RT values in *R. cassubicifolius*.

Given the high completeness of our assembly (94.1% BUSCO genes), it represents an important resource for exploring the genetic background of shifts from sex to apomixis in *R. cassubicifolius* and beyond. The apomixis candidate genes under selection in hybrids (e.g., *ASY1, XRI1*, and *APC1*), recognized by Paetzold et al. (2022), were also detected in the genome of *R. cassubicifolius* at Chr2 and Chr5. Annotated genes will also be an important resource for comprehensive analysis of loci under selection in the genus (e.g., via dN/dS ratios; Pellino et al., 2013), which could give insights into general genome evolution, adaptation, and speciation.

### Comparison of nuclear genome assembly strategies

**Figure 6:**
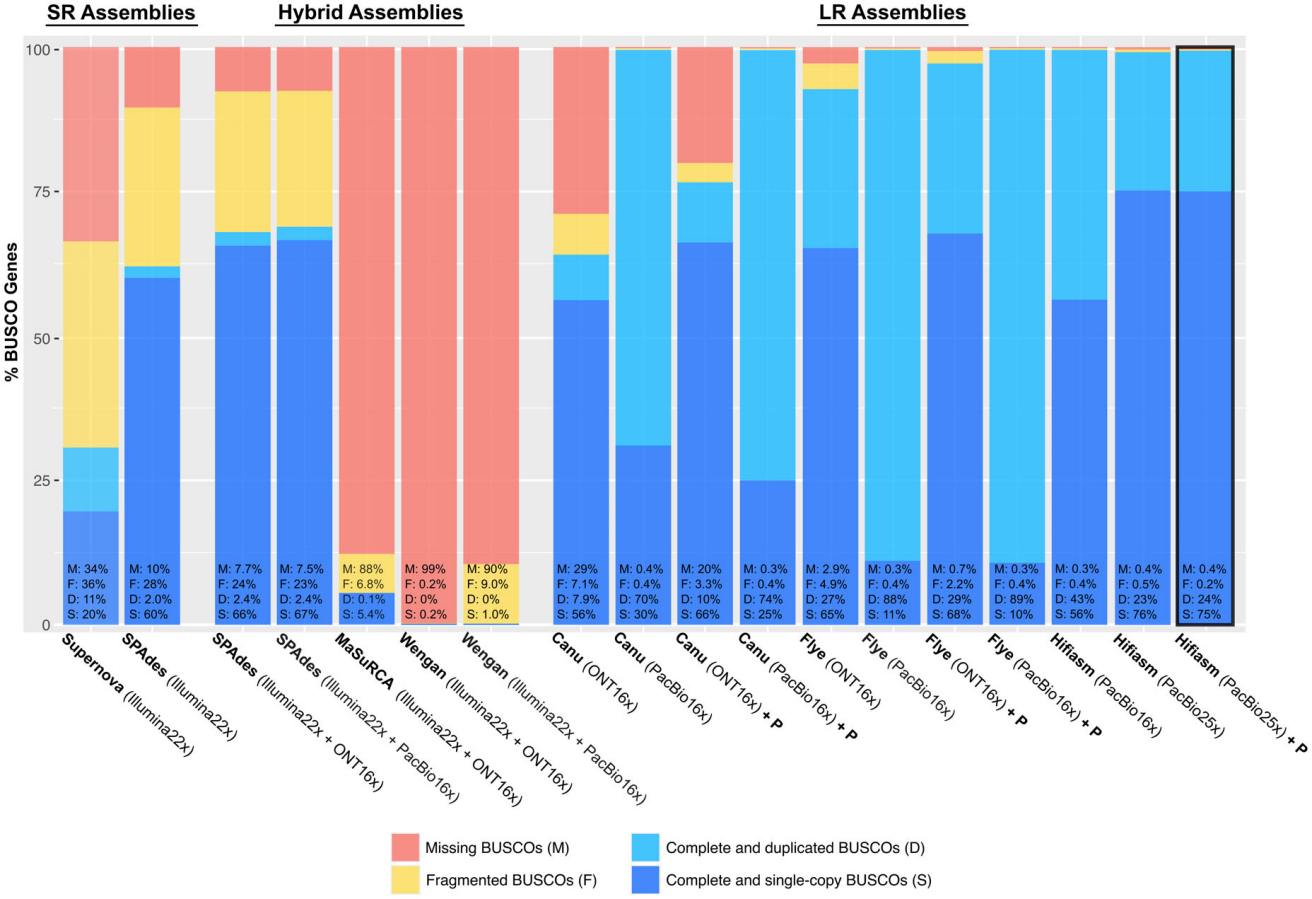
BUSCO assessments for different genome assembly strategies of *Ranunculus cassubicifolius*. Supernova, and partially SPAdes, MaSuRCA, and Wengan used Illumina short‐reads (SR). Canu, Flye, and Hifiasm used ONT/PacBio long‐reads alone (LR), and SPAdes, MaSuRCA, and Wengan used long‐reads in combination with short reads (hybrid strategy). P = polished assemblies. Analyses are based on 1614 BUSCO groups searched. See the Methods section for more details, and Figure S10 and Table S6a–c for 25× PacBio data.

This study presents a systematic and comprehensive comparison of assembly strategies for non‐model plants with large nuclear genomes. Related studies have mainly focused on model plants with high read coverage or a small number of examined tools (e.g., Mascher et al., 2021), or non‐model plants with small‐sized genomes (e.g., Murigneux et al., 2020). Our assembly strategies yielded substantial differences concerning genome completeness, continuity, and quality. Supernova and SPAdes based on Illumina reads delivered low to moderate expected genome completeness (86/17%), with millions of very short contigs (Table S4). BUSCO genes were highly fragmented (F: 36/28%), with many missing genes (M: 34/10%; Figure 6). Our results align with previous studies that tested short Illumina reads for large plant genomes (Gatter et al., 2021; Hotaling et al., 2023; Rhie et al., 2021; Zimin & Salzberg, 2022). Especially in *R. cassubicifolius* with 86% repetitive regions, short reads with moderate coverage cannot resolve long and complex genomic patterns.

SPAdes, MaSuRCA, and Wengan based on Illumina and ONT or PacBio reads performed slightly better in terms of contig length, but showed very low completeness (0.16–13 / 14–38%) and non‐optimal BUSCO scores as many genes were missing (M: 7.7–99 / 7.5–90%, F: 0.2–24 / 9–23%). Hybrid assemblers are considered to be particularly useful under low long‐read coverage (<10–15×), or large or complex genomes (e.g., Di Genova et al., 2021; Gatter et al., 2021; Zimin et al., 2017). However, this cannot be confirmed in our study. These tools were tested on species with small genomes like *Arabidopsis thaliana* (0.135 Gbp, ca. 21% TEs; Quesneville, 2020) or humans (3.2 Gbp, ca. 50% repeats; Liao et al., 2023), or require higher Illumina read coverage (>30–40×) than provided here, which may account for their low performance.

The ONT‐ and PacBio‐based assemblies conducted with Canu, Flye, and Hifiasm were superior in terms of completeness (25–158%), contig number (1453−65 232), contig size (N50: 0.037–20.0 Mbp; Table 3), and quality as the majority of BUSCO genes were found (M: 0.2–29%, F: 0.3–7.1%, D: 7.9–88%, S: 11–76%; Figure 6). This is also consistent with Hotaling et al. (2023), who concluded that long reads allow for assemblies of higher quality, particularly for large repeat‐rich genomes. However, PacBio produced better assemblies than ONT (R9) data at the same coverage when using Canu and Flye. PacBio data delivered the longer and fewer contigs. The lower base accuracy of ONT sequence data (97 vs. 98%), despite a slightly larger sequence length (23 vs. 18 kbp), can explain this observation. The ONT read N50 in this study was ca. 20 kbp, whereas other studies, using fresh libraries or different DNA extraction protocols, achieved over twice this value (Hakim et al., 2024; Horz et al., 2024; Nowak et al., 2024; Text S5). PacBio‐ compared to ONT‐based assemblies generated by Canu and Flye were far too large considering the expected genome size (158/153 vs. 25/112%) and characterized by too many duplicated BUSCO genes (70/88 vs. 8/27%). This suggests uncollapsed heterozygous regions in the assembly.

Hifiasm outperformed Canu and Flye, using either PacBio 25× or 16× downsampled data, in completeness, contig length, contig number (Table 3, Tables S4, S5), and runtime (2–3 vs. 7–14 days), but not in found BUSCO genes (all ca. 99%; Figure 6, Figure S10). This agrees with recent benchmarks on complex genomes (Cheng et al., 2021; Yu et al., 2024). Our polishing procedure slightly improved missing and fragmented BUSCO gene metrics (M: 4.2 vs. 3.2%; F: 1.8 vs. 1.0%), and reduced contig number >1 Mbp (23 206 vs. 21 574) for long‐read assemblies, with the most significant improvement for the ONT‐Canu assembly (S: 66 vs. 56%; D: 7.9 vs. 10%; F: 7.0 vs. 3.4%, M: 29 vs. 20%). Polishing particularly improved low‐quality assemblies by reducing errors and duplications, but did not significantly increase contig length. The need for polishing thus depends on the obtained assembly quality and is particularly attractive when working under low coverage scenarios (<20×). In addition, 25× compared to 16× PacBio data delivered long‐read assemblies of substantially fewer and larger contigs (4209 vs. 9597 and 6.0 vs. 1.2 Mbp), more single‐copy BUSCO genes found (36% vs. 27%), and fewer duplicated (63% vs. 73%), fragmented (0.34 vs. 1.0%), and missing genes (0.20% vs. 3.2%), but showed similar genome completeness across long‐read strategies (Table 3, Tables S4 vs. S5, Figure 6 vs. Figure S10). Coverage much below 25× is therefore not recommended for either strategy.

Finally, the 25× PacBio‐based Hifiasm assembly polished twice by PacBio and once more by Illumina reads delivered the best results (100% expected genome completeness, 1319 contigs, 20 Mbp N50 and 99% complete BUSCO genes). This represents the best assembly strategy for the large and complex *R. cassubicifolius* nuclear genome with low to moderate sequencing coverage, and agrees with findings in assemblies of other non‐model plants (e.g., Dmitriev et al., 2021).

## CONCLUSIONS

In this study, we assembled and annotated the plastome, mitogenome, and nuclear genome sequences of *R. cassubicifolius*, a diploid sexual progenitor species of the *R. auricomus* complex. We generated a plastid and the first mitogenome for *Ranunculus* based on Illumina and Illumina + PacBio data, respectively. An updated plastome phylogeny and the first mitogenome phylogeny of Ranunculaceae indicated frequent gene losses (e.g., *infA*, *ycf15*, and *rps* genes), potentially in the context of environmental adaptation and reproductive features. To assemble the large and complex nuclear genome, we tested 18 different strategies using short‐, hybrid‐, and long‐read strategies, followed by gap filling and/or polishing with various state‐of‐the‐art bioinformatic tools. The best result in terms of contiguity and completeness was achieved by Hifiasm using PacBio data, polished twice with filtered PacBio reads (Racon, polishCLR) and once with filtered Illumina reads (POLCA). This is the first high‐quality, chromosome‐scale, and annotated nuclear genome sequence for *Ranunculus* and the second, but largest for Ranunculaceae on NCBI. The *R. cassubicifolius* genome with 2.69 Gb size, containing 35 482 annotated genes and 86% repetitive elements, represents a typical medium‐sized plant genome. In comparison with other Ranunculales, we detected two ancient genome duplication events.

The obtained results and in particular the new nuclear genome sequence reference are useful for (i) orthologous gene/locus assembly and allele phasing of reduced‐representation sequencing (e.g., RAD‐Seq, GBS, or target enrichment), transcriptome (RNA‐seq), or whole genome (re)sequencing (e.g., WGS or WGR) data, (ii) as a basis for understanding cyto‐nuclear compatibility for both plastid‐nuclear and mitochondrial‐nuclear interactions; (iii) for the analysis of the more complex allo‐ and autopolyploid nuclear genomes within the genus *Ranunculus*, as well as (iv) for improving functional analyses (e.g., gene expression studies) related to apomixis or environmental adaptation.

## METHODS

### Plant material

**Figure 7:**
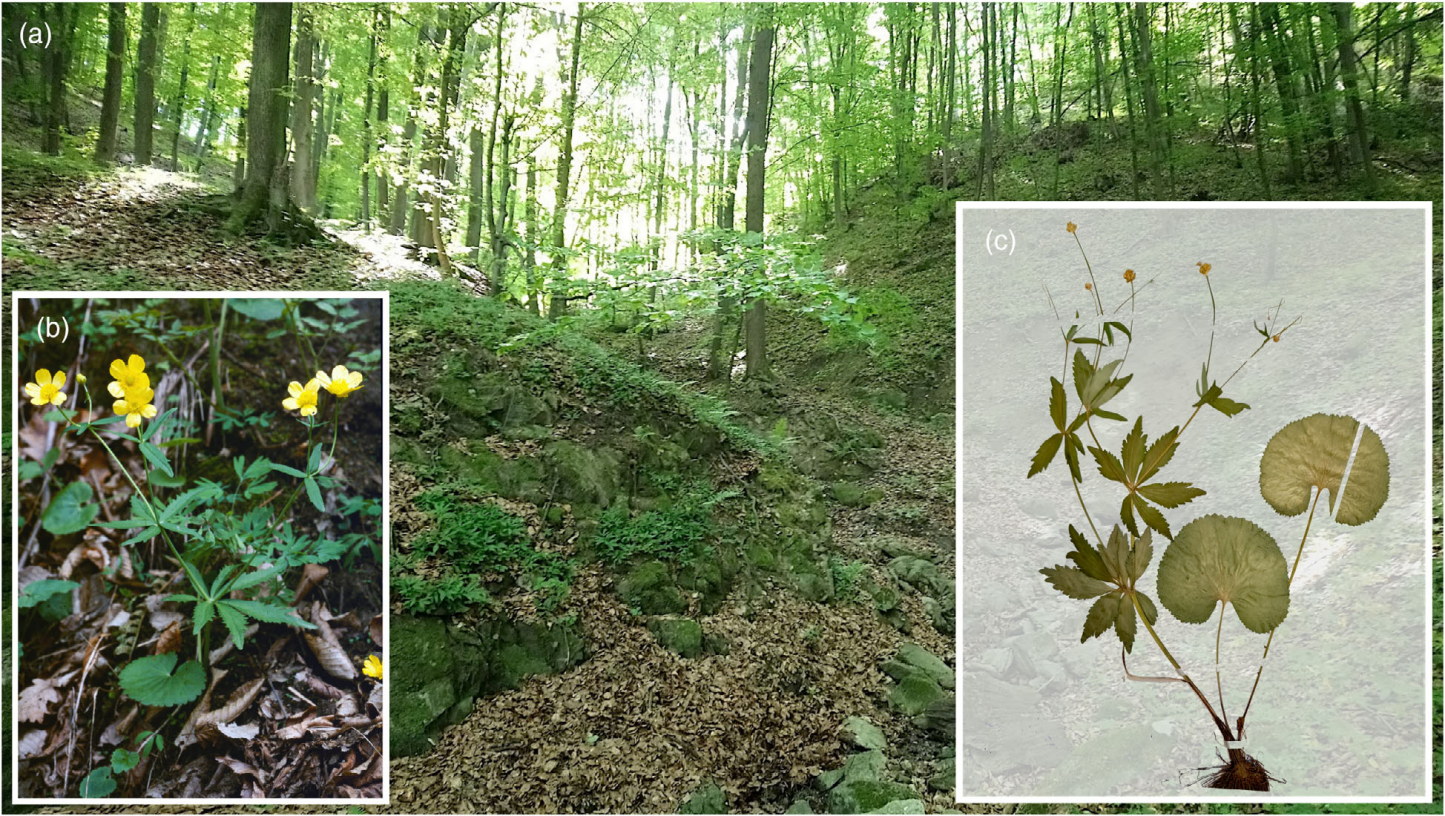
Characteristics of the plant species *Ranunculus cassubicifolius* (Ranunculaceae) examined in the present study. (a) Habitat of *R. cassubicifolius* (previously listed as “*R. carpaticola*”) population LH040 in a forest near Revúca, Slovakia (May 4, 2018, L. Hodač & K. Spitzer; details in Karbstein, Tomasello, et al., 2020). (b) Habit of the species with undissected basal leaves and typical flowers (population EH8483). (c) The scan of a representative herbarium specimen from the population LH040. Field images: Elvira Hörandl, Ladislav Hodač.

In the present study, analyses were performed on diploid sexual individuals (2n = 16) of *R. cassubicifolius* (*R. auricomus* species complex, Figure 7; taxonomic treatment follows Karbstein, Tomasello, et al., 2020). Chromosome numbers, ploidy levels, and reproduction modes are published in Hörandl & Greilhuber (2002) under “*R. carpaticola*”, in Karbstein, Tomasello, et al. (2020), and Karbstein et al. (2021), and sequenced individuals are listed in Table 4. We had to harvest different individuals from the same population because plants did not sprout again after the intense collection of leaves needed for genomic DNA. All plants were cultivated at the Old Botanical Garden of the University of Göttingen.

**Table 4:** Plant materials used in the present study

Sample ID (population/individual)	Location	Illumina	ONT	PacBio	Hi‐C	NCBI BioSample	References
EH8483/10	48°41′39″ N, 20°07′42″ E	x				SAMN27582367	Hörandl & Greilhuber, 2002
LH040/02	48°40′38″ N, 20°06′24″ E		x			SAMN27753242	Karbstein, Tomasello, et al., 2020
LH040/06	48°40′38″ N, 20°06′24″ E			x	x	SAMN44350262	Karbstein, Tomasello, et al., 2020
LH040/05	48°40′38″ N, 20°06′24″ E				x	SAMN44350262	Karbstein, Tomasello, et al., 2020

All plants were sampled around the city of Revúca, Slovakia.

*Ranunculus cassubicifolius* represents a distinct, outcrossing lineage and only shows very low intraspecific differentiation and heterozygosity in nuclear markers (Paun et al., 2006; Karbstein, Tomasello, et al., 2020; Karbstein et al., 2021, 2022; e.g., <0.1% for LH040/04 in Tomasello et al., 2020). Therefore, sequencing different individuals from the same populations is not expected to introduce bias into the genome polishing and scaffolding processes.

### Illumina DNA extraction and sequencing

For Illumina short‐read sequencing, fresh leaves were harvested in 2019, and high‐molecular‐weight genomic DNA was extracted immediately after harvest from ca. 200 mg of material from a single *R. cassubicifolius* individual (EH8483/10) using liquid nitrogen and the DNeasy PowerPlant Pro Kit (Qiagen, Hilden, Germany) according to the manufacturer's instructions. DNA size and concentration were measured with the Qubit Fluorometer 2.0 and the Qubit dsDNA HS Assay Kit (ThermoFisher Scientific, Waltham, MA, USA), and the Genomic DNA ScreenTape device (Agilent Technologies, Santa Clara, CA, USA). The extraction yielded 16 ng/μl of DNA with a median fragment length of 19 kbp. Library preparation followed the 10× Chromium Controller Genomics Kit (10× Genomics, Pleasanton, CA, USA) according to the manufacturer's instructions. Library quality was assessed by a qPCR based on the NEBNext Library Quant Kit (New England Biolabs, Ipswich, MA, USA). Sequencing with standard Illumina primers was performed on a NextSeq 500/550 instrument (Illumina, San Diego, CA, USA) with 2 × 150 bp reads.

We converted the output into FASTQ files using Supernova v2.1.1 (10× Genomics, San Francisco, CA, USA; Marks et al., 2017; Weisenfeld et al., 2018) and bcl2fastq v2.20 (Illumina). We conducted polyX‐tail and adapter trimming, and quality filtering of raw reads using Atria v3.1.2 (Chuan et al., 2021) with default settings, and BBTools/BBMap v39.01 (“repair.sh”; Bushnell, 2022) to reorder filtered paired‐end reads. The quality of the sequenced reads was checked by FastQC v0.11.4 (Andrews, 2010; reports on FigShare [01 = Item 01 in the FigShare repository], https://doi.org/10.6084/m9.figshare.20488908 incl. a README file).

### Nanopore DNA extraction and sequencing

For ONT long‐read sequencing, we harvested fresh, young leaf material in the early morning in 2021, after 12‐h dark adaptation at room temperature. We used fresh leaf material (ca. 75 mg) from a single *R. cassubicifolius* individual (LH040/02) and the Qiagen protocol for the isolation of gDNA from plants and fungi using Genomic Tip 20/G. Library preparation was conducted using the ONT Ligation Sequencing Kit SQK‐LSK110 optimized for high throughput and long reads (ONT, Oxford, UK). We adjusted the DNA concentration to 1000 ng in 47 μl (ca. 21.5 ng/μl). We used a MinION 101B device, the software MinKNOW v21.11.7, 21.11.9 and 22.03.5, and six R9.4.1 flow cells following the manufacturer's instructions for priming and loading the prepared libraries. After 1–2 days of run time, the old library was usually exhausted and replaced with a second library using the supplied flow cell wash kit. Sequencing was run for 3–5 days per flow cell until the majority of pores were inactive (>99%). The DNA extraction and quality control, and library preparation are described in Text S1 and Figure S1 in detail. Reports per flow cell can be accessed via FigShare [Item 01].

Basecalling was performed with the ONT software GUPPY v6.0.1 specifying the configuration file “dna_r9.4.1_450bps_fast.cfg” for fast basecalling. In preruns, the difference in mean base accuracy between the settings “fast” and the substantially slower “high accuracy” was quite small, that is, 97 versus 98%. Next, NanoFilt v2.2.0 (De Coster et al., 2018) was applied to remove the first 50 bp of the reads, which were typically of low quality.

### PacBio DNA extraction and sequencing

For PacBio long‐read sequencing, high‐molecular‐weight gDNA was isolated from fresh leaf material of a single *R. cassubicifolius* individual (LH040/06) with the NucleoBond HMW DNA kit (Macherey Nagel, Germany), following the manufacturer's instructions. The integrity of the DNA was determined by the Femto Pulse System (Agilent Technologies, Santa Clara, CA, USA). The DNA concentration was measured with the Qubit dsDNA High Sensitivity assay kit (Thermo Fisher Scientific, Waltham, MA, USA). For the construction of a HiFi library, 7.5 μg HMW DNA was fragmented (speed 29) using the Megaruptor 3 device (Diagenode SA, Seraing, Belgium). In total, two HiFi libraries were constructed, pooled, and sequenced on a single Revio 25 M SMRT cell. Libraries were generated according to the “Procedure & Checklist—Preparing whole genome and metagenome libraries using SMRTbell® prep kit 3.0” manual (102–166‐600; Pacific Biosciences, Menlo Park, CA, USA). For size fractionation, a SageELF (Sage Science, Beverly, MA, USA) device was employed. The fragment size of the final library (16.2 kb) was measured using the Femto Pulse System (Agilent Technologies). Polymerase‐bound SMRTbell complexes were formed according to standard manufacturer's protocols. Sequencing (HiFi CCS) was conducted using the Pacific Biosciences Revio instrument (24 h movie time, loading concentration 230 pM, 2 h pre‐extension time, diffusion loading, mean insert length according to SMRT link raw data report: 18.0 kbp) following standard manufacturer's protocols. All steps were done at IPK Gatersleben.

HiFiAdapterFilt v2.0.0 (Sim et al., 2022) was run with default settings to convert the HiFi reads from BAM to FASTQ format, and to remove reads with remnant adapter sequences.

### Chromosome conformation capture (Hi‐C) DNA extraction and sequencing

A Hi‐C sequencing library was generated from fresh leaf material of two *R. cassubicifolius* individuals (LH040/05 + 06) with the *DpnII* enzyme, following Padmarasu et al. (2019). The library was sequenced (paired‐end: 2 × 111 cycles, two indexing reads: 8 cycles, v1.5 chemistry) on a NovaSeq6000 instrument (Illumina, San Diego, USA) according to standard manufacturer's protocols. All steps were performed at IPK Gatersleben.

### Genomic data analysis

#### The plastid genome

The plastome sequence was assembled using Illumina reads and the software GetOrganelle v1.7.5.3 (Jin et al., 2020) as recommended by Freudenthal et al. (2020), specifying the “embplant_pt” database, k‐mer size range between 45 and 121, and as seed the *Ranunculus repens* plastome (154 247 bp, NC_036976; Dann et al., 2017). The average sequencing depth (the average number of reads per position) was 560×. The plastome was annotated using CPGAVAS2 (Shi et al., 2019) with default settings and 2544 reference plastomes, and visualized with CPGView (Liu et al., 2023). We checked the annotation results with Geneious R11 2023.1.2 (Kearse et al., 2012). The final annotated plastome sequence was deposited in GenBank (NC_077490). We applied MAFFT v7.490 to align and investigate differences between *R. cassubicifolius* and other *Ranunculus* plastome sequences.

#### The mitochondrial genome

We used GetOrganelle with the same settings as described above, except that we specified “embplant_mt” and the closest available mitogenome (*Hepatica maxima*, NC_053368.1; Park & Park, 2020) as reference. The average sequencing depth was estimated at 92×. To improve the assembly, we first mapped ONT reads against the numerous contigs derived from GetOrganelle and extracted the mapped reads using SAMtools v1.9 (“faidx” and “view”; Danecek et al., 2021), Minimap2 v2.27 (“‐ax map‐ont”; Li, 2018) and BEDTools v2.29.1 (“bamtofastq”; Quinlan & Hall, 2010). We applied MaSuRCA v4.0.9 (Zimin & Salzberg, 2022) to perform a hybrid assembly based on extracted ONT reads and “embplant_mt” hitting Illumina reads derived from GetOrganelle.

Then, we retained an ONT‐based mitogenome sequence in 18 contigs and a total length of 351 681 bp. We aligned contigs in Geneious to merge overlapping regions, which resulted in 4 contigs concatenated to a single sequence (orientation followed *H. maxima*), onto which Illumina and ONT reads were mapped using minimap2 (“‐ax sr” and “‐ax map‐ont”, respectively; mappings and contig borders on FigShare [Item 03]). We inferred an average Illumina read depth of 62× (2.87% sites without read depth), and an ONT read depth of 3640× (9.88% sites without read depth), respectively. The assembly was annotated using the MitoHiFi pipeline v3.2.1 (Uliano‐Silva et al., 2023) including gene annotation with *H. maxima* as the reference, and visualized with PMGmap (Zhang, Chen, et al., 2024). We ran RepeatModeler2 v2.0.5 to additionally check for dispersed repeats. In a second assembly strategy, we performed the hybrid assembly with HiFi reads as described above. This resulted in 40 raw contigs with 1 913 766 bp summarized into nine contigs that were aimed to be arranged like the ones in the ONT hybrid mitogenome. We inferred an average Illumina read depth of 269× (1.96% sites without read depth) and HiFi read depth of 3324× (0.12% sites without read depth).

Gap regions were usually spanned by Illumina‐ and/or ONT‐/PacBio reads. The absence of reads at certain sites is likely due to highly divergent regions of the mitogenome. Several populations of mitogenome sequences usually exist within a cell (Møller et al., 2021; Sloan, 2013). The assembled “consensus” mitogenome sequence is a mixture of these populations, potentially resulting in regions that cannot be spanned by raw reads. When comparing sites covered by both read types, there are no sites without reads for the PacBio‐based hybrid assembly. For the ONT‐based hybrid assembly, there are only sites without relatively long ONT reads at the start of the linearized assembly because the assembler did not take circularity into account. Sites without Illumina reads were mainly found in repetitive regions. We performed progressiveMauve (2015‐02‐25; Darling et al., 2010) to investigate mitogenomic rearrangements between the PacBio and ONT assemblies, and between *R. cassubicifolius* and other Ranunculaceae mitogenome sequences.

Geneious was used to check the annotation results. The resulting GenBank file was converted into a feature table for GenBank submission using the GB2sequin tool (Lehwark & Greiner, 2019) within the MPI‐MP CHLOROBOX webserver. Finally, we selected the PacBio‐based mitogenome because of a higher depth of mapped short‐ and long‐reads, and fewer sites without read depth. The annotated mitogenome was deposited in GenBank (PP657143).

#### Phylogenomics and organellar gene evolution within Ranunculacaeae

To study gene evolution within Ranunculaceae, we downloaded all available organellar genome sequences from NCBI using the custom Python script “findGenome.py” (https://github.com/KK260/NCBI‐Genome‐Tools/). Applying the specifications “complete genome” AND “Ranunculaceae” AND “chloroplast”/ “mitochondrion,” we found 1161 plastome and 16 mitogenome sequences that were filtered by duplicate removal and a maximum of one individual per species (favoring the latest RefSeq “NC” records). Taxon names were checked for synonyms using the Catalogue of Life on GBIF. This yielded 306 plastomes including six outgroup taxa of Berberidaceae and Papaveraceae (292 taxa), and 10 mitogenomes including one outgroup taxon of Papaveraceae (none available for Berberidaceae, 10 taxa; see NCBI accessions in Table S1).

Plastome sequences were aligned using MAFFT with mode “FFT‐NS‐2” (Katoh & Standley, 2013), following the deletion of sites with less than 50, 70 and 90% of available samples using Goalign v0.3.7 (Lemoine & Gascuel, 2021). We then ran ModelTest‐NG v0.1.7 (Darriba et al., 2020), which determined “TVM+I+G4” (unfiltered), “TIM1+I+G4” (min50), and “GTR+I+G4” (min70, min90) as best‐fit evolutionary models. Maximum likelihood (MaxLik) trees were inferred with RAxML‐NG v1.2.1 (Kozlov et al., 2019), specifying the sequence model and 100 bootstraps. Quartet Sampling (QS) v1.3.1 (Pease et al., 2018) scores were calculated with default settings. The final phylogenetic tree was selected according to the highest mean TBE support and optimal QS scores (QC = 1, QD = 0, QI = 1) using the R package phytools v2.0–3 (Revell, 2012, 2024). We chose “min90” (126 kbp) with the lowest number of gaps excluding poorly assembled non‐coding DNA regions, and the highest TBE and QS values in the phylogeny as the optimal alignment (Figures S2–S4 and Text S2, and alignments on FigShare [Item 02]). Branches of all tribes except *Ranunculeae* were collapsed with FigTree v1.4.4 (Rambaut, 2014), and gene gain and loss events were mapped onto the tree.

The mitogenome phylogeny was built on a coalescent‐based method because of substantial genomic rearrangements observed among species but also within *R. cassubicifolius* (Figure S5a,b, see Figure S6 for the MaxLik phylogeny). Using the custom Python script “assembleGenes.py,” we ran Astral v5.7.8 with 100 bootstraps based on collapsed gene trees of low support previously inferred by RAxML_NG, and mapped gene features onto the resulting tree. Branch support is indicated by local posterior probabilities (PP) and quartet support values.

In Ranunculaceae, gene loss per group derived from the feature table was manually verified by mapping (max. 5% sequence divergence) extracted genes from closely related species to the sequence of interest in Geneious. In *R. cassubicifolius*, we aimed to find lost organellar genes by mapping the genes from closely‐related species to the nuclear genome sequence.

#### The nuclear genome

We applied different assembly strategies based on an estimated haploid genome size of 3.2 Gbp: (i) using Illumina reads, we ran Supernova and SPAdes v3.13.2 (Prjibelski et al., 2020); (ii) using Illumina and ONT or PacBio/HiFi reads, we ran SPAdes (untrusted Supernova contigs for gap filling), MaSuRCA v4.1.0 (Zimin et al., 2017), MuCHSALSA v0.02 (Gatter et al., 2021) and Wengan (M) v0.2 (Di Genova et al., 2021); (iii a) using ONT reads, we ran Canu v2.1.1 (Koren et al., 2017) and Flye v2.9.1 (Kolmogorov et al., 2019), followed by the best strategy of Dmitriev et al. (2021) of two times polishing with filtered ONT reads using Racon v1.4.3 (Vaser et al., 2017) and Medaka v0.7.1 (https://github.com/nanoporetech/medaka), and polishing with Illumina reads using POLCA (Zimin & Salzberg, 2020); (iii b) using HiFi reads, we ran Canu, Flye, and Hifiasm v0.19.8 (Cheng et al., 2021), followed by the previous polishing strategy except for the use of polishCLR v1.1.0 (Chang et al., 2023) instead of Medaka. To generate comparable assembly conditions for ONT and PacBio data, we downsampled PacBio reads to 16× coverage using reformat.sh from BBTools and reran hybrid‐ and long‐read analyses.

Statistics for genome assemblies were calculated using QUAST v5.0.1 (Gurevich et al., 2013). Assembly completeness in terms of expected single‐copy genes was estimated by “Benchmarking Universal Single‐Copy Orthologs” (BUSCO v5.0.0; Waterhouse et al., 2018; Manni et al., 2021) with the “embryophyta_odb10” 2020‐09‐10 dataset containing 1614 core genes. The assembly with the highest completeness, largest contig size, and best BUSCO scores was selected for scaffolding with Hi‐C data and genome annotation. Filtered Hi‐C reads were mapped to the HiFi polished contigs using Juicer v1.6 (Durand et al., 2016) with default settings. We applied the 3D‐DNA pipeline v170123 (Dudchenko et al., 2017) to order and orient the clustered contigs with sizes >15 000 bp. Misassemblies were identified and fixed manually based on neighboring interactions using the assembling mode in the Juicebox software (Hi‐C contact map in Figure S7).

The genome assembly was then subjected to the identification of tandem repeats (TRs) and transposable elements (TEs), and protein‐coding gene annotation (details in Text S3). Repetitive elements were identified using Tandem Repeats Finder v4.10.1 (Benson, 1994) and EDTA v2.2.1. The EDTA pipeline integrates the results of LTR‐FINDER (Ou & Jiang, 2019; Xu & Wang, 2007), LTR_retriever (Ou & Jiang, 2018), Generic Repeat Finder (Shi & Liang, 2019), TIR‐Learner (Su et al., 2019; Xiong et al., 2014), HelitronScanner (Xiong et al., 2014; Zhang et al., 2022), and TEsorter to achieve accurate and efficient TE identification and classification. MMseqs2 was used to identify inter‐ and intra‐chromosomal syntenic regions. We used a multi‐species mapping approach by including RNA reads from multiple *Ranunculus* species (37 individuals, Table S2), retrieved using prefetch and fasterq‐dump from NCBI SRA toolkit v3.1.0. RNA data was mapped onto the genome using HISAT2 v2.2.1 (Kim et al., 2019). Structural annotation of protein‐coding genes was done with a combination of three approaches:

- First, homology‐based gene prediction using GeMoMa v1.9 (Keilwagen et al., 2016, 2018), based on annotations from 14 Ranunculales species (Table S3). The coding genes from these 14 species were aligned to the *R. cassubicifolius* genome sequence using MMseqs2 v15‐6f452 (Steinegger & Söding, 2017) following the default settings of GeMoMa. To refine intron boundaries, RNA‐seq data from five recently published diploid *R. auricomus* transcriptomes (Paetzold et al., 2022) were incorporated. The resulting gene annotation sets were merged and filtered using the GeMoMa Annotation Filter (GAF).
- Second, we applied the BRAKER3 pipeline (Gabriel et al., 2024), which relies on ab initio gene prediction with AUGUSTUS v3.5.0 (Stanke et al., 2006, 2008) and GeneMark‐ETP v1.02 (Brůna et al., 2024), corrected by extrinsic evidence from RNA‐seq and protein homology. For protein evidence, we used the “Viridiplantae” library (Kuznetsov et al., 2023) and annotated NCBI genomes of Ranunculaceae species (Table S3). The resulting BRAKER3 annotation was filtered using GeMoMa Annotation Filter (GAF).
- Third, we employed Funannotate v1.8.15 (Palmer & Stajich, 2022), initially developed for fungi but now capable of handling larger genomes. Repetitive contigs were cleaned from the genome using minimap2 v2.26, and simple repeats were masked using tantan (“‐s arabidopsis”; Frith, 2011). Approximately 8.0% (217 Mbp) of the genome sequence was masked. The filtered genome and transcriptome sequences were used to train the annotation process by a genome‐guided transcriptome assembly based on Trimmomatic v0.39 (Bolger et al., 2014), Trinity v2.8.5 (Grabherr et al., 2011), HISAT2, kallisto v0.46.1 (Bray et al., 2016), and PASA v2.5.3 (Haas et al., 2008) to filter, normalize, and cluster (genome‐guided) transcriptomic data. The sorted alignments, transcripts, UniProt protein library, and transcriptome annotations were the input for the gene prediction process performed by AUGUSTUS v3.5.0, GlimmerHMM (Majoros et al., 2004), and SNAP (Korf, 2004). Results were summarized with EVidenceModeler v1.1.1 (Haas et al., 2008).

The annotations resulting from GeMoMa, BRAKER3, and Funannotate were compared based on BUSCO completeness scores. Based on these comparisons, the GeMoMa and BRAKER3 gene predictions were merged using GAF. Gene models in disagreement with RNA evidence data were fixed manually. The annotation quality was evaluated using BUSCO v5.6.1 with the embryophyta_odb10 database. Functional annotation was performed with InterProScan5 (Jones et al., 2014) and eggNOG‐mapper (Cantalapiedra et al., 2021) using the Funannotate “annotate” function. Non‐coding RNA features, including rRNAs, tRNAs, and short ncRNAs, were identified using Infernal v1.1.5 (Nawrocki & Eddy, 2013), which scanned covariance models from the Rfam database v15 (Ontiveros‐Palacios et al., 2024). Additionally, tRNAscan‐SE v2.0.12 (Chan et al., 2021) and barrnap v0.9 (https://github.com/tseemann/barrnap) were used to enhance the detection of tRNAs and rRNAs, respectively. The nuclear genome sequences and annotated protein‐coding genes are deposited in NCBI under GCA_049309505.

We scanned the genome for complex repeat structures and centromeric regions based on sequence similarity using ModDotPlot v0.9.0 (Sweeten et al., 2024). To infer ancient whole genome duplications in our species of interest and in Ranunculales (Ranunculaceae: *Aquilegia caerulea*, *Coptis chinensis*, and *Thalictrum thalictroides*; Menispermaceae: *Stephania cephalantha*; Papaveraceae: *Papaver somniferum*; references in Table S3), we conducted analyses with the wgd pipeline v2.0.38 based on protein‐coding genes of high‐quality genomes. We followed default recipes for inference of orthogroups, synonymous substitutions per site of duplicated genes (K_S_ distribution), intra‐ and interspecies gene synteny based on orthogroups, and absolute peptide‐based dating of WGDs using a Bayesian MCMC tree approach (Chen et al., 2024; Chen & Zwaenepoel, 2023). Only one transcript per gene and species was used to avoid false positive duplication bumps caused by alternative splicing. The phylogenetic tree used for dating including secondary calibration points was calculated in Orthofinder v2.5.5 (Emms & Kelly, 2019). We received the following adjusted divergence times as secondary calibration points from timetree.org (Kumar et al., 2022): *P*. *somniferum* and remaining Menispermaceae and Ranunculaceae: 125 mya (102.9–117.2), Menispermaceae and Ranunculaceae: 92 mya (75.9–103), *C*. *chinensis* and remaining Ranunculaceae: 64 mya (25.7–79.6), *T. thalictroides* / *A. coerulea* and *R. cassubicifolius*: 60 mya (41.7–123.6; removing calibration points >100 mya led to similiar dating results, see item 04 on FigShare), and *T. thalictroides* and *A. coerulea*: 21 mya (9.6–36). To classify segmental duplications (SD), and small‐scale duplications (SSD) as tandem (TD), proximal (PD), or dispersed duplications (DD), the pipeline doubletrouble v.1.6.0 was run with default settings (Almeida‐Silva and Van de Peer, 2025).

Data analyses were mainly performed on the HPC cluster of the GWDG (Göttingen, Germany), except for the Wengan genome assemblies and genome annotation, which were run on a local HPC cluster of the TU Ilmenau and the de.NBI cloud, respectively.

## AUTHOR CONTRIBUTIONS

KK and EH designed research; EH collected plant materials; KK, AH, MP, ST, JPB, and BHB performed lab work; KK, NC, XT, BP, and ST analyzed data; KK and EH wrote the paper, with contributions from NC, II, BP, JdV, XT, AH, NS, ST, AP, NW, BHB, JPB, CP, and MP.

## Supporting information


**Text S1.** (a, b) Extraction of genomic DNA (gDNA) and (b) library preparation for Oxford Nanopore Technology (ONT) sequencing performed at the University of Göttingen.
**Text S2.** Determining the optimal DNA sequence alignment for phylogenetic analyses.
**Text S3.** Identification of tandem repeats (TRs) and transposable elements (TEs), and protein‐coding genes.
**Text S4.** Detailed results of plastome‐based phylogenies in Ranunculaceae.
**Text S5.** Impact of using frozen libraries for ONT DNA sequencing.
**Figure S1.** Gel electrophoresis of gDNA extractions from 17th December 2021 using 1 kb DNA Ladder (New England Biolabs, Ipswich, MA, USA; 500 bp–10 kb) as size standard.
**Figure S2.** (a–d) Maximum‐likelihood phylogeny based on min0 (no filtering), min50, min70, and min90 alignments of 306 plastomes (taxa) of the plant family Ranunculaceae.
**Figure S3.** (a, b) Maximum‐likelihood phylogeny based on 306 plastomes (292 taxa) of the plant family Ranunculaceae.
**Figure S4.** Maximum‐likelihood phylogeny based on 306 plastome sequences (292 taxa) and the min90 alignment of the plant family Ranunculaceae.
**Figure S5.** Whole genome alignment analysis of (a) all available mitogenome sequences in Ranunculaceae, and (b) of the assembled Illumina‐ONT and ‐PacBio genome sequences of *Ranunculus cassubicifolius* (LH040).
**Figure S6.** Concatenation‐based phylogeny of 10 mitogenome sequences and 42 genes of Ranunculaceae (see Figure 3b for the coalescent‐based phylogeny).
**Figure S7.** Hi‐C contact map.
**Figure S8.** (a–h) ModDotPlots of pseudochromosomes 1–8 of the final PacBio genome assembly (Table 1, ‘Nuclear Genome’).
**Figure S9.** Detection of ancient whole genome duplication (WGD) events in *Ranunculus cassubicifolius*.
**Figure S10.** BUSCO assessments (PacBio 25×) for different genome assembly strategies of the diploid sexual species *Ranunculus cassubicifolius*.
**Table S1.** Selected (a) plastome and (b) mitogenome sequences from NCBI.
**Table S2.** RNA‐seq data of 37 Ranunculaceae individuals from SRA/NCBI used for *Ranunculus cassubicifolius* genome annotation. See Methods for annotation details.
**Table S3.** Genome annotation data of 14 Ranunculales species from NCBI used for genome annotation of *Ranunculus cassubicifolius*.
**Table S4.** QUAST statistics (PacBio 16×) for different genome assembly strategies of *Ranunculus cassubicifolius*. Supernova, and partially SPAdes, MaSuRCA, and Wengan used Illumina short‐reads (EH8483/10).
**Table S5.** QUAST statistics (PacBio 25×) for different genome assembly strategies of *Ranunculus cassubicifolius*. Supernova, and partially SPAdes, MaSuRCA, and Wengan used Illumina short‐reads (EH8483/10).
**Table S6.** (a‐c) BUSCO statistics of nuclear genome sequence assemblies.

## Data Availability

The basic data supporting the findings are available within the manuscript and Supporting Information. We deposited DNA alignments, intermediate results, and tool reports including a Readme file on FigShare (https://doi.org/10.6084/m9.figshare.20488908), and the used Python scripts “findGenome.py” and “assembleGenes.py” on GitHub at https://github.com/KK260/NCBI‐Genome‐Tools. The non‐coding gene and transposable elements annotations are available on GitHub at https://github.com/NancyChoudhary28/Ranunculus‐genomics. Raw sequencing data and the assembled sequences are deposited at the National Center for Biotechnology Information (NCBI) database under the BioProject accession numbers: PRJNA826743 for Illumina raw reads (https://www.ncbi.nlm.nih.gov/bioproject?LinkName=biosample_bioproject&from_uid=27582367), PRJNA831351 for filtered ONT, PacBio, and Hi‐C reads, filtered mitochondrial ONT and PacBio reads, and filtered mitochondrial Illumina reads (https://www.ncbi.nlm.nih.gov/bioproject/PRJNA831351/, https://www.ncbi.nlm.nih.gov/Traces/index.html?view=run_browser&acc=SRR31094232&display=data‐access); GCA _049309505 for the assembled and annotated genome (https://www.ncbi.nlm.nih.gov/datasets/genome/GCA_049309505.1/); NC_077490 for the assembled and annotated plastome (https://www.ncbi.nlm.nih.gov/search/all/?term=NC_077490), PP657143 for the assembled and annotated mitogenome (https://www.ncbi.nlm.nih.gov/search/all/?term=PP657143).
