# Supplementary material for: Assembling genomes of non‐model plants: A case study with evolutionary insights from Ranunculus (Ranunculaceae)
Source: Plant J. 2025 Sep 19;123(6):e70390. doi: 10.1111/tpj.70390 (PMC12448783; doi:10.1111/tpj.70390)
Supplement: Supplementary file 1 — Text S1. (a, b) Extraction of genomic DNA (gDNA) and (b) library preparation for Oxford Nanopore Technology (ONT) sequencing performed at the University of Göttingen. Text S2. Determining the optimal DNA sequence alignment for phylogenetic analyses. Text S3. Identification of tandem repeats (TRs) and transposable elements (TEs), and protein‐coding genes. Text S4. Detailed results of plastome‐based phylogenies in Ranunculaceae. Text S5. Impact of using frozen libraries for ONT DNA sequencing. Figure S1. Gel electrophoresis of gDNA extractions from 17th December 2021 using 1 kb DNA Ladder (New England Biolabs, Ipswich, MA, USA; 500 bp–10 kb) as size standard. Figure S2. (a–d) Maximum‐likelihood phylogeny based on min0 (no filtering), min50, min70, and min90 alignments of 306 plastomes (taxa) of the plant family Ranunculaceae. Figure S3. (a, b) Maximum‐likelihood phylogeny based on 306 plastomes (292 taxa) of the plant family Ranunculaceae. Figure S4. Maximum‐likelihood phylogeny based on 306 plastome sequences (292 taxa) and the min90 alignment of the plant family Ranunculaceae. Figure S5. Whole genome alignment analysis of (a) all available mitogenome sequences in Ranunculaceae, and (b) of the assembled Illumina‐ONT and ‐PacBio genome sequences of Ranunculus cassubicifolius (LH040). Figure S6. Concatenation‐based phylogeny of 10 mitogenome sequences and 42 genes of Ranunculaceae (see Figure 3b for the coalescent‐based phylogeny). Figure S7. Hi‐C contact map. Figure S8. (a–h) ModDotPlots of pseudochromosomes 1–8 of the final PacBio genome assembly (Table 1, ‘Nuclear Genome’). Figure S9. Detection of ancient whole genome duplication (WGD) events in Ranunculus cassubicifolius. Figure S10. BUSCO assessments (PacBio 25×) for different genome assembly strategies of the diploid sexual species Ranunculus cassubicifolius. Table S1. Selected (a) plastome and (b) mitogenome sequences from NCBI. Table S2. RNA‐seq data of 37 Ranunculaceae individuals from SRA/NCBI used for Ranunc [file TPJ-123-0-s001.zip › tpj70390-sup-0001-Supinfo.docx]

**Assembling genomes of non-model plants: A case study with evolutionary insights from *Ranunculus* (Ranunculaceae)**

**Karbstein, Kevin^1,2,3*^, Choudhary, Nancy^4^, Xie, Ting^5^, Tomasello, Salvatore^1^, Wagner, Natascha D.^1^, Barke, Birthe H.^1^, Paetzold, Claudia^6^, Bradican, John P.^1^, Preick, Michaela^7^, Himmelbach, Axel^8^, Stein, Nils^8,9^, Papantonis, Argyris^5^, Irisarri, Iker^10^, de Vries, Jan^11,12,13^, Pucker, Boas^4^, & Hörandl, Elvira^1*^**

**^1^** *University of Göttingen, Albrecht-von-Haller Institute for Plant Sciences, Department of Systematics, Biodiversity and Evolution of Plants (with Herbarium), Göttingen, Germany*

**^2^** *Max Planck Institute for Biogeochemistry, Department of Biogeochemical Integration, Jena, Germany*

**^3^** *Data‑Intensive Systems and Visualization Group (dAI.SY), Technical University Ilmenau,* *Ilmenau, Germany*

**^4^** *Institute for Cellular & Molecular Botany (IZMB), University of Bonn, Bonn, Germany*

**^5^** *University Medical Center Göttingen, Institute of Pathology, Göttingen, Germany*

**^6^** *Senckenberg Naturhistorische Sammlungen, Dresden, Germany*

**^7^** *University of Potsdam, Institute for Biochemistry and Biology, Potsdam, Germany*

**^8^** *Leibniz Institute of Plant Genetics and Crop Plant Research (IPK), Seeland, Germany*

**^9^** *Center of integrated Breeding Research (CiBreed), Department of Crop Sciences, Göttingen, Germany*

**^10^** *Museo Nacional de Ciencias Naturales (MNCN-CSIC), Department of Biodiversity and Evolutionary Biology, Madrid, Spain*

**^11^** *University of Göttingen, Institute for Microbiology and Genetics, Department of Applied Bioinformatics, Göttingen, Germany*

**^12^** *University of Göttingen, Campus Institute Data Science (CIDAS), Göttingen, Germany*

**^13^** *University of Göttingen, Göttingen Center for Molecular Biosciences (GZMB), Department of Applied Bioinformatics, Göttingen, Germany*

**ORCID**

Kevin Karbstein 0000-0003-1424-6557

Nancy Choudhary 0000-0002-2562-7905

Ting Xie 0000-0002-6648-7697

Salvatore Tomasello 0000-0001-5201-1156

Natascha D. Wagner 0000-0001-6623-7623

Birthe H. Barke 0000-0001-5379-6055

Claudia Paetzold 0000-0002-4128-6645

John P. Bradican 0000-0001-5650-6172

Axel Himmelbach 0000-0001-7338-0946

Nils Stein 0000-0003-3011-8731

Argyris Papantonis 0000-0001-7551-1073

Iker Irisarri 0000-0002-3628-1137

Jan de Vries 0000-0003-3507-5195

Boras Pucker 0000-0002-3321-7471

Elvira Hörandl 0000-0002-7600-1128

*** corresponding authors:** *email: kkarb@bgc-jena.mpg.de or kevin.karbstein@tu-ilmenau.de, elvira.hoerandl@biologie.uni-goettingen.de*

**Text S1.** (a, b) Extraction of genomic DNA (gDNA) and (b) library preparation for Oxford Nanopore Technology (ONT) sequencing performed at the University of Göttingen.

(a)

**gDNA extraction** (Qiagen Genomic Tip 20/G, high-molecular-weight DNA up to 150 kb; Qiagen, Hilden, Germany)

**Protocol based on**

- <https://www.qiagen.com/de/resources/download.aspx?id=cb2ac658-8d66-43f0-968e-7bb0ea2c402a&lang=en>
- the manufacturer´s instructions of the Qiagen Genomic DNA Buffer Set
- Vaillancourt and Buell (2019), Li et al. (2020)
- own experience

1. **Harvest young(!), fresh, dark-adapted leaves from your samples** (young expanding leaf and/or shoot material is optimal and should be dark-treated for 12 ~ 24 h before harvesting to reduce photosynthetic by-products; use a Falcon tube wrapped in aluminum foil for fresh leaf sampling (see also Li et al., 2020)
2. **Place autoclaved mortar and pestle (one set per sample) in the freezer just prior to gDNA extraction.** This avoids the immediate melting of ground frozen leaf powder and ensures high quality of extracted DNA (see below).
3. **Be sure to wear a lab coat, safety glasses, and gloves for any subsequent lab work!**
4. **Finally (!) grind 75 mg fresh leaf material** (should result in **at least** 30 ng/µl in 200 µl ddH_2_O, e.g., Qiagen AE buffer leads to bad Nanodrop 260/230 values due to various ingredients, like e.g. salt, which can be problematic in subsequent DNA sequencing; 🡪 30 ng/µl * 200 µl= 6000 ng 🡪 6 µg 🡪 Genomic-tip 20/G: up to 20 µg) **using liquid nitrogen and autoclaved mortar and pestle. The amount of fresh material is species-specific,** and 75 mg is needed to recover 6 µg of DNA from *Ranunculus auricomus* plants. **Pour a little liquid nitrogen into the empty mortar** and wait for it to evaporate (to cool), then add a little liquid nitrogen and then the leaves and **start grinding immediately**. Repeat this step if necessary. In the end, you should have a **finely ground powder that is still frozen**.
5. **Immediately(!) transfer the leaf powder** with a spatula into a 2.0 ml Eppendorf tube containing 400 µl of pre-warmed (40°C) Quiagen AP1 Extraction Buffer.
6. Incubate for 1.5 h at 40°C with gentle agitation (ca. 350 rpm).
7. Add **4 µl DNase-free Qiagen RNase A** (100 mg/ml), invert 20 times, and incubate for 30 minutes at 37°C.
8. Add **18 µl Qiagen Proteinase K** (20 mg/ml), invert 20 times, and incubate for 2 h at 50°C with gentle agitation.
9. **Centrifuge at 12,000 x g for 20 minutes** to pellet insoluble debris.
10. **Be sure to use wide-bore pipettes** to avoid disrupting the DNA molecules!
11. **Equilibrate the Qiagen Genomic Tip 20/G with 1 ml of QTB Buffer,** and allow the Qiagen Genomic Tip to empty by gravity flow.
12. Transfer **400 µl of the clarified lysate** (do not include the foam that formed during incubation time) to the appropriate buffer **QBT-equilibrated Qiagen Genomic Tip 20/G**. Allow it to enter the resin (Granulat, Harz) by gravity flow. **Important note: Keep the residue!** If you use too much fresh material, it is possible that the DNA won't be retained by the tip filters. You can recognize this problem in Step 15 because there is no precipitate. Just use the residue from this step and continue with step 15. Since you are transferring only precipitated DNA to your glass tube, no contaminants will be transferred to the next tube.
13. Wash with **QC Buffer** (Genomic-tip 20/G: **5 x 1 ml)** (**Important Note:** Keep the residue as well!).
14. Elute with prewarmed (55°C) **Buffer QF** (Genomic-tip 20/G: **0.8 ml**) into a new 2.0 ml Eppendorf Tube.
15. **Precipitate the DNA** by adding 0.7 volumes (ca. 0.5 ml) of room temperature isopropanol by inverting the tube 20 times, and waiting 15 minutes (DNA should usually precipitate immediately; **if this is not the case, the DNA was already solubilized in the previous steps** **🡪 see comments above**).
16. **Spool out DNA on a glass rod** (Alternatively: centrifuge at 5,500 g at 4°C for 30 minutes, and remove isopropanol).
17. Prepare a 1.5 ml Eppendorf tube with ca. 0.8 ml ice-cold 80% ethanol, and **wash the DNA (will be kept on the glass rod).**
18. **Air-dry the DNA (30 seconds) and gently resuspend the DNA in 200 µl Qiagen TE Buffer overnight in the refrigerator (4 °C**) (Alternatively, gently pipette the solution up and down several times 4-5 hours after extraction).
19. **Store at 4°C in the refrigerator for** several weeks or months. **Freeze the DNA sample only in case of long-term storage** (Freezing 🡪 DNA degradation!).

**Nanodrop (DNA Quality)**

1. Turn on the computer and click on the NanoDrop 2000 Spectrophotometer icon on the desktop.
2. Click on the Nucleic Acid tab.
3. Blank the machine before measuring concentrations (e.g., with 1.5 µl ddH2O/AE water).
4. Add 1.5 µl sample volume (shake very gently before).

**Gel electrophoresis (DNA Quality and Quantity)**

**Gel preparation:**

1. Weigh 1 g agarose and add to 70 mL 1 x TAE buffer (2%).
2. Boil (microwave).
3. Cool under running water.
4. Pour the gel solution into the gel stand and remove bubbles.
5. Place combs on the gel stand.
6. Place the hardened gel (after approx. 30 minutes) with the gel slide in the gel chamber.

**Sample preparation:**

1. Mix 5 µl of the extracted DNA with 1 µl application buffer (6x ROTI Load DNAstain; Carl Roth, Germany), respectively.

**Gel electrophoresis:**

1. Pipette samples into the gel chambers, and note the sample order.
2. Pipette 3 µl DNA size standard (Quick-Load 1 kb Extended DNA Ladder BioLabs) mixed with 1 µl loading buffer (6x ROTI Load DNAstain) into an empty chamber.
3. Gel run: 50 V, 100 minutes.
4. Take an image of the gel under UV light.

**Qubit (DNA Quantity;** Thermo Fisher Scientific, Waltham, USA)

**Sample preparation:**

1. All reagents should be at room temperature!
2. “Working solution” for MasterMix: **per sample and standard measurement,** **199 µl** dsDNA HS Buffer + **1 µl** dsDNA HS („high sensitive“) / BR (“broad range”) Reagent.
3. Qubit tubes: pipette **2 µl** DNA extract into the tube (vortex DNA before!) and add **198 µl** HS/BR Buffer 🡪 immediately vortex!
4. Pipette **10 µl** S1 and S2 HS/BR standard into a tube and add **190 µl** HS/BR Buffer 🡪 immediately vortex!
5. Incubate all qubit tubes protected from light for **at least 5 minutes**.

**Qubit measurements:**

1. Set DS-DNA (high sensitive/broad range) in the Qubit Fluorometer
2. Calibration: first measure Standard S1, then measure Standard S2
3. Sample measurements (invert tube 2-3 times before measurement)

(b)

Three gDNA extractions were performed in parallel from a single *R. cassubicifolius* s.l. individual (LH040). Fresh leaf material (ca. 75 mg) was frozen in liquid nitrogen and ground with a mortar and pestle, and transferred into a 2.0 ml Eppendorf tube containing 400 µl API Qiagen extraction buffer (Qiagen DNeasy Plant Mini Kit). The mixture was incubated at 40°C for 1.5 hours with gentle agitation. Then, 4 µl Qiagen RNase A (100 mg/ml) was added, and the mixture was incubated again at 37°C for 30 minutes, followed by the addition of 18 µl Qiagen Proteinase K (20 mg/ml), and incubation at 50°C for 2 h with gentle agitation. The mixture was centrifuged at 12,000 x g for 20 min, and ca. 400 µl of the lysate was transferred with wide-bore tips to a buffer QBT-equilibrated Genomic Tip. DNA was washed with 5 x 1 ml QC Buffer, and eluted with 0.8 ml 55°C pre-warmed QF Buffer into a new 2.0 ml Eppendorf tube. DNA was precipitated by adding 70% room-temperature isopropanol and inverting the tube ca. 20 times, followed by incubation at room temperature for 15 min. Using a glass rod, the DNA was rolled up and placed into a new 1.5 ml Eppendorf tube containing ca. 0.8 ml ice-cold 80% ethanol. The DNA was air dried, resuspended in 200 µl autoclaved ddH_2_O, and all three extractions were pooled into a single tube, stored at 4 °C until library preparation.

DNA concentration was assessed using the Qubit Fluorometer 3.0 and the Qubit dsDNA HS Assay Kit, and the DNA purity was checked with the NanoDrop 2000 Spectrophotometer (ThermoFisher Scientific, Waltham, USA; Text S1). DNA fragment length distribution and RNA absence were checked on a 2% agarose gel via electrophoresis (50 V, 90 min) and the Quick-Load 1 kb DNA Ladder (New England Biolabs Inc., Ipswich, USA; Text S1, Figure S1). After several dilution steps, we obtained a final DNA yield of 61.2 µg (51.0 ng/µl * 1200 µl ddH_2_O).

Library preparation was conducted using the ONT Ligation Sequencing Kit SQK-LSK110 optimized for high throughput and long reads (ONT, Oxford, UK), and applicable for singleplex gDNA sequencing. We adjusted the DNA concentration to 1000 ng in 47 µl (ca. 21.5 µl/ng) and followed the manufacturer’s instructions for library preparation (protocol vGDE_9108_v110_revL_10Nov2020, available at community.nanoporetech.com) with a few modifications. Incubation times were increased up to 15 min, the concentration of ethanol wash buffer was increased to 80%, and DNA fragments > 3 kbp were enriched using the buffers provided with the kit. Active pores ranged from ca. 800-1500. Flow cell 1 was loaded with freshly prepared libraries, flow cell 2 with one freshly prepared and one library stored at -80°C for two days, and flow cells 3-6 with two libraries stored at -80°C for 1-2 weeks. Freezing of gDNA had no apparent effect on N_50_ fragment size (see also Results & Discussion and flow cell reports, item [04] on FigShare).

**Text S2**. Determining the optimal DNA sequence alignment for phylogenetic analyses.

Filtering settings (i.e., the minimum number of samples per alignment site) resulted in different alignment lengths: 343,436 bp (min0), 155,794 (min50), 147,062 (min70), and 126,707 bp (min90). With increasing strictness of data filtering (min0 to min90), we observed an increase in mean FBP (86.09, 88.06, 88.31, and 91.21) and TBE (93.43, 94.36, 93.92, and 95.56) values. As expected for large phylogenies, TBE was higher than FBP (88.42 vs. 94.32). Mean values of QS metrics such as QC (0.66, 0.67, 0.66, and 0.67) and QI (0.90, 0.91, 0.91, and 0.90) showed relatively high levels of concordant patterns with taxa usually “placed” correctly within the phylogeny and high branch informativeness across filtering settings, although a drop in median QC for min0 (QC=0.92, all others with QC and QI = 1) was observed. The raw (min0) and less strictly filtered alignments (min50, min70) contained various gaps due to substantial variation in non-coding plastome sequence regions. While this information can be useful for discriminating closely related taxa, it has also been shown that random or too high levels of missing data negatively affect phylogenetic reconstructions and bootstrap values (Eaton et al., 2017; Karbstein, Tomasello et al., 2020). Therefore, we chose the min90 setting (126,707 bp) with the lowest number of gaps in the alignment, excluding poorly assembled non-coding DNA regions, and the highest average bootstrap support in the phylogeny.

**Text S3.** Identification of tandem repeats (TRs) and transposable elements (TEs), and protein-coding genes.

The genome assembly was subjected to the identification of tandem repeats and transposable elements (TEs) using Tandem Repeats Finder v4.10.1 (Benson, 1999) with parameters: ‘Match=2, Mismatch=5, Delta=7, PM=80, PI=10, Minscore=5, and MaxPeriod=2000’ and EDTA v2.2.1, respectively. The EDTA pipeline integrates the results of multiple tools: LTR-FINDER (Xu and Wang, 2007; Ou and Jiang, 2019), LTR_retriever (Ou and Jiang, 2018), Generic Repeat Finder (Shi and Liang, 2019), TIR-Learner (Xiong et al., 2014; Su et al., 2019), HelitronScanner (Xiong et al., 2014; Zhang et al., 2022), and TEsorter, to achieve accurate and efficient TE identification and classification.

To identify inter- and intra-chromosomal syntenic regions, MMseqs2 was used. First, the *Ranunculus cassubicifolius* genome sequence was converted into the MMseqs2 database format followed by the identification of similar sequences with the parameters --start-sens 1 --sans-steps 2 -s 4. The resulting database was converted into BLAST output format 6. A custom python script (<https://github.com/NancyChoudhary28/Ranunculus-genomics>) was used to filter sequences based on two criteria, (a) sequence identity more than 90%, and (b) sequence length of at least 5000 bp.

Given the limited availability of RNA-seq reads from *R. cassubicifolius*, we used a multi-species RNA-seq mapping approach. This strategy included RNA reads from multiple *Ranunculus* species (37 individuals, Table S2), retrieved using prefetch and fasterq-dump from NCBI SRA toolkit v3.1.0. RNA data was mapped onto the genome using HISAT2 v2.2.1 (Kim et al., 2019).

Structural annotation of protein-coding genes used a combination of three approaches. (i) First, homology-based gene prediction using GeMoMa v1.9 (Keilwagen et al., 2016, 2018), using annotations from three Ranunculaceae and eleven Ranunculales species (Table S3). The coding genes from these 14 species were aligned to the *R. cassubicifolius* genome sequence using MMseqs2 v15-6f452 (Steinegger and Söding, 2017) following the default GeMoMa setting. GeMoMa is based on the amino acid sequences and intron position conservation among closely related species. To refine intron boundaries, RNA-seq data from five recently published diploid *R. auricomus* transcriptomes (Paetzold et al., 2022) were incorporated. The resulting fourteen gene annotation sets were merged and filtered using the GeMoMa Annotation Filter (GAF) with parameters: f="start=='M' and stop=='*' and (isNaN(score) or score/aa>=1.50 or (score/aa>0.8 and avgCov>50)) and avgCov>0.0 and (iAA>=0.5 or pAA>=0.5) and (evidence>1 or tpc>=0.8)" and atf="tie==1 and sumWeight>4".

(ii) Second, we used the BRAKER3 pipeline (Gabriel et al., 2024), which uses *ab initio* gene prediction using AUGUSTUS v3.5.0 (Stanke et al., 2006, 2008) and GeneMark-ETP v1.02 (Brůna et al., 2024), which is corrected by extrinsic evidence provided by RNA-seq and protein homology. For protein evidence, we used the ‘Viridiplantae’ library (Kuznetsov et al., 2023) and annotated NCBI genomes of Ranunculaceae species (*Aquilegia caerulea* [GCA_002738505.1], *Thalictrum thalictroides* [GCA_013358455.1], and *Coptis chinensis* [GCA_015680905.1]). The resulting BRAKER3 annotation was filtered using GeMoMa Annotation Filter (GAF) with parameters: f="start=='M' and stop=='*' and avgCov>0.0 and tpc>0.5" and atf="tie==1 or sumWeight>1".

(iii) Third, we used Funannotate v1.8.15 (<https://github.com/nextgenusfs/funannotate>; Palmer and Stajich, 2022), initially developed for fungi but now capable of handling larger genomes. Repetitive contigs were cleaned from the genome using minimap2 v2.26 and simple repeats were masked using TANTAN (‘-s arabidopsis’; Frith, 2011). Approximately 8.04% (217 Mbp) of the genome sequence was masked. Funannotate’s gene prediction involved three steps: train, predict, and update. In all steps, the parameter ‘--max_intronlen 1000000’ was used. The filtered genome and the transcriptome sequences were used to train the annotation process by a genome-guided transcriptome assembly using Trimmomatic v0.39 (Bolger et al., 2014), Trinity v2.8.5 (Grabherr et al., 2011), HISAT2, kallisto v0.46.1 (Bray et al., 2016), and PASA v2.5.3 (Haas et al., 2008) to filter, normalize, and cluster (genome-guided) transcriptomic data. The sorted alignments, transcripts, UniProt protein library, and transcriptome annotations were the input for the gene prediction process performed by AUGUSTUS v3.5.0, GlimmerHMM (Majoros et al., 2004), and SNAP (Korf, 2004), and results were summarized with EVidenceModeler v1.1.1 (Haas et al., 2008). Additional parameters ‘--busco_db eudicots_odb10 --organism other --busco_seed_species arabidopsis’ were used in this step.

The annotations resulting from GeMoMa, BRAKER3, and Funannotate were compared based on BUSCO completeness scores. Based on these comparisons, the GeMoMa and BRAKER3 gene predictions were merged using GeMoMa Annotation Filter (GAF) with the following parameters: f="start=='M' and stop=='*' and avgCov>0 and tpc>0 and aa >=50 and ((isNaN(score) and (tie==1 or isNaN(tie) and tpc>0.5)) or (tie>0.5 or tpc>0.5))" and atf= " tie==1 and tpc>0.5 and (isNaN(score) or sumWeight>2)". Gene models in disagreement with RNA evidence data were fixed manually. The annotation quality was evaluated using BUSCO v5.6.1 with the embryophyta_odb10 database. The functional annotation was performed using InterProScan5 (Jones et al., 2014) and eggNOG-mapper (Cantalapiedra et al., 2021) using the Funannotate “annotate” function.

**Text S4**. Detailed results of plastome-based phylogenies in Ranunculaceae.

The results of the best maximum likelihood plastome analyses indicate a fully resolved and well supported phylogeny (TBE>95; Figures 3, S2 & S4). Low branch support occurs particularly in the species-rich genera *Anemone, Clematis, Hepatica,* or *Aconitum* probably due to fast radiation and slow rates of plastome sequence evolution (Wang et al., 2016; Zhai et al., 2019). Almost all subfamilies/tribes are monophyletic (except for *Anemoneae* and *Adonideae*; also a few species are non-monophyletic like *Clematis fruticosa* or *terniflora*). Convergent evolution with multiple origins of taxonomically informative morphological characters has been shown for Ranunculaceae (Zhai et al., 2019) and may explain non-monophyletic relationships observed particularly for *Adonideae*. The ambiguous position of the local endemic of the Qinling Mountains in China *Anemone taipaiensis*, which leads to the non-monophyly of *Anemoneae* (but monophyletic in less well-supported min50/70 phylogenies, Figure S2bc), needs further investigation.

The tribes branch successively as follows: *Glaucideae*, *Hydrastideae*, and *Coptideae*, and (the core Ranunculaceae) *Adonideae* (incl. *Trollius* and *Calathodes*), *Thalictroideae*, *Caltheae*, *Asteropyreae*, *Cimifugeae*, *Nigelleae*, *Delphinieae*, *Calanthemeae*, *Helleboreae*, *Ranunculeae*, *Adonideae* (incl. *Adonis*), and *Anemoneae*. Observed phylogenetic patterns among tribes are largely consistent across min0 to min90 datasets (except for ‘jumping’ tribes such as *Cimifugeae* and a part of *Thalictroideae*; Figure S2a-d), indicating a robust phylogenetic framework for Ranunculaceae. Branching of tribes is also widely consistent with a recent plastome study based on fewer taxa or a multi-locus-based nuclear and plastid phylogeny (Wang et al., 2009; Zhai et al., 2019; except for non-monophyly of *Adonideae* and sister relationships among “central” tribes such as *Delphinieae*, *Nigelleae, Cimifugeae, Caltheae*, and *Thalictroideae*).

**Text S5.** Impact of using frozen libraries for ONT DNA sequencing.

Sequencing of ONT libraries of flow cells 3-6 once stored at -80°C compared to fresh libraries of flow cell 1 (and partly 2) did not yield decreased output quality in terms of N_50_ read fragment size (flow cells 1-6: 20.1, 21.67, 21.6, 20.5, 19.6, and 23.3 kbp), and read number and sequencing output (flow cells 1-6: 1.1, 0.7, 0.9, 0.5, 1.8, and 0.7 M / 10.8, 6.6, 7.3, 3.3, 11.7, and 5.7 Gbp). Flow cell reports are deposited in item [04] on FigShare. Therefore, short freezing and thawing of the DNA libraries did not lead to any apparent fragmentation of the DNA reads, supported by observations for dried material or general extraction recommendations (Doyle and Dickson, 1987; Russo et al., 2022). This may be attributed to the used standard elution buffer that stabilizes DNA in solution, and the rapid freezing at -80°C and slow thawing of libraries.

In addition, it is important to note that the ONT read N_50_ length of the runs in this study was ca. 20 kbp, while other studies have achieved runs with more than twice this value when using fresh libraries based on different DNA extraction protocols (Hakim et al., 2024; Horz et al., 2024; Nowak et al., 2024). Using the latest ONT flow cell generation and fresh DNA extracts from different kits would probably deliver much longer, higher quality reads and thus improved assemblies.

**Table S1.** Selected (a) plastome and (b) mitogenome sequences from NCBI.

**(a)**

| **Genbank ID** | **Taxon** | **Sequence Length (bp)** |
| --- | --- | --- |
| PP407389 | *Aconitum alboviolaceum* | 155652 |
| NC_036357 | *Aconitum angustius* | 156109 |
| KY407559 | *Aconitum austrokoreense* | 155892 |
| MK253470 | *Aconitum barbatum* | 156761 |
| NC_041579 | *Aconitum brachypodum* | 155651 |
| OK323949 | *Aconitum bulleyanum* | 155791 |
| KY407560 | *Aconitum carmichaelii* | 155880 |
| NC_031420 | *Aconitum ciliare* | 155832 |
| MG678803 | *Aconitum contortum* | 155653 |
| NC_031421 | *Aconitum coreanum* | 157029 |
| OM289058 | *Aconitum delavayi* | 155733 |
| NC_061703 | *Aconitum duclouxii* | 155479 |
| NC_038096 | *Aconitum episcopale* | 151214 |
| NC_036358 | *Aconitum finetianum* | 155625 |
| NC_056280 | *Aconitum flavum* | 155654 |
| MZ959044 | *Aconitum forrestii* | 155869 |
| OK539525 | *Aconitum habaense* | 155800 |
| NC_038095 | *Aconitum hemsleyanum* | 155684 |
| KT820669 | *Aconitum jaluense subsp. jaluense* | 155926 |
| KT820670 | *Aconitum japonicum subsp. napiforme* | 155878 |
| MK253471 | *Aconitum kusnezoffii* | 155832 |
| NC_035894 | *Aconitum longecassidatum* | 155524 |
| NC_080244 | *Aconitum macrorhynchum* | 155913 |
| NC_031423 | *Aconitum monanthum* | 155688 |
| NC_061321 | *Aconitum nagarum* | 155732 |
| NC_061553 | *Aconitum ouvrardianum* | 155799 |
| MK782812 | *Aconitum paniculigerum var. wulingense* | 155774 |
| MK782813 | *Aconitum paniculigerum var. wulingense* | 155813 |
| NC_053848 | *Aconitum pendulum* | 155597 |
| NC_058692 | *Aconitum piepunense* | 155836 |
| NC_035892 | *Aconitum pseudolaeve* | 155628 |
| MN967020 | *Aconitum puchonroenicum* | 155631 |
| NC_057536 | *Aconitum quelpaertense* | 155636 |
| NC_061700 | *Aconitum ramulosum* | 155841 |
| MF186593 | *Aconitum reclinatum* | 157354 |
| MW817090 | *Aconitum scaposum* | 157688 |
| PP407390 | *Aconitum sczukinii* | 155819 |
| NC_036359 | *Aconitum sinomontanum* | 157215 |
| NC_061555 | *Aconitum stapfianum* | 155858 |
| NC_061704 | *Aconitum stylosum* | 155475 |
| NC_050689 | *Aconitum tanguticum* | 157114 |
| ON751949 | *Aconitum transsectum* | 155872 |
| NC_066973 | *Aconitum tschangbaischanense* | 155881 |
| NC_072898 | *Aconitum umbrosum* | 157227 |
| NC_038094 | *Aconitum vilmorinianum* | 155761 |
| KU556690 | *Aconitum volubile* | 155872 |
| NC_061702 | *Aconitum weixiense* | 155872 |
| NC_041525 | *Actaea asiatica* | 159638 |
| NC_081474 | *Actaea biternata* | 159761 |
| NC_077574 | *Actaea cimicifuga* | 159761 |
| NC_041533 | *Actaea dahurica* | 159370 |
| NC_042253 | *Actaea heracleifolia* | 159578 |
| NC_081475 | *Actaea japonica* | 159736 |
| NC_081476 | *Actaea purpurea* | 159497 |
| MN623225 | *Actaea simplex* | 159624 |
| NC_041543 | *Actaea vaginata* | 159110 |
| NC_056353 | *Adonis amurensis* | 157032 |
| MK253469 | *Adonis coerulea* | 157033 |
| NC_077573 | *Adonis mongolica* | 157521 |
| NC_065037 | *Adonis pseudoamurensis* | 156917 |
| NC_072342 | *Adonis ramosa* | 157057 |
| NC_041474 | *Adonis sutchuenensis* | 157603 |
| NC_037194 | *Anemoclema glaucifolium* | 160400 |
| NC_063528 | *Anemone alpina* | 162638 |
| NC_064389 | *Anemone altaica* | 160806 |
| NC_082177 | *Anemone coronaria* | 159564 |
| NC_060982 | *Anemone flaccida* | 157213 |
| NC_039465 | *Hepatica henryi* | 159276 |
| NC_045910 | *Hepatica asiatica* | 160157 |
| NC_045878 | *Hepatica nobilis var. japonica* | 160988 |
| NC_066445 | *Anemone hortensis* | 158962 |
| NC_045909 | *Hepatica maxima* | 160876 |
| NC_045879 | *Anemone narcissiflora* | 158557 |
| NC_066446 | *Anemone nemorosa* | 160895 |
| NC_063529 | *Anemone occidentalis* | 161678 |
| MN025345 | *Anemone patens subsp. multifida* | 161936 |
| MN025344 | *Pulsatilla grandis* | 161376 |
| NC_041526 | *Anemone raddeana* | 160493 |
| NC_054280 | *Anemone reflexa* | 160910 |
| OR251125 | *Anemone rivularis var. flore-minore* | 162991 |
| NC_069812 | *Anemone shikokiana* | 159286 |
| NC_050873 | *Anemone taipaiensis* | 162084 |
| OR251126 | *Anemone thomsonii var. thomsonii* | 160058 |
| NC_039451 | *Anemone tomentosa* | 160945 |
| NC_039456 | *Anemone trullifolia* | 157096 |
| MK860686 | *Anemone turczaninovii* | 162795 |
| NC_056381 | *Anemone vitifolia* | 162203 |
| NC_041527 | *Anemonopsis macrophylla* | 158787 |
| NC_053820 | *Aquilegia barnebyi* | 161954 |
| NC_041528 | *Aquilegia coerulea* | 161429 |
| NC_041529 | *Aquilegia ecalcarata* | 161795 |
| NC_058528 | *Aquilegia kansuensis* | 162090 |
| NC_046738 | *Aquilegia rockii* | 162123 |
| NC_058527 | *Aquilegia yabeana* | 162094 |
| NC_058529 | *Aquilegia yangii* | 161820 |
| NC_041530 | *Asteropyrum cavaleriei* | 163660 |
| NC_045850 | *Asteropyrum peltatum* | 164455 |
| NC_041531 | *Beesia calthifolia* | 158355 |
| NC_072729 | *Beesia deltophylla* | 157506 |
| OY747139 | *Berberis vulgaris* | 166264 |
| NC_041475 | *Calathodes oxycarpa* | 160415 |
| MK253466 | *Callianthemum alatavicum* | 156538 |
| NC_041476 | *Callianthemum taipaicum* | 156939 |
| NC_041532 | *Caltha palustris* | 156167 |
| MK253464 | *Ceratocephala falcata* | 150821 |
| PP155435 | *Ceratocephala orthoceras* | 151407 |
| NC_069854 | *Ceratocephala testiculata* | 150820 |
| NC_046829 | *Chelidonium majus* | 159734 |
| NC_039844 | *Clematis acerifolia* | 159552 |
| NC_065272 | *Clematis acerifolia var. elobata* | 159690 |
| MT876505 | *Clematis alpina subsp. ochotensis* | 159631 |
| MT876514 | *Clematis alpina subsp. sibirica* | 159675 |
| NC_039577 | *Clematis alternata* | 159476 |
| NC_081049 | *Clematis apiifolia* | 159682 |
| NC_069848 | *Clematis armandii* | 159353 |
| NC_069849 | *Clematis aureolata* | 159548 |
| NC_065250 | *Clematis brachiata* | 159678 |
| NC_042793 | *Clematis brachyura* | 159532 |
| NC_039579 | *Clematis brevicaudata* | 159583 |
| NC_065251 | *Clematis cadmia* | 159714 |
| NC_066977 | *Clematis calcicola* | 159658 |
| NC_065264 | *Clematis campestris* | 159671 |
| NC_065265 | *Clematis canescens* | 159752 |
| NC_066718 | *Clematis chinensis* | 159497 |
| NC_065266 | *Clematis chrysocoma* | 159584 |
| NC_065267 | *Clematis connata* | 159768 |
| NC_065268 | *Clematis crassifolia* | 159312 |
| NC_065269 | *Clematis crispa* | 159304 |
| NC_065270 | *Clematis delavayi* | 159659 |
| NC_065271 | *Clematis drummondii* | 159729 |
| NC_058885 | *Clematis florida* | 159606 |
| NC_065273 | *Clematis fruticosa* | 159796 |
| NC_060524 | *Clematis fusca* | 159624 |
| NC_065274 | *Clematis glaucophylla* | 159302 |
| NC_081050 | *Clematis gouriana* | 159681 |
| NC_081051 | *Clematis grandidentata* | 159634 |
| NC_081052 | *Clematis gratopsis* | 159643 |
| NC_050373 | *Clematis guniuensis* | 159682 |
| NC_065275 | *Clematis haenkeana* | 156007 |
| NC_070190 | *Clematis henryi* | 159707 |
| NC_039845 | *Clematis heracleifolia* | 159565 |
| NC_065276 | *Clematis hexapetala* | 159534 |
| NC_065277 | *Clematis huchouensis* | 159590 |
| NC_081053 | *Clematis integrifolia* | 159630 |
| NC_065278 | *Clematis intricata* | 159593 |
| NC_065279 | *Clematis lancifolia* | 159447 |
| NC_065286 | *Clematis lasiandra* | 159688 |
| NC_065281 | *Clematis leschenaultiana* | 159622 |
| NC_065282 | *Clematis ligusticifolia* | 159600 |
| NC_039690 | *Clematis loureiroana* | 159624 |
| NC_041477 | *Clematis macropetala* | 159647 |
| NC_079957 | *Clematis mandshurica* | 159564 |
| NC_057507 | *Clematis montana* | 159523 |
| NC_065283 | *Clematis nannophylla* | 159814 |
| NC_071961 | *Clematis orientalis* | 159543 |
| NC_069850 | *Clematis otophora* | 159534 |
| NC_081054 | *Clematis parviloba* | 159664 |
| NC_063559 | *Clematis patens* | 159603 |
| NC_085220 | *Clematis patens subsp. tientaiensis* | 159644 |
| NC_081055 | *Clematis peterae* | 159676 |
| NC_065284 | *Clematis petriei* | 159425 |
| NC_065248 | *Clematis pinnata* | 159652 |
| NC_058760 | *Clematis potaninii* | 159691 |
| NC_081056 | *Clematis psilandra* | 159622 |
| NC_065285 | *Clematis pubescens* | 159391 |
| NC_065287 | *Clematis quinquefoliolata* | 159724 |
| NC_081994 | *Clematis ranunculoides* | 159741 |
| NC_069851 | *Clematis rehderiana* | 159582 |
| NC_039578 | *Clematis repens* | 159507 |
| NC_065288 | *Clematis reticulata* | 159284 |
| NC_065289 | *Clematis rutoides* | 159678 |
| NC_060523 | *Clematis serratifolia* | 159648 |
| NC_065290 | *Clematis songorica* | 159822 |
| NC_081062 | *Clematis speciosa* | 159753 |
| NC_081057 | *Clematis stans* | 159671 |
| ON411443 | *Clematis stans var. austrojaponensis* | 159775 |
| NC_081058 | *Clematis subumbellata* | 159687 |
| NC_080984 | *Clematis tangutica* | 159584 |
| NC_028000 | *Clematis terniflora* | 159528 |
| NC_069852 | *Clematis tibetana* | 159540 |
| NC_065291 | *Clematis tomentella* | 159818 |
| NC_081059 | *Clematis tsugetorum* | 159611 |
| NC_065249 | *Clematis tubulosa* | 159653 |
| OP649578 | *Clematis tubulosa var. ichangensis* | 159645 |
| NC_039846 | *Clematis uncinata* | 159524 |
| NC_065292 | *Clematis urophylla* | 159696 |
| NC_081060 | *Clematis urticifolia* | 159720 |
| NC_065293 | *Clematis virginiana* | 159561 |
| NC_065294 | *Clematis viridis* | 159847 |
| NC_081061 | *Clematis vitalba* | 159710 |
| NC_065295 | *Clematis williamsii* | 159645 |
| NC_065296 | *Clematis xiangguiensis* | 159575 |
| NC_041534 | *Consolida ajacis* | 155900 |
| NC_047292 | *Consolida orientalis* | 155915 |
| NC_036485 | *Coptis chinensis* | 155484 |
| OM202495 | *Coptis chinensis var. brevisepala* | 154641 |
| MK569483 | *Coptis chinensis* | 154849 |
| NC_064406 | *Coptis deltoidea* | 154538 |
| NC_054329 | *Coptis japonica* | 154985 |
| NC_054330 | *Coptis omeiensis* | 154573 |
| NC_037759 | *Coptis quinquesecta* | 154549 |
| NC_054331 | *Coptis teeta* | 154156 |
| MK253461 | *Delphinium anthriscifolium* | 155077 |
| NC_051554 | *Delphinium brunonianum* | 153926 |
| MW246165 | *Delphinium candelabrum var. monanthum* | 153995 |
| MK253460 | *Delphinium ceratophorum* | 154245 |
| NC_049872 | *Delphinium grandiflorum* | 157339 |
| NC_047293 | *Delphinium maackianum* | 154484 |
| NC_056321 | *Delphinium yunnanense* | 154053 |
| MK253459 | *Dichocarpum dalzielii* | 153110 |
| NC_041478 | *Dichocarpum fargesii* | 153134 |
| MK253458 | *Dichocarpum sutchuenense* | 155390 |
| NC_041535 | *Enemion raddeanum* | 151916 |
| NC_053526 | *Epimedium parvifolium* | 157201 |
| NC_066652 | *Eranthis byunsanensis* | 160324 |
| NC_041536 | *Eranthis stellata* | 159251 |
| MK281585 | *Eschscholzia californica* | 160201 |
| PP155436 | *Ficaria verna* | 156439 |
| NC_041539 | *Glaucidium palmatum* | 156791 |
| NC_033341 | *Gymnaconitum gymnandrum* | 157327 |
| NC_062141 | *Helleborus atrorubens* | 166695 |
| NC_041540 | *Helleborus thibetanus* | 155525 |
| NC_060983 | *Hepatica acutiloba* | 159497 |
| NC_060984 | *Hepatica americana* | 159805 |
| NC_060985 | *Hepatica falconeri* | 161075 |
| MG001340 | *Hepatica henryi* | 159276 |
| NC_062086 | *Hepatica insularis* | 160377 |
| MG952899 | *Hepatica maxima* | 160876 |
| NC_060986 | *Hepatica nobilis* | 160635 |
| MG952898 | *Hepatica nobilis var. japonica* | 160988 |
| NC_060987 | *Hepatica transsilvanica* | 161005 |
| NC_034702 | *Hydrastis canadensis* | 160000 |
| NC_041541 | *Isopyrum manshuricum* | 151243 |
| NC_041542 | *Leptopyrum fumarioides* | 157448 |
| NC_066184 | *Mahonia bodinieri* | 165697 |
| NC_012615 | *Megaleranthis saniculifolia* | 159924 |
| PP155437 | *Myosurus apetalus* | 150380 |
| PP155438 | *Myosurus minimus* | 150431 |
| NC_039542 | *Naravelia pilulifera* | 159513 |
| NC_039580 | *Naravelia zeylanica* | 159568 |
| NC_041537 | *Nigella damascena* | 155218 |
| NC_083219 | *Nigella sativa* | 154120 |
| NC_041538 | *Oxygraphis glacialis* | 155434 |
| NC_037831 | *Papaver rhoeas* | 152905 |
| NC_041479 | *Paraquilegia anemonoides* | 164383 |
| NC_067755 | *Paraquilegia microphylla* | 164390 |
| NC_061038 | *Pulsatilla campanella* | 162322 |
| NC_045908 | *Pulsatilla cernua var. koreana* | 162709 |
| NC_039452 | *Pulsatilla chinensis* | 162052 |
| MK860685 | *Pulsatilla dahurica* | 162450 |
| OP729488 | *Pulsatilla saxatilis* | 162659 |
| NC_061398 | *Pulsatilla tongkangensis* | 163442 |
| KX639503 | *Ranunculus austro-oreganus* | 154493 |
| MK253468 | *Ranunculus bungei* | 156082 |
| NC_045920 | *Ranunculus cantoniensis* | 155154 |
| NC_077490 | *Ranunculus cassubicifolius* | 156233 |
| NC_079824 | *Ranunculus chinensis* | 155289 |
| KY562595 | *Ranunculus flammula* | 156061 |
| MZ169045 | *Ranunculus japonicus* | 156981 |
| NC_008796 | *Ranunculus macranthus* | 155129 |
| NC_065303 | *Ranunculus membranaceus* | 156028 |
| NC_031651 | *Ranunculus occidentalis* | 154474 |
| NC_060613 | *Ranunculus pekinensis* | 156139 |
| NC_036976 | *Ranunculus repens* | 154247 |
| NC_036977 | *Ranunculus reptans* | 157239 |
| NC_080350 | *Ranunculus sceleratus* | 156329 |
| ON462450 | *Ranunculus silerifolius var. silerifolius* | 155368 |
| NC_081908 | *Ranunculus ternatus* | 156003 |
| NC_063514 | *Ranunculus yunnanensis* | 156050 |
| NC_039743 | *Semiaquilegia adoxoides* | 158340 |
| NC_057495 | *Semiaquilegia guangxiensis* | 164047 |
| NC_047294 | *Staphisagria macrosperma* | 155905 |
| NC_058830 | *Thalictrum aquilegiifolium* | 156253 |
| MW816628 | *Thalictrum aquilegiifolium var. sibiricum* | 156244 |
| MZ962406 | *Thalictrum baicalense* | 156196 |
| NC_061927 | *Thalictrum cirrhosum* | 155969 |
| NC_026103 | *Thalictrum coreanum* | 155088 |
| NC_085600 | *Thalictrum elegans* | 155864 |
| NC_070058 | *Thalictrum fargesii* | 155929 |
| NC_053570 | *Thalictrum foeniculaceum* | 155923 |
| NC_058920 | *Thalictrum foliolosum* | 155764 |
| OM501079 | *Thalictrum minus var. hypoleucum* | 156258 |
| NC_041544 | *Thalictrum minus* | 156217 |
| MK253449 | *Thalictrum petaloideum* | 155876 |
| NC_068627 | *Thalictrum simplex* | 156211 |
| MK253448 | *Thalictrum tenue* | 156103 |
| NC_039433 | *Thalictrum thalictroides* | 154924 |
| NC_058831 | *Thalictrum viscosum* | 155984 |
| NC_084427 | *Trollius altaicus* | 159986 |
| NC_084428 | *Trollius asiaticus* | 160002 |
| NC_084429 | *Trollius buddae* | 159688 |
| NC_031849 | *Trollius chinensis* | 160191 |
| NC_084430 | *Trollius dschungaricus* | 159816 |
| NC_050872 | *Trollius farreri* | 160611 |
| NC_084431 | *Trollius japonicus* | 160061 |
| NC_084432 | *Trollius ledebouri* | 160025 |
| NC_084433 | *Trollius lilacinus* | 160202 |
| NC_059918 | *Trollius macropetalus* | 160094 |
| NC_084434 | *Trollius micranthus* | 159597 |
| NC_084435 | *Trollius pumilus* | 159598 |
| NC_084436 | *Trollius ranunculoides* | 159616 |
| NC_084437 | *Trollius taihasenzanensis* | 160019 |
| NC_084438 | *Trollius vaginatus* | 159723 |
| NC_084439 | *Trollius yunnanensis* | 159717 |
| NC_039744 | *Urophysa henryi* | 158303 |
| NC_039742 | *Urophysa rockii* | 158512 |

**(b)**

| **Genbank ID** | **Taxon** | **Sequence Length (bp)** |
| --- | --- | --- |
| NC_084324 | *Aconitum carmichaelii* | 425319 |
| NC_053920 | *Aconitum kusnezoffii* | 440720 |
| NC_053368 | *Hepatica maxima* | 1122546 |
| OR100522 | *Corydalis pauciovulata* | 675483 |
| NC_072536 | *Paropyrum anemonoides* | 206722 |
| NC_068018 | *Pulsatilla cernua* | 747621 |
| NC_068017 | *Pulsatilla chinensis* | 878988 |
| OL979137 | *Pulsatilla chinensis var. kissii* | 684203 |
| NC_071219 | *Pulsatilla dahurica* | 824625 |
| NC_088759 | *Ranunculus cassubicifolius* | 1183731 |

**Table S2.** RNA-seq data of 37 Ranunculaceae individuals from SRA/NCBI used for *Ranunculus cassubicifolius* genome annotation. See Materials and Methods for annotation details.

| **SRA ID** | **Taxon** |
| --- | --- |
| SRR21162053 | *Ranunculus carpaticola x Ranunculus notabilis* |
| SRR21162054 | *Ranunculus carpaticola x Ranunculus notabilis* |
| SRR21162055 | *Ranunculus notabilis* |
| SRR21162056 | *Ranunculus notabilis* |
| SRR21162057 | *Ranunculus carpaticola* |
| SRR11487683 | *Ranunculus caucasicus* |
| SRR11487684 | *Ranunculus acris* |
| SRR11487685 | *Ranunculus acris* |
| SRR13060786 | *Ranunculus japonicus* |
| SRR1737526 | *Ranunculus cantoniensis* |
| SRR1822558 | *Ranunculus brotherusii* |
| SRR3291759 | *Ranunculus sceleratus* |
| SRR958803 | *Ranunculus carpaticola x Ranunculus cassubicifolius* |
| SRR958819 | *Ranunculus carpaticola x Ranunculus cassubicifolius* |
| SRR958841 | *Ranunculus notabilis* |
| SRR958846 | *Ranunculus cassubicifolius* |
| SRR1822529 | *Ranunculus bungei* |
| SRR18512371 | *Ranunculus bungei* |
| SRR18512372 | *Ranunculus bungei* |
| SRR18512373 | *Ranunculus bungei* |
| SRR18512374 | *Ranunculus bungei* |
| SRR18512375 | *Ranunculus bungei* |
| SRR18512376 | *Ranunculus bungei* |
| SRR18512381 | *Ranunculus bungei* |
| SRR18512383 | *Ranunculus bungei* |
| SRR18512384 | *Ranunculus bungei* |
| SRR18512385 | *Ranunculus bungei* |
| SRR18512386 | *Ranunculus bungei* |
| SRR18512387 | *Ranunculus bungei* |
| SRR18512388 | *Ranunculus bungei* |
| SRR18512389 | *Ranunculus bungei* |
| SRR18512390 | *Ranunculus bungei* |
| SRR18512391 | *Ranunculus bungei* |
| SRR18512392 | *Ranunculus bungei* |
| SRR18512398 | *Ranunculus bungei* |
| SRR18512400 | *Ranunculus bungei* |
| SRR18512401 | *Ranunculus bungei* |

**Table S3.** Genome annotation data of 14 Ranunculales species from NCBI used for genome annotation of *Ranunculus cassubicifolius*.

| **Tool** | **Species** | **Family** | **Order** | **NCBI Accession** | **Reference** |
| --- | --- | --- | --- | --- | --- |
| Funannotate and Braker | *Aquilegia coerulea* | Ranunculaceae | Ranunculales | GCA_002738505.1 | <https://www.ncbi.nlm.nih.gov/datasets/genome/GCA_002738505.1/> |
| Funannotate and Braker | *Coptis chinensis* | Ranunculaceae | Ranunculales | GCA_015680905.1 | <https://doi.org/10.1038/s41467-021-23611-0> |
| Funannotate and Braker | *Thalictrum thalictroides* | Ranunculaceae | Ranunculales | GCA_013358455.1 | <https://doi.org/10.1002/aps3.11407> |
| Funannotate | *Kingdonia uniflora* | Circaeasteraceae | Ranunculales | GCA_014058105.1 | <https://doi.org/10.1016/j.isci.2020.101124> |
| Funannotate | *Macleaya cordata* | Papaveraceae | Ranunculales | GCA_002174775.1 | <https://doi.org/10.1016/j.molp.2017.05.007> |
| Funannotate | *Papaver armeniacum* | Papaveraceae | Ranunculales | GCA_023531295.1 | <https://doi.org/10.1038/s41467-022-30856-w> |
| Funannotate | *Papaver atlanticum* | Papaveraceae | Ranunculales | GCA_023531105.1 | <https://doi.org/10.1038/s41467-022-30856-w> |
| Funannotate | *Papaver bracteatum* | Papaveraceae | Ranunculales | GCA_023529315.1 | <https://doi.org/10.1038/s41467-022-30856-w> |
| Funannotate | *Papaver californicum* | Papaveraceae | Ranunculales | GCA_023531435.1 | <https://doi.org/10.1038/s41467-022-30856-w> |
| Funannotate | *Papaver nudicaule* | Papaveraceae | Ranunculales | GCA_023529015.1 | <https://doi.org/10.1038/s41467-022-30856-w> |
| Funannotate | *Papaver somniferum* | Papaveraceae | Ranunculales | GCF_003573695.1 | <https://doi.org/10.1126/science.aat4096> |
| Funannotate | *Stephania cephalantha* | Menispermaceae | Ranunculales | GCA_039657325.1 | <https://doi.org/10.1038/s41467-024-45690-5> |
| Funannotate | *Stephania japonica* | Menispermaceae | Ranunculales | GCA_039657345.1 | <https://doi.org/10.1038/s41467-024-45690-5> |
| Funannotate | *Stephania yunnanensis* | Menispermaceae | Ranunculales | GCA_039657365.1 | <https://doi.org/10.1038/s41467-024-45690-5> |

**Table S4.** QUAST statistics for different genome assembly strategies of *Ranunculus cassubicifolius*. Supernova, and partially SPAdes, MaSuRCA, and Wengan used Illumina short-reads (EH8483/10). Canu, Flye, and Hifiasm, and partially SPAdes, MaSuRCA, and Wengan used ONT (LH040/02) or downsampled PacBio (LH040/06) long-reads. Coverage is given per read type. Polishing included Racon, Medaka / polishCLR, and POLCA steps. N_50_ describes the shortest contig length required to summarize at least half (50%) of the bases of the entire assembly; L_50_ is defined as the minimum number of contigs that encompass half (50%) of the bases of the entire assembly. See Table S5 for comparisons with 25x PacBio data. - = no results were produced.

| **Metric** | **Supernova**  (Illumina22x) | **SPAdes** (Illumina22x) | **SPAdes**  (Illumina22x + ONT16x/ PacBio16x) | **MaSuRCA** (Illumina22x + ONT16x/ PacBio16x) | **Wengan**  (Illumina22x + ONT16x/ PacBio16x) | **Canu**  (ONT16x/ PacBio16x) | **Canu**  (ONT16x/ PacBio16x)  +3x Polish | **Flye** (ONT16x/ PacBio16x) | **Flye** (ONT16x/ PacBio16x)  +3x Polish | **Hifiasm**  (PacBio25x/ PacBio16x) | **Hifiasm**  (PacBio25x)  +3x Polish |
| --- | --- | --- | --- | --- | --- | --- | --- | --- | --- | --- | --- |
| **Total assembly length, Mbp** | 2,76  (86%) | 0,557  (17%) | 0.408/  (13%)/  0.464  (14%) | 0.076/  (2.4%)/  - | 0.005  (0.16%)/  1.21  (38%) | 0.80  (25%)/  5.05  (158%) | 0.81  (25%)/  5.04  (157%) | 3.58  (112%)/  4.90  (153%) | 3.45  (108%)/  4.90  (153%) | 3.22  (101%)/  3.62  (113%) | **3.21**  **(100%)** |
| **No. contigs** | 6,920,808 | 1,231,317 | 1,184,036/  529,574 | 38,650/  - | 368/  6,565,544 | 30,612/  16,262 | 32,959/  15,711 | 65,232/  12,514 | 54,310/  12,356 | 1,453/  3,449 | **1,319** |
| **No. contigs > 1 Mbp** | 562,716 | 122,407 | 110,594/  108,266 | 12,799/  - | 368/  5,046 | 30,612/  16,262 | 31,072/  15,711 | 64,618/  12,506 | 53,572/  12,356 | 1,453/  3,449 | **1,319** |
| **Largest contig, Mbp** | 0.039 | 0.069 | 0.096/  0.112 | 0.145/  - | 0.095/  0.009 | 0.540/  14.3 | 0.542/  14.3 | 0.976/  6.57 | 0.974/  6.57 | 84/  32 | **84** |
| **GC, %** | 40 | 39 | 39/  39 | 40/  - | 40/  39 | 40/  42 | 40/  42 | 43/  42 | 43/  42 | 42/  42 | **42** |
| **N_50_, Mbp** | 0.002 | 0.003 | 0.005/  0.005 | 0.010/  - | 0.021/  0.006 | 0.037/  0.617 | 0.038/  0.618 | 0.103/  0.853 | 0.104/  0.852 | 20.0/  2.89 | **20.0** |
| **L_50_** | 259,069 | 29,900 | 23,910/  23,533 | 1,507/  - | 76/  28,344 | 6,935/  2,374 | 6,937/  2,366 | 10,305/  1728 | 9,889/  1727 | 45/  350 | **45** |

**Table S5.** QUAST statistics for different genome assembly strategies of *Ranunculus cassubicifolius*. Supernova, and partially SPAdes, MaSuRCA, and Wengan used Illumina short-reads (EH8483/10). Canu, Flye, and Hifiasm, and partially SPAdes, MaSuRCA, and Wengan used ONT (LH040/02) or downsampled PacBio (LH040/06) long-reads. Coverage is given per read type. Polishing included Racon, Medaka / polishCLR, and POLCA steps. N_50_ describes the shortest contig length required to summarize at least half (50%) of the bases of the entire assembly; L_50_ is defined as the minimum number of contigs that encompass half (50%) of the bases of the entire assembly. See Table S4 for comparisons with 16x downsampled PacBio data. - = no results were produced.

| **Metric** | **Supernova**  (Illumina22x) | **SPAdes** (Illumina22x) | **SPAdes**  (Illumina22x + ONT16x  /PacBio25x) | **MaSuRCA** (Illumina22x +  ONT16x  /PacBio25x) | **Wengan**  (Illumina22x + ONT16x/  PacBio25x) | **Canu**  (ONT16x  /PacBio25x) | **Canu**  (ONT16x  /PacBio25x)  +3x Polish | **Flye** (ONT16x  /PacBio25x) | **Flye** (ONT16x  /PacBio25x)  +3x Polish | **Hifiasm**  (PacBio25x/ PacBio16x) | **Hifiasm**  (PacBio25x)  +3x Polish |
| --- | --- | --- | --- | --- | --- | --- | --- | --- | --- | --- | --- |
| **Total assembly length, Mbp** | 2,76  (86%) | 0,557  (17%) | 0.408/  (13%)/  0.464  (15%) | 0.076/  (2.4%)/  - | 0.005  (0.16%)/  1.21  (38%) | 0.80  (25%)/  5.12  (160%) | 0.81  (25%)/  5.12  (160%) | 3.58  (112%)/  4.93  (154%) | 3.45  (108%)/  4.92  (154%) | 3.22  (101%)/  3.62  (113%) | **3.21**  **(100%)** |
| **No. contigs** | 6,920,808 | 1,231,317 | 1,184,036/  555,617 | 38,650/  - | 368/  6,565,547 | 30,612/  5,840 | 32,959/  5,208 | 65,232/  8,104 | 54,310/  7,917 | 1,453/  3449 | **1319** |
| **No. contigs > 1 Mbp** | 562,716 | 122,407 | 110,594/  103,406 | 12,799/  - | 368/  5,056 | 30,612/  5,840 | 31,072/  5,208 | 64,618/  8,094 | 53,572/  7,917 | 1,453/  3,449 | **1319** |
| **Largest contig, Mbp** | 0.039 | 0.069 | 0.096/  0.111 | 0.145/  - | 0.095/  0.009 | 0.540/  5.10 | 0.542/  51.6 | 0.976/  12.6 | 0.974/  12.6 | 84/  32 | **84** |
| **GC, %** | 40 | 39 | 39/  39 | 40/  - | 40/  39 | 40/  42 | 40/  42 | 43/  42 | 43/  42 | 42/  42 | **42** |
| **N_50_, Mbp** | 0.002 | 0.003 | 0.005/  0.005 | 0.010/  - | 0.021/  0.006 | 0.037/  3.18 | 0.038/  3.19 | 0.103/  1.73 | 0.104/  1.73 | 20.0/  2.89 | **20.0** |
| **L_50_** | 259,069 | 29,900 | 23,910/  22,261 | 1,507/  - | 76/  28,344 | 6,935/  469 | 6,937/  467 | 10,305/  842 | 9,889/  841 | 45/  350 | **45** |

**Table S6.** BUSCO statistics of nuclear genome sequence assemblies. (a) Short-read Illumina, (b) hybrid-read Illumina + ONT/PacBio, and (c) long-read ONT/PacBio assemblies. Total BUSCO groups searched = 1614. Complete and single-copy BUSCOs (S), complete and duplicated BUSCOs (D), fragmented BUSCOs (F), and missing BUSCOs (M). See Figures 6 and S10 for visualization of BUSCO scores, and further details in the Materials and Methods sections.

**(a)**

|  | **Assemblies** |  |  |  |
| --- | --- | --- | --- | --- |
| **BUSCO scores** | **Supernova_**  **Illumina22x** | **Supernova_**  **Illumina22x_perc** | **SPAdes_**  **Illumina22x** | **SPAdes_**  **Illumina22x_perc** |
| S | 317 | 19.64 | 969 | 60.04 |
| D | 178 | 11.03 | 32 | 1.98 |
| F | 576 | 35.69 | 444 | 27.51 |
| M | 543 | 33.64 | 169 | 10.47 |

**(b)**

|  | **Assemblies** |  |  |  |  |  |
| --- | --- | --- | --- | --- | --- | --- |
| **BUSCO scores** | **SPAdes**  **Illumina22x**  **ONT16x** | **SPAdes**  **Illumina22x**  **ONT16x_perc** | **MaSuRCA**  **Illumina22x**  **ONT16x** | **MaSuRCA**  **Illumina22x**  **ONT16x_perc** | **Wengan**  **Illumina22x**  **ONT16x** | **Wengan**  **Illumina22x**  **ONT16x _perc** |
| S | 1059 | 65.61 | 87 | 5.39 | 3 | 0.19 |
| D | 38 | 2.35 | 2 | 0.12 | 0 | 0.00 |
| F | 393 | 24.35 | 109 | 6.75 | 4 | 0.25 |
| M | 124 | 7.68 | 1416 | 87.73 | 1607 | 99.57 |
|  |  |  |  |  |  |  |
|  | **SPAdes**  **Illumina22x**  **PacBio16x** | **SPAdes**  **Illumina22x**  **PacBio16x_perc** | **MaSuRCA**  **Illumina22x**  **PacBio16x** | **MaSuRCA**  **Illumina22x**  **PacBio16x_perc** | **Wengan**  **Illumina22x**  **PacBio16x** | **Wengan**  **Illumina22x**  **PacBio16x_perc** |
| S | 1082 | 67.04 | **-** | **-** | 16 | 0.99 |
| D | 39 | 2.42 | - | - | 0 | 0.00 |
| F | 372 | 23.05 | - | - | 145 | 8.98 |
| M | 121 | 7.50 | - | - | 1453 | 90.02 |
|  |  |  |  |  |  |  |
|  | **SPAdes**  **Illumina22x**  **PacBio25x** | **SPAdes**  **Illumina22x**  **PacBio25x_perc** | **MaSuRCA**  **Illumina22x**  **PacBio25x** | **MaSuRCA**  **Illumina22x**  **PacBio25x_perc** | **Wengan**  **Illumina22x**  **PacBio25x** | **Wengan**  **Illumina22x**  **PacBio25x_perc** |
| S | 1108 | 68.65 | **-** | **-** | 16 | 0.99 |
| D | 34 | 2.11 | - | - | 0 | 0.00 |
| F | 341 | 21.13 | - | - | 141 | 8.74 |
| M | 131 | 8.12 | - | - | 1457 | 90.27 |

**(c)**

|  | **Assemblies** |  |  |  |  |  |  |  |  |  |  |  |
| --- | --- | --- | --- | --- | --- | --- | --- | --- | --- | --- | --- | --- |
| **BUSCO scores** | **Canu**  **ONT16x** | **Canu**  **ONT16x**  **perc** | **Canu**  **ONT16x**  **Polished** | **Canu**  **ONT16x**  **Polished**  **_perc** | **Flye**  **ONT16x** | **Flye**  **ONT16x**  **perc** | **Flye**  **ONT16x**  **Polished** | **Flye**  **ONT16x**  **Polished**  **_per** |  |  |  |  |
| S | 907 | 56.20 | 1068 | 66.17 | 1052 | 65.17 | 1093 | 67.72 |  |  |  |  |
| D | 127 | 7.87 | 168 | 10.41 | 437 | 27.08 | 475 | 29.43 |  |  |  |  |
| F | 114 | 7.06 | 54 | 3.35 | 79 | 4.89 | 35 | 2.17 |  |  |  |  |
| M | 466 | 28.87 | 324 | 20.07 | 46 | 2.85 | 11 | 0.68 |  |  |  |  |
|  |  |  |  |  |  |  |  |  |  |  |  |  |
|  | **Canu**  **PacBio16x** | **Canu**  **PacBio16x**  **perc** | **Canu**  **PacBio16x**  **Polished** | **Canu**  **PacBio16x**  **Polished**  **perc** | **Flye**  **PacBio16x** | **Flye**  **PacBio16x**  **perc** | **Flye**  **PacBio16x**  **Polished** | **Flye**  **PacBio16x**  **Polished**  **perc** | **Hifiasm**  **Pacbio16x** | **Hifiasm**  **PacBio16x**  **perc** |  |  |
| S | 488 | 30.24 | 403 | 24.97 | 174 | 10.86 | 167 | 10.35 | 909 | 56.32 |  |  |
| D | 1113 | 68.96 | 1199 | 74.29 | 1428 | 88.47 | 1435 | 88.91 | 694 | 43.00 |  |  |
| F | 7 | 0.43 | 7 | 0.43 | 7 | 0.43 | 7 | 0.43 | 7 | 0.43 |  |  |
| M | 6 | 0.37 | 5 | 0.31 | 5 | 0.31 | 5 | 0.31 | 4 | 0.25 |  |  |
|  |  |  |  |  |  |  |  |  |  |  |  |  |
|  | **Canu**  **PacBio25x** | **Canu**  **PacBio25x**  **perc** | **Canu**  **PacBio25x**  **Polished** | **Canu**  **PacBio25x**  **Polished**  **perc** | **Flye**  **PacBio25x** | **Flye**  **PacBio25x**  **perc** | **Flye**  **PacBio25x**  **Polished*** | **Flye**  **PacBio25x**  **Polished**  **Perc*** | **Hifiasm**  **Pacbio25x** | **Hifiasm**  **PacBio25x**  **perc** | **Hifiasm**  **PacBio25x**  **Polished** | **Hifiasm**  **PacBio25x**  **Polished**  **_perc** |
| S | 468 | 29.00 | 354 | 21.93 | 464 | 28.75 | 395 | 24.47 | 1232 | 76.33 | 1217 | 75.40 |
| D | 1138 | 70.51 | 1252 | 77.57 | 1141 | 70.69 | 1211 | 75.03 | 368 | 22.80 | 386 | 23.92 |
| F | 5 | 0.31 | 5 | 0.31 | 6 | 0.37 | 5 | 0.31 | 8 | 0.50 | 4 | 0.25 |
| M | 3 | 0.19 | 3 | 0.19 | 3 | 0.19 | 3 | 0.19 | 6 | 0.37 | 7 | 0.43 |


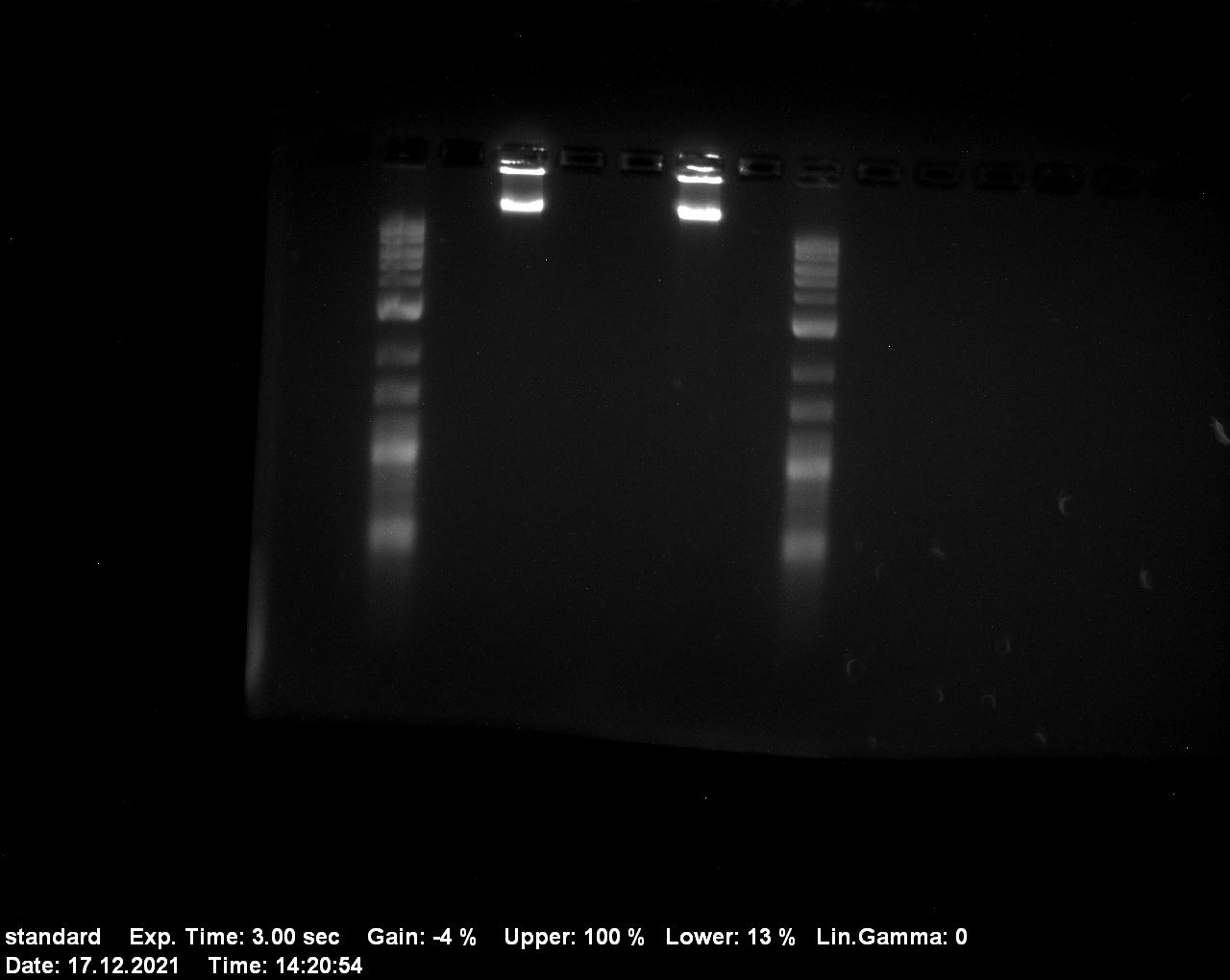


**10 kbp**

**10 kbp**

**Figure S1.** Gel electrophoresis of gDNA extractions from 17^th^ December 2021 using 1 kb DNA Ladder (New England Biolabs, Ipswich, MA, USA; 500 bp to 10 kb) as size standard. The gDNA extract was applied in the central lanes, surrounded on the left and right by the DNA ladder. The top DNA fragment of the ladder is 10 kb. The extracted gDNA is substantially larger than 10 kb, and some very large fragments were not even able to move in the gel. The ONT analyses showed a median fragment length distribution of N_50_ = ca. 23 kb, ranging up to 236 kb.

**Figure S2.** (a-d) Maximum-likelihood phylogeny based on min0 (no filtering), min50, min70, and min90 alignments of 306 plastome sequences of the plant family Ranunculaceae. Filtering was done according to minimum number of samples per alignment site. Transfer expectation values (TBE) are shown. See the Materials & Methods section for more details. The file is attached to the Supplements.

**Figure S3.** (a, b) Maximum-likelihood phylogeny based on 306 plastome sequences of the plant family Ranunculaceae. All branches received full (1) transfer bootstrap expectation (TBE) values, unless otherwise shown. Quartet Sampling (QS) metrics are found in Figure S4. All NCBI species names were checked by gbif.org, and synonyms were replaced by accepted names (synonyms in brackets). The color of clades corresponds to tribe/subfamily names (tribal classification follows Wang et al. (2016) and Zhai et al. (2019), and the plastome of *Ranunculus cassubicifolius* assembled in this study is highlighted with a black arrow. Image sources; Kevin Karbstein, *Anemone sp.*, *Pulsatilla sp.*, *Adonis sp.*, *Nigella sp.*, *Caltha sp.*, *Aquilegia sp.*, and *Trollius sp.*; inaturalist.org (CC-BY-NC), *Clematis* *sp.* (photos/344038634), *Aconitum* *sp.* (observations/175339012), *Actaea sp.* (observations/154486471)*,* and *Coptis sp.* (observations/152014320). See Texts S2 and S3 for more details. The file is attached to the Supplements.

**Figure S4**. Maximum-likelihood phylogeny based on 306 plastome sequences and the min90 alignment of the plant family Ranunculaceae. See Pease et al. (2018) and Karbstein, Tomasello et al. (2020) for interpretation of QS metrics, and the Materials and Methods section for more details. The file is attached to the Supplements.

**(a)**

***Ranunculus cassubicifolius***

*Aconitum carmichaelii*

*Pulsatilla dahurica*

*Pulsatilla kissii*

*Pulsatilla cernua*

*Paropyrum anemonoides*

*Corydalis pauciovulata*

*Anemone maxima*

*Aconitum kusnezoffii*

**(b)**

Illumina-ONT

***Ranunculus cassubicifolius***

Illumina-PacBio

***Ranunculus cassubicifolius***

**Figure S5.** Whole genome alignment analysis of (a) all available mitogenome sequences in Ranunculaceae, and (b) of the assembled Illumina-ONT and -PacBio genome sequences of *Ranunculus cassubicifolius* (LH040). We performed a progressiveMAUVE analysis (2015-02-25; Darling et al., 2010). Shown are locally collinear blocks, i.e., homologous regions of sequences shared by the genomes under study, without any rearrangements of homologous sequences. The figures show substantial rearrangements of collinear blocks (a) among species but also (b) between individuals of the same species. See the Materials and Methods section for more details.


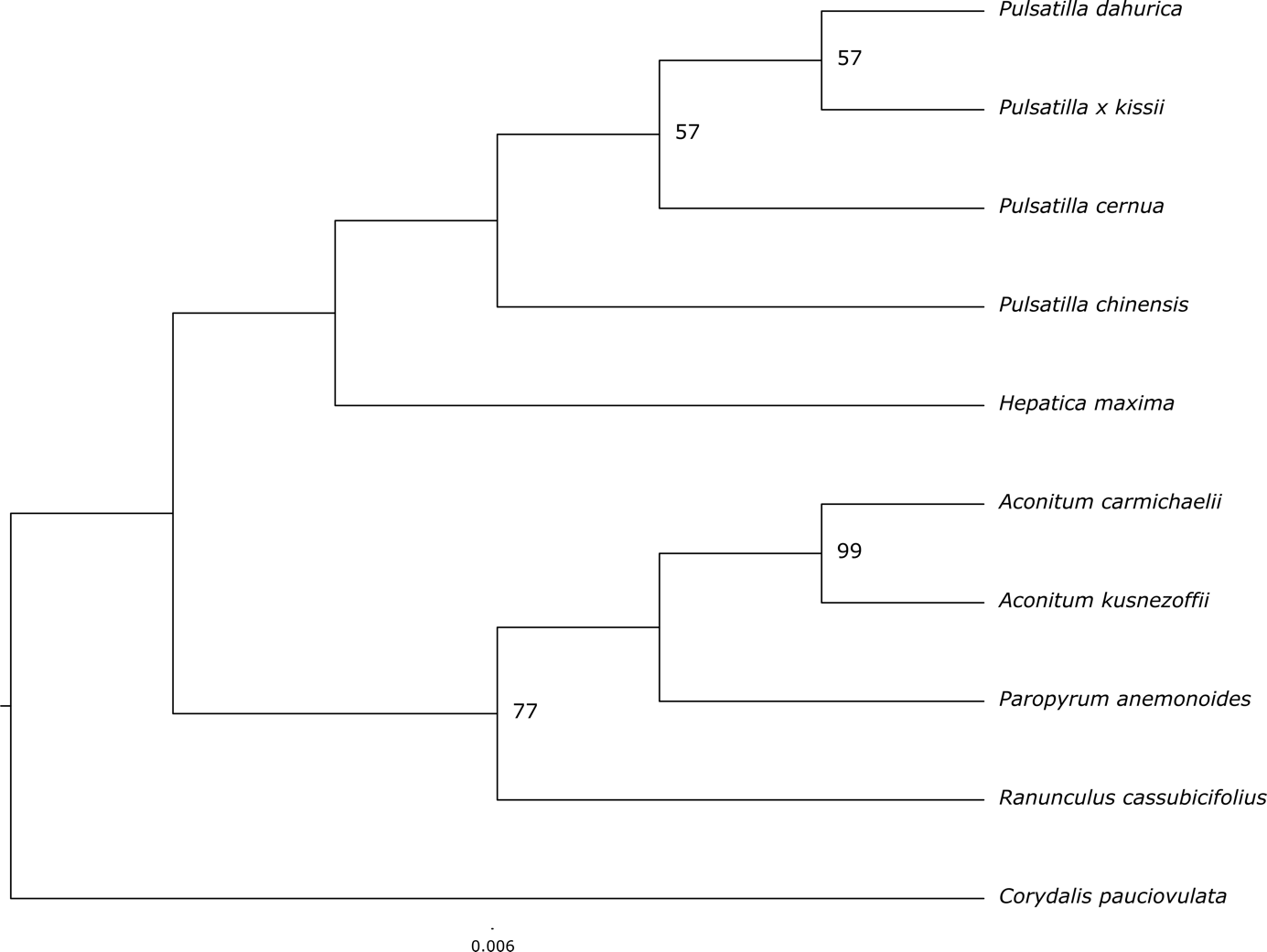


**Figure S6.** Concatenation-based phylogeny of 10 mitogenome sequences and 42 genes of Ranunculaceae (see Figure 3b for the coalescent-based phylogeny). Missing sites were not removed, and the TIM1+I+G4 model was chosen by ModelTest-NG. All branches received full (100%) Felsenstein Bootstrap Proportions (FBP) values, unless otherwise shown.

**Figure S7.** Hi-C contact map. Shown is a heatmap representation of chromatin interactions, derived from the Hi-C data. Please see the Material & Methods for more details. The diagonal represents the regions that are close to each other. The color scale indicates the frequency of chromatin interactions (red areas likely indicate high contact frequencies, meaning those regions of the genome are physically close in the 3D space of the nucleus. Blue rectangles along the diagonal highlight topologically associating domains with high intrachromatin contact, i.e., the 8 pseudochromosomes of *Ranunculus cassubicifolius*. The file is attached to the Supplements.

**Figure S8.** (a-h) ModDotPlots of pseudochromosomes 1-8 of the final PacBio genome assembly (Table 1, ‘Nuclear Genome’). These plots are able to visualize multi-megabase tandem repeat arrays within centromeres (as clusters of high similarity). Blue = low genetic identity, yellow = high genetic identity. We classified four chromosomes (Chr1, Chr2, Chr5, and Chr6) as metacentric and four as submetacentric-subtelocentric (Chr3, Chr4, Chr7, and Chr8). A detailed morphological karyotype of the species is given in Hörandl et al. (1997). The file is attached to the Supplements.

**Figure S9.** Detection of ancient whole genome duplication (WGD) events in *Ranunculus cassubicifolius*. (a) Corrected substitution rate (K_S_) with ELMM mixture modeling of duplicated genes and anchor pairs of *R. cassubicifolius* (grey and green bars, respectively) in relation to six available and gene-annotated high-quality genomes of Ranunculales (different colors) produced by wgd2 pipeline (Chen and Zwaenepoel, 2023; Chen et al., 2024). The analysis is based on Multiplicon Homologous Regions (MHR), which summarize homologous regions across the entire genome that likely represent duplicated blocks retained after a WGD event. These regions are constructed from anchor pairs found in syntenic regions. In *R. cassubicifolius*, anchor K_S_ distribution of paralogous genes indicates a more recent (left peak) and a more ancient (right peak) duplication (see also Figure 5a). In interspecies comparisons (GlobalMHR), the K_S_ anchor pair peak of *R. cassubicifolius* (1.63) occurs after all other Ranunculaceae species (1.1–1.2) except *Stephania* (Menispermaceae: 1.66). This suggests that the WGD event likely occurred in the common ancestor of *Ranunculus*, *Aquilegia*, *Coptis*, and *Thalictrum* at the base of Ranunculaceae. K_S_ = synonymous substitutions per site of duplicated genes; anchor pairs = pairs of duplicates from a WGD event. (b) Interspecies ‘syndepth’ plots based on orthogroups show collinear block ratios per species pair, and support previous interspecies K_S_ results. A syndepth plot is used in comparative genomics to show the depth of synteny between two genomes, that is, how many orthologous or paralogous genomic segments in one species align with each segment in another species. Most examined species show a collinear block ratio of 0:0, likely due to lineage-specific evolution and the presence of many genes that do not belong to any collinear block or are not found in the same block within a species pairs. Interestingly, *Stephania* shows no triplicated genes in orthogroup comparisons. This may suggest that the WGD event occurred at the base of Ranunculaceae rather than at an earlier stage. The high collinear block ratios of *Papaver* are related to a recent lineage-specific WGD event. See the Materials & Methods section, and item [04] on FigShare including all wgd2 results for more details. The files are attached to the Supplements.

**Figure S10.** BUSCO assessments for different genome assembly strategies of the diploid sexual species *Ranunculus cassubicifolius*. Supernova, and partially SPAdes, MaSuRCA, and Wengan used Illumina short-reads (EH8483/10). Canu, Flye, and Hifiasm, and partially SPAdes, MaSuRCA, and Wengan used ONT (LH040/02) or PacBio (LH040/06) long-reads. The polishing of the Canu, Flye, and Hifiasm assemblies includes the following steps: polishing via filtered ONT reads using Racon and Medaka / via filtered PacBio reads using Racon and polishCLR, respectively, and via filtered Illumina reads using POLCA (MaSuRCA pipeline). The best assembly in terms of complete BUSCO genes (S+D) is highlighted with a black square, that is Hifiasm (PacBio) + Polishing. Analyses are based on 1,614 BUSCO genes. See the Materials and Methods section, and Tables S4 and S5 for more details. See Figure 6 for comparisons with downsampled (16x) PacBio reads. The file is attached to the Supplements.

**References**

Bolger, A. M., Lohse, M., and Usadel, B. (2014). Trimmomatic: a flexible trimmer for Illumina sequence data. *Bioinformatics* 30, 2114–2120. doi: 10.1093/bioinformatics/btu170

Bray, N. L., Pimentel, H., Melsted, P., and Pachter, L. (2016). Near-optimal probabilistic RNA-seq quantification. *Nat Biotechnol* 34, 525–527. doi: 10.1038/nbt.3519

Brůna, T., Lomsadze, A., and Borodovsky, M. (2024). GeneMark-ETP significantly improves the accuracy of automatic annotation of large eukaryotic genomes. *Genome Res* 34, 757–768. doi: 10.1101/gr.278373.123

Cantalapiedra, C. P., Hernández-Plaza, A., Letunic, I., Bork, P., and Huerta-Cepas, J. (2021). eggNOG-mapper v2: functional annotation, orthology assignments, and domain prediction at the metagenomic scale. *Mol Biol Evol* 38, 5825–5829. doi: 10.1093/molbev/msab293

Chen, H., and Zwaenepoel, A. (2023). “Inference of ancient polyploidy from genomic data,” in *Polyploidy: Methods and Protocols*, ed. Y. Van de Peer (New York, NY: Springer US), 3–18. doi: 10.1007/978-1-0716-2561-3_1

Chen, H., Zwaenepoel, A., and Van de Peer, Y. (2024). wgd v2: a suite of tools to uncover and date ancient polyploidy and whole-genome duplication. *Bioinformatics* 40. doi: 10.1093/bioinformatics/btae272

Darling, A. E., Mau, B., and Perna, N. T. (2010). progressiveMauve: multiple genome alignment with gene gain, loss and rearrangement. *PLoS One* 5, e11147. doi: 10.1371/journal.pone.0011147

Doyle, J. J., and Dickson, E. E. (1987). Preservation of plant samples for DNA restriction endonuclease analysis. *Taxon* 36, 715–722. doi: 10.2307/1221122

Eaton, D. A. R., Spriggs, E. L., Park, B., and Donoghue, M. J. (2017). Misconceptions on missing data in RAD-seq phylogenetics with a deep-scale example from flowering plants. *Syst Biol* 66, 399–412. doi: 10.1093/sysbio/syw092

Frith, M. C. (2011). A new repeat-masking method enables specific detection of homologous sequences. *Nucleic Acids Res* 39, e23–e23. doi: 10.1093/nar/gkq1212

Gabriel, L., Brůna, T., Hoff, K. J., Ebel, M., Lomsadze, A., Borodovsky, M., et al. (2024). BRAKER3: Fully automated genome annotation using RNA-seq and protein evidence with GeneMark-ETP, AUGUSTUS, and TSEBRA. *Genome Res* 34, 769–777. doi: 10.1101/gr.278090.123

Grabherr, M. G., Haas, B. J., Yassour, M., Levin, J. Z., Thompson, D. A., Amit, I., et al. (2011). Full-length transcriptome assembly from RNA-Seq data without a reference genome. *Nat Biotechnol* 29, 644–652. doi: 10.1038/nbt.1883

Haas, B. J., Salzberg, S. L., Zhu, W., Pertea, M., Allen, J. E., Orvis, J., et al. (2008). Automated eukaryotic gene structure annotation using EVidenceModeler and the program to assemble spliced alignments. *Genome Biol* 9, R7. doi: 10.1186/gb-2008-9-1-r7

Hakim, S. E., Choudhary, N., Malhotra, K., Peng, J., Arafa, A., Bültemeier, A., et al. (2024). Phylogenomics and metabolic engineering reveal a conserved gene cluster in Solanaceae plants for withanolide biosynthesis. *bioRxiv*, 1–44. doi: 10.1101/2024.09.27.614867

Hörandl, E., Dobes, C., Lambrou, M. 1997. Chromosome and pollen studies on Austrian species of the apomictic *Ranunculus auricomus* complex. *Bot Helv* 207, 195–209.

Horz, J. M., Wolff, K., Friedhoff, R., and Pucker, B. (2024). Genome sequence of the medicinal and ornamental plant *Digitalis purpurea* reveals the molecular basis of flower color variation. *bioRxiv*, 1–27.

Jones, P., Binns, D., Chang, H.-Y., Fraser, M., Li, W., McAnulla, C., et al. (2014). InterProScan 5: genome-scale protein function classification. *Bioinformatics* 30, 1236–1240. doi: 10.1093/bioinformatics/btu031

Karbstein, K., Tomasello, S., Hodač, L., Dunkel, F. G., Daubert, M., and Hörandl, E. (2020). Phylogenomics supported by geometric morphometrics reveals delimitation of sexual species within the polyploid apomictic *Ranunculus auricomus* complex (Ranunculaceae). *Taxon* 69, 1191–1220. doi: 10.1002/tax.12365

Keilwagen, J., Hartung, F., Paulini, M., Twardziok, S. O., and Grau, J. (2018). Combining RNA-seq data and homology-based gene prediction for plants, animals and fungi. *BMC Bioinformatics* 19, 189. doi: 10.1186/s12859-018-2203-5

Keilwagen, J., Wenk, M., Erickson, J. L., Schattat, M. H., Grau, J., and Hartung, F. (2016). Using intron position conservation for homology-based gene prediction. *Nucleic Acids Res* 44, e89–e89. doi: 10.1093/nar/gkw092

Kim, D., Paggi, J. M., Park, C., Bennett, C., and Salzberg, S. L. (2019). Graph-based genome alignment and genotyping with HISAT2 and HISAT-genotype. *Nat Biotechnol* 37, 907–915. doi: 10.1038/s41587-019-0201-4

Korf, I. (2004). Gene finding in novel genomes. *BMC Bioinformatics* 5, 1–9. doi: 10.1186/1471-2105-5-59

Kuznetsov, D., Tegenfeldt, F., Manni, M., Seppey, M., Berkeley, M., Kriventseva, E. V, et al. (2023). OrthoDB v11: annotation of orthologs in the widest sampling of organismal diversity. *Nucleic Acids Res* 51, D445–D451. doi: 10.1093/nar/gkac998

Li, Z., Parris, S., and Saski, C. A. (2020). A simple plant high-molecular-weight DNA extraction method suitable for single-molecule technologies. *Plant Methods* 16, 38. doi: 10.1186/s13007-020-00579-4

Majoros, W. H., Pertea, M., and Salzberg, S. L. (2004). TigrScan and GlimmerHMM: two open source ab initio eukaryotic gene-finders. *Bioinformatics* 20, 2878–2879. doi: 10.1093/bioinformatics/bth315

Nowak, M. S., Harder, B., Meckoni, S. N., Friedhoff, R., Wolff, K., and Pucker, B. (2024). Genome sequence and RNA-seq analysis reveal genetic basis of flower coloration in the giant water lily *Victoria cruziana*. *bioRxiv*, 1–23. doi: 10.1101/2024.06.15.599162

Ou, S., and Jiang, N. (2018). LTR_retriever: A highly accurate and sensitive program for identification of long terminal repeat retrotransposons. *Plant Physiol* 176, 1410–1422. doi: 10.1104/pp.17.01310

Ou, S., and Jiang, N. (2019). LTR_FINDER_parallel: parallelization of LTR_FINDER enabling rapid identification of long terminal repeat retrotransposons. *Mob DNA* 10, 48. doi: 10.1186/s13100-019-0193-0

Paetzold, C., Barke, B. H., and Hörandl, E. (2022). Evolution of transcriptomes in early-generation hybrids of the apomictic *Ranunculus auricomus complex* (Ranunculaceae). *Int J Mol Sci* 23, 13881. doi: 10.3390/ijms232213881

Palmer, J. M., and Stajich, J. E. (2022). Funannotate (Version 1.8.15) [Computer software].

Pease, J. B., Brown, J. W., Walker, J. F., Hinchliff, C. E., and Smith, S. A. (2018). Quartet Sampling distinguishes lack of support from conflicting support in the green plant tree of life. *Am J Bot* 105, 385–403. doi: 10.1002/ajb2.1016

Russo, A., Mayjonade, B., Frei, D., Potente, G., Kellenberger, R. T., Frachon, L., et al. (2022). Low-input high-molecular-weight DNA extraction for long-read sequencing from plants of diverse families. *Front Plant Sci* 13, 1–12. doi: 10.3389/fpls.2022.883897

Shi, J., and Liang, C. (2019). Generic Repeat Finder: A high-sensitivity tool for genome-wide de novo repeat detection. *Plant Physiol* 180, 1803–1815. doi: 10.1104/pp.19.00386

Stanke, M., Diekhans, M., Baertsch, R., and Haussler, D. (2008). Using native and syntenically mapped cDNA alignments to improve de novo gene finding. *Bioinformatics* 24, 637–644. doi: 10.1093/bioinformatics/btn013

Stanke, M., Schöffmann, O., Morgenstern, B., and Waack, S. (2006). Gene prediction in eukaryotes with a generalized hidden Markov model that uses hints from external sources. *BMC Bioinformatics* 7, 62. doi: 10.1186/1471-2105-7-62

Steinegger, M., and Söding, J. (2017). MMseqs2 enables sensitive protein sequence searching for the analysis of massive data sets. *Nat Biotechnol* 35, 1026–1028. doi: 10.1038/nbt.3988

Su, W., Gu, X., and Peterson, T. (2019). TIR-Learner, a new ensemble method for TIR transposable element annotation, provides evidence for abundant new transposable elements in the maize genome. *Mol Plant* 12, 447–460. doi: 10.1016/j.molp.2019.02.008

Vaillancourt, B., and Buell, C. R. (2019). High molecular weight DNA isolation method from diverse plant species for use with Oxford Nanopore sequencing. *bioRxiv*, 1–13. doi: 10.1101/783159

Wang, W., Lin, L., Xiang, X.-G., Ortiz, R. D. C., Liu, Y., Xiang, K.-L., et al. (2016). The rise of angiosperm-dominated herbaceous floras: Insights from Ranunculaceae. *Sci Rep* 6, 27259. doi: 10.1038/srep27259

Wang, W., Lu, A.-M., Ren, Y., Endress, M. E., and Chen, Z.-D. (2009). Phylogeny and classification of Ranunculales: Evidence from four molecular loci and morphological data. *Perspect Plant Ecol Evol Syst* 11, 81–110. doi: 10.1016/j.ppees.2009.01.001

Xiong, W., He, L., Lai, J., Dooner, H. K., and Du, C. (2014). HelitronScanner uncovers a large overlooked cache of Helitron transposons in many plant genomes. *Proceedings of the National Academy of Sciences* 111, 10263–10268. doi: 10.1073/pnas.1410068111

Xu, Z., and Wang, H. (2007). LTR_FINDER: an efficient tool for the prediction of full-length LTR retrotransposons. *Nucleic Acids Res* 35, W265–W268. doi: 10.1093/nar/gkm286

Zhai, W., Duan, X., Zhang, R., Guo, C., Li, L., Xu, G., et al. (2019). Chloroplast genomic data provide new and robust insights into the phylogeny and evolution of the Ranunculaceae. *Mol Phylogenet Evol* 135, 12–21. doi: 10.1016/j.ympev.2019.02.024

Zhang, R.-G., Li, G.-Y., Wang, X.-L., Dainat, J., Wang, Z.-X., Ou, S., et al. (2022). TEsorter: An accurate and fast method to classify LTR-retrotransposons in plant genomes. *Hortic Res* 9, 0–3. doi: 10.1093/hr/uhac017
