# Supplementary figures and images for: Assembling genomes of non‐model plants: A case study with evolutionary insights from Ranunculus (Ranunculaceae)
Source: Plant J. 2025 Sep 19;123(6):e70390. doi: 10.1111/tpj.70390 (PMC12448783; doi:10.1111/tpj.70390)

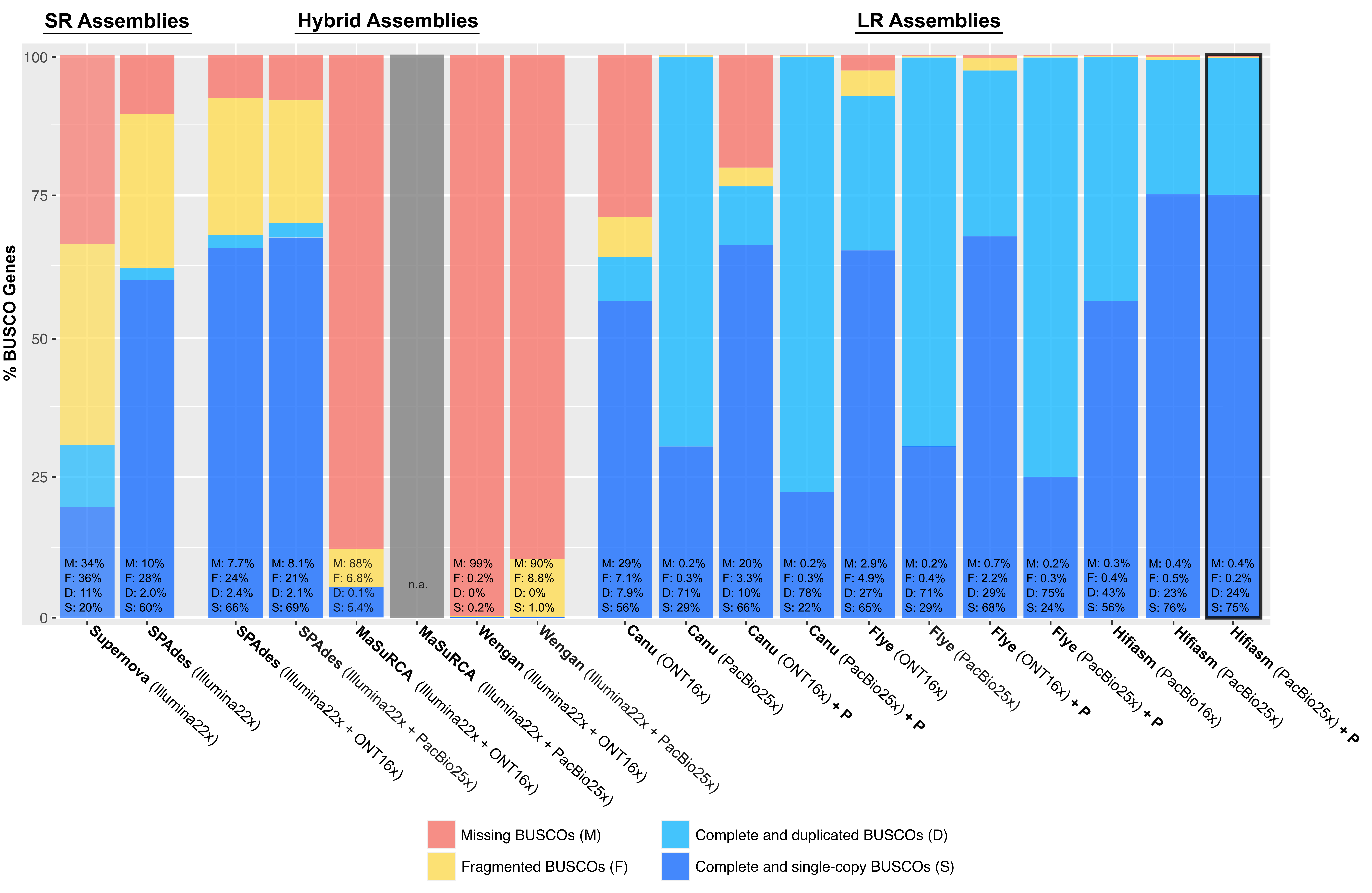

Supplement: Supplementary file 1 — Text S1. (a, b) Extraction of genomic DNA (gDNA) and (b) library preparation for Oxford Nanopore Technology (ONT) sequencing performed at the University of Göttingen. Text S2. Determining the optimal DNA sequence alignment for phylogenetic analyses. Text S3. Identification of tandem repeats (TRs) and transposable elements (TEs), and protein‐coding genes. Text S4. Detailed results of plastome‐based phylogenies in Ranunculaceae. Text S5. Impact of using frozen libraries for ONT DNA sequencing. Figure S1. Gel electrophoresis of gDNA extractions from 17th December 2021 using 1 kb DNA Ladder (New England Biolabs, Ipswich, MA, USA; 500 bp–10 kb) as size standard. Figure S2. (a–d) Maximum‐likelihood phylogeny based on min0 (no filtering), min50, min70, and min90 alignments of 306 plastomes (taxa) of the plant family Ranunculaceae. Figure S3. (a, b) Maximum‐likelihood phylogeny based on 306 plastomes (292 taxa) of the plant family Ranunculaceae. Figure S4. Maximum‐likelihood phylogeny based on 306 plastome sequences (292 taxa) and the min90 alignment of the plant family Ranunculaceae. Figure S5. Whole genome alignment analysis of (a) all available mitogenome sequences in Ranunculaceae, and (b) of the assembled Illumina‐ONT and ‐PacBio genome sequences of Ranunculus cassubicifolius (LH040). Figure S6. Concatenation‐based phylogeny of 10 mitogenome sequences and 42 genes of Ranunculaceae (see Figure 3b for the coalescent‐based phylogeny). Figure S7. Hi‐C contact map. Figure S8. (a–h) ModDotPlots of pseudochromosomes 1–8 of the final PacBio genome assembly (Table 1, ‘Nuclear Genome’). Figure S9. Detection of ancient whole genome duplication (WGD) events in Ranunculus cassubicifolius. Figure S10. BUSCO assessments (PacBio 25×) for different genome assembly strategies of the diploid sexual species Ranunculus cassubicifolius. Table S1. Selected (a) plastome and (b) mitogenome sequences from NCBI. Table S2. RNA‐seq data of 37 Ranunculaceae individuals from SRA/NCBI used for Ranunc [file TPJ-123-0-s001.zip › tpj70390-sup-0008-FigureS10.png]

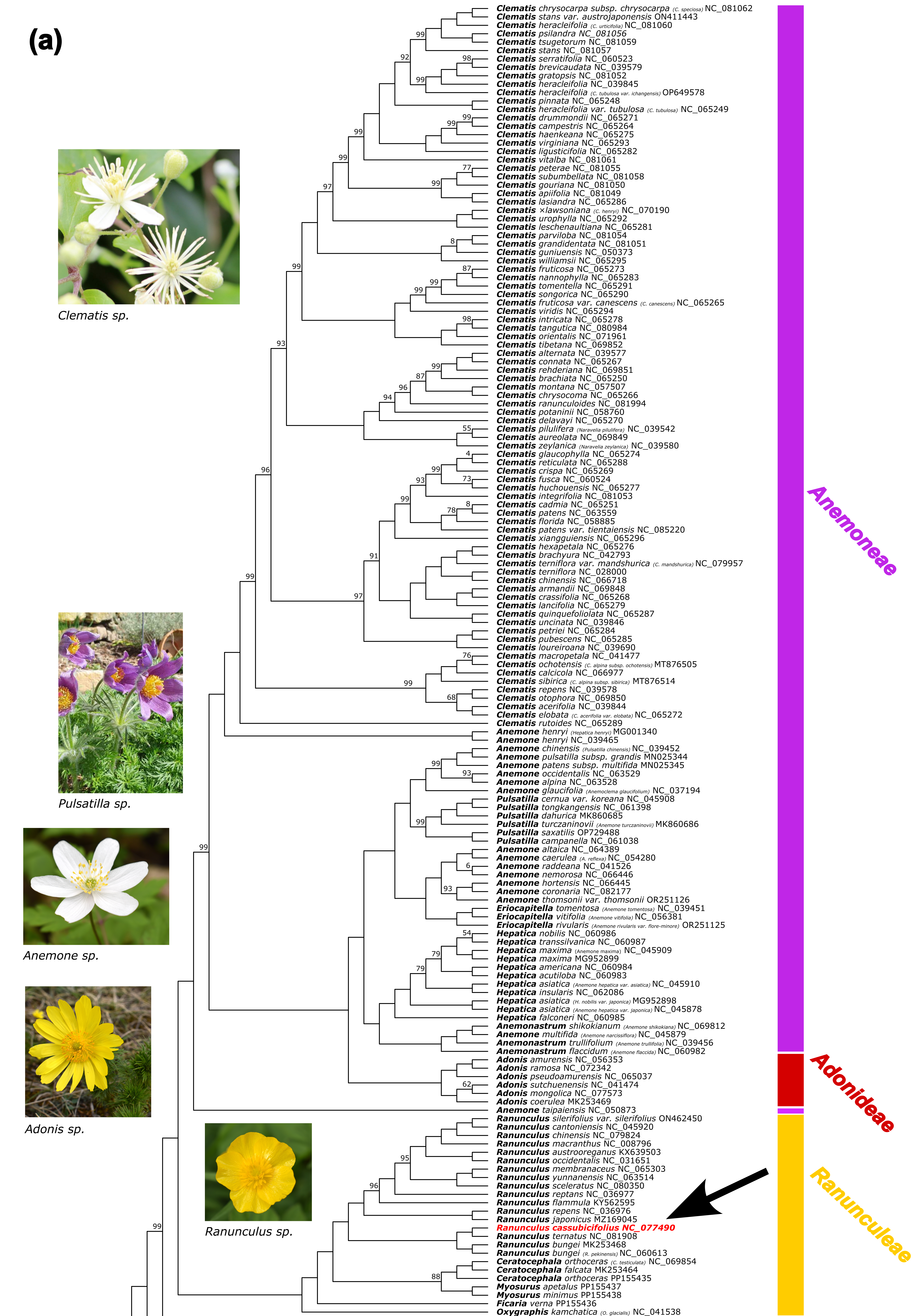

Supplement: Supplementary file 1 — Text S1. (a, b) Extraction of genomic DNA (gDNA) and (b) library preparation for Oxford Nanopore Technology (ONT) sequencing performed at the University of Göttingen. Text S2. Determining the optimal DNA sequence alignment for phylogenetic analyses. Text S3. Identification of tandem repeats (TRs) and transposable elements (TEs), and protein‐coding genes. Text S4. Detailed results of plastome‐based phylogenies in Ranunculaceae. Text S5. Impact of using frozen libraries for ONT DNA sequencing. Figure S1. Gel electrophoresis of gDNA extractions from 17th December 2021 using 1 kb DNA Ladder (New England Biolabs, Ipswich, MA, USA; 500 bp–10 kb) as size standard. Figure S2. (a–d) Maximum‐likelihood phylogeny based on min0 (no filtering), min50, min70, and min90 alignments of 306 plastomes (taxa) of the plant family Ranunculaceae. Figure S3. (a, b) Maximum‐likelihood phylogeny based on 306 plastomes (292 taxa) of the plant family Ranunculaceae. Figure S4. Maximum‐likelihood phylogeny based on 306 plastome sequences (292 taxa) and the min90 alignment of the plant family Ranunculaceae. Figure S5. Whole genome alignment analysis of (a) all available mitogenome sequences in Ranunculaceae, and (b) of the assembled Illumina‐ONT and ‐PacBio genome sequences of Ranunculus cassubicifolius (LH040). Figure S6. Concatenation‐based phylogeny of 10 mitogenome sequences and 42 genes of Ranunculaceae (see Figure 3b for the coalescent‐based phylogeny). Figure S7. Hi‐C contact map. Figure S8. (a–h) ModDotPlots of pseudochromosomes 1–8 of the final PacBio genome assembly (Table 1, ‘Nuclear Genome’). Figure S9. Detection of ancient whole genome duplication (WGD) events in Ranunculus cassubicifolius. Figure S10. BUSCO assessments (PacBio 25×) for different genome assembly strategies of the diploid sexual species Ranunculus cassubicifolius. Table S1. Selected (a) plastome and (b) mitogenome sequences from NCBI. Table S2. RNA‐seq data of 37 Ranunculaceae individuals from SRA/NCBI used for Ranunc [file TPJ-123-0-s001.zip › tpj70390-sup-0003-FigureS3/Figure_S3a_min90_pt_phylogeny_ranunculaceae.png]

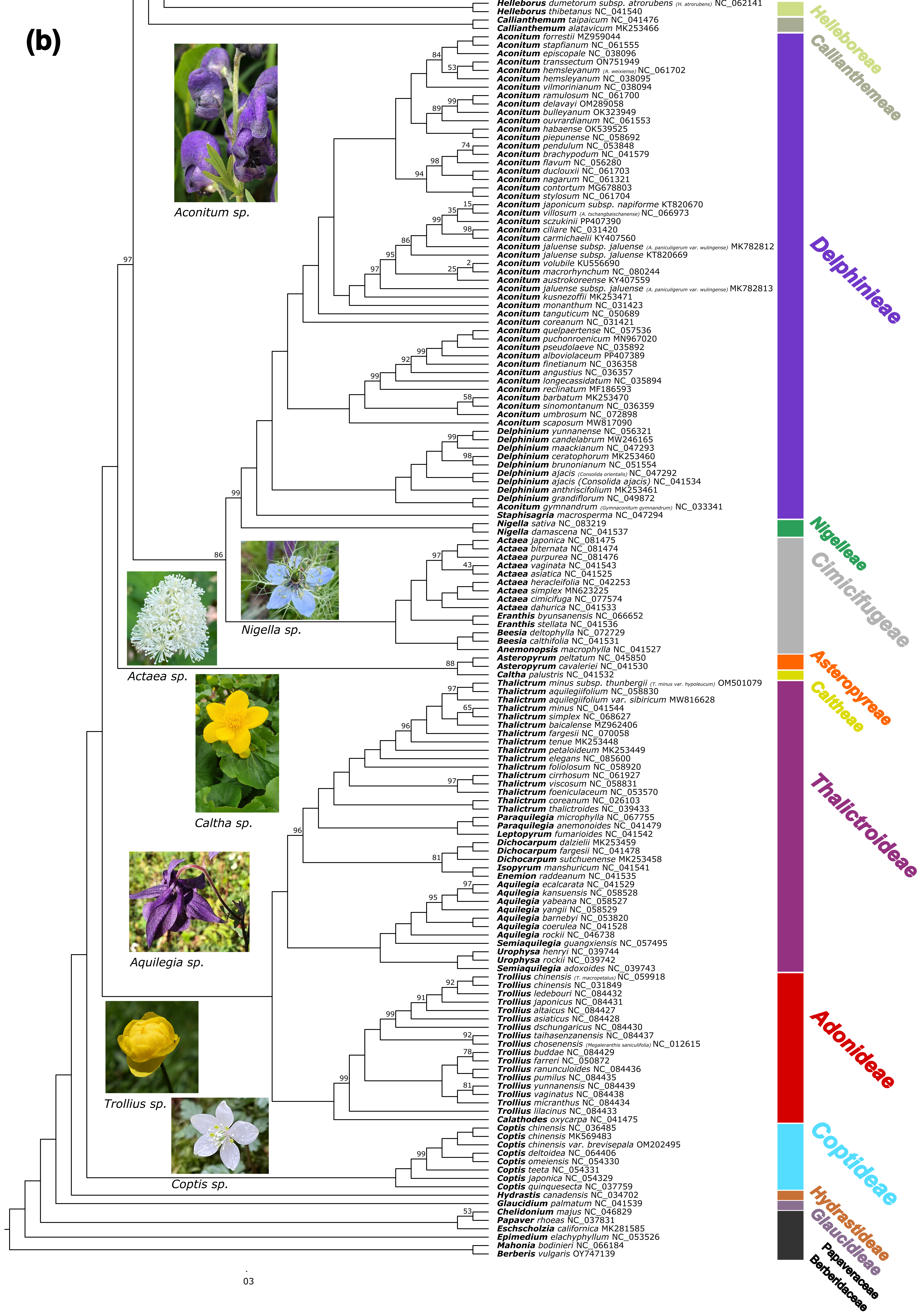

Supplement: Supplementary file 1 — Text S1. (a, b) Extraction of genomic DNA (gDNA) and (b) library preparation for Oxford Nanopore Technology (ONT) sequencing performed at the University of Göttingen. Text S2. Determining the optimal DNA sequence alignment for phylogenetic analyses. Text S3. Identification of tandem repeats (TRs) and transposable elements (TEs), and protein‐coding genes. Text S4. Detailed results of plastome‐based phylogenies in Ranunculaceae. Text S5. Impact of using frozen libraries for ONT DNA sequencing. Figure S1. Gel electrophoresis of gDNA extractions from 17th December 2021 using 1 kb DNA Ladder (New England Biolabs, Ipswich, MA, USA; 500 bp–10 kb) as size standard. Figure S2. (a–d) Maximum‐likelihood phylogeny based on min0 (no filtering), min50, min70, and min90 alignments of 306 plastomes (taxa) of the plant family Ranunculaceae. Figure S3. (a, b) Maximum‐likelihood phylogeny based on 306 plastomes (292 taxa) of the plant family Ranunculaceae. Figure S4. Maximum‐likelihood phylogeny based on 306 plastome sequences (292 taxa) and the min90 alignment of the plant family Ranunculaceae. Figure S5. Whole genome alignment analysis of (a) all available mitogenome sequences in Ranunculaceae, and (b) of the assembled Illumina‐ONT and ‐PacBio genome sequences of Ranunculus cassubicifolius (LH040). Figure S6. Concatenation‐based phylogeny of 10 mitogenome sequences and 42 genes of Ranunculaceae (see Figure 3b for the coalescent‐based phylogeny). Figure S7. Hi‐C contact map. Figure S8. (a–h) ModDotPlots of pseudochromosomes 1–8 of the final PacBio genome assembly (Table 1, ‘Nuclear Genome’). Figure S9. Detection of ancient whole genome duplication (WGD) events in Ranunculus cassubicifolius. Figure S10. BUSCO assessments (PacBio 25×) for different genome assembly strategies of the diploid sexual species Ranunculus cassubicifolius. Table S1. Selected (a) plastome and (b) mitogenome sequences from NCBI. Table S2. RNA‐seq data of 37 Ranunculaceae individuals from SRA/NCBI used for Ranunc [file TPJ-123-0-s001.zip › tpj70390-sup-0003-FigureS3/Figure_S3b_min90_pt_phylogeny_ranunculaceae.png]

./Chr1

Chr1

0.4

0.3

0.2

0.1

0.0

0.0

0.1

0.2

0.3

0.4

Chr1

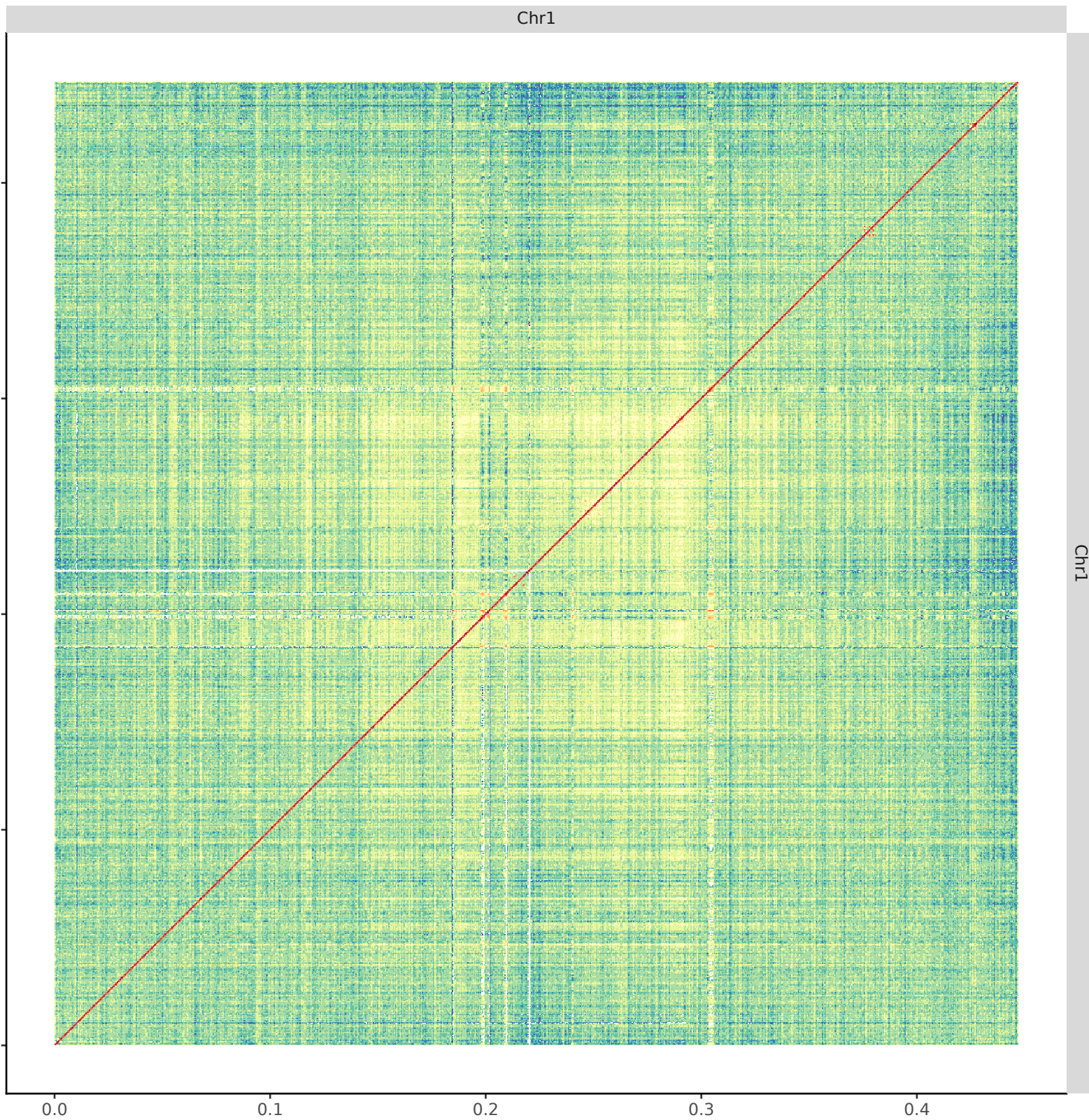

Supplement: Supplementary file 1 — Text S1. (a, b) Extraction of genomic DNA (gDNA) and (b) library preparation for Oxford Nanopore Technology (ONT) sequencing performed at the University of Göttingen. Text S2. Determining the optimal DNA sequence alignment for phylogenetic analyses. Text S3. Identification of tandem repeats (TRs) and transposable elements (TEs), and protein‐coding genes. Text S4. Detailed results of plastome‐based phylogenies in Ranunculaceae. Text S5. Impact of using frozen libraries for ONT DNA sequencing. Figure S1. Gel electrophoresis of gDNA extractions from 17th December 2021 using 1 kb DNA Ladder (New England Biolabs, Ipswich, MA, USA; 500 bp–10 kb) as size standard. Figure S2. (a–d) Maximum‐likelihood phylogeny based on min0 (no filtering), min50, min70, and min90 alignments of 306 plastomes (taxa) of the plant family Ranunculaceae. Figure S3. (a, b) Maximum‐likelihood phylogeny based on 306 plastomes (292 taxa) of the plant family Ranunculaceae. Figure S4. Maximum‐likelihood phylogeny based on 306 plastome sequences (292 taxa) and the min90 alignment of the plant family Ranunculaceae. Figure S5. Whole genome alignment analysis of (a) all available mitogenome sequences in Ranunculaceae, and (b) of the assembled Illumina‐ONT and ‐PacBio genome sequences of Ranunculus cassubicifolius (LH040). Figure S6. Concatenation‐based phylogeny of 10 mitogenome sequences and 42 genes of Ranunculaceae (see Figure 3b for the coalescent‐based phylogeny). Figure S7. Hi‐C contact map. Figure S8. (a–h) ModDotPlots of pseudochromosomes 1–8 of the final PacBio genome assembly (Table 1, ‘Nuclear Genome’). Figure S9. Detection of ancient whole genome duplication (WGD) events in Ranunculus cassubicifolius. Figure S10. BUSCO assessments (PacBio 25×) for different genome assembly strategies of the diploid sexual species Ranunculus cassubicifolius. Table S1. Selected (a) plastome and (b) mitogenome sequences from NCBI. Table S2. RNA‐seq data of 37 Ranunculaceae individuals from SRA/NCBI used for Ranunc [file TPJ-123-0-s001.zip › tpj70390-sup-0006-FigureS8/Figure_S8_ah_ModDotPlots_PDFs/Chr1_FULL.pdf]

./Chr2

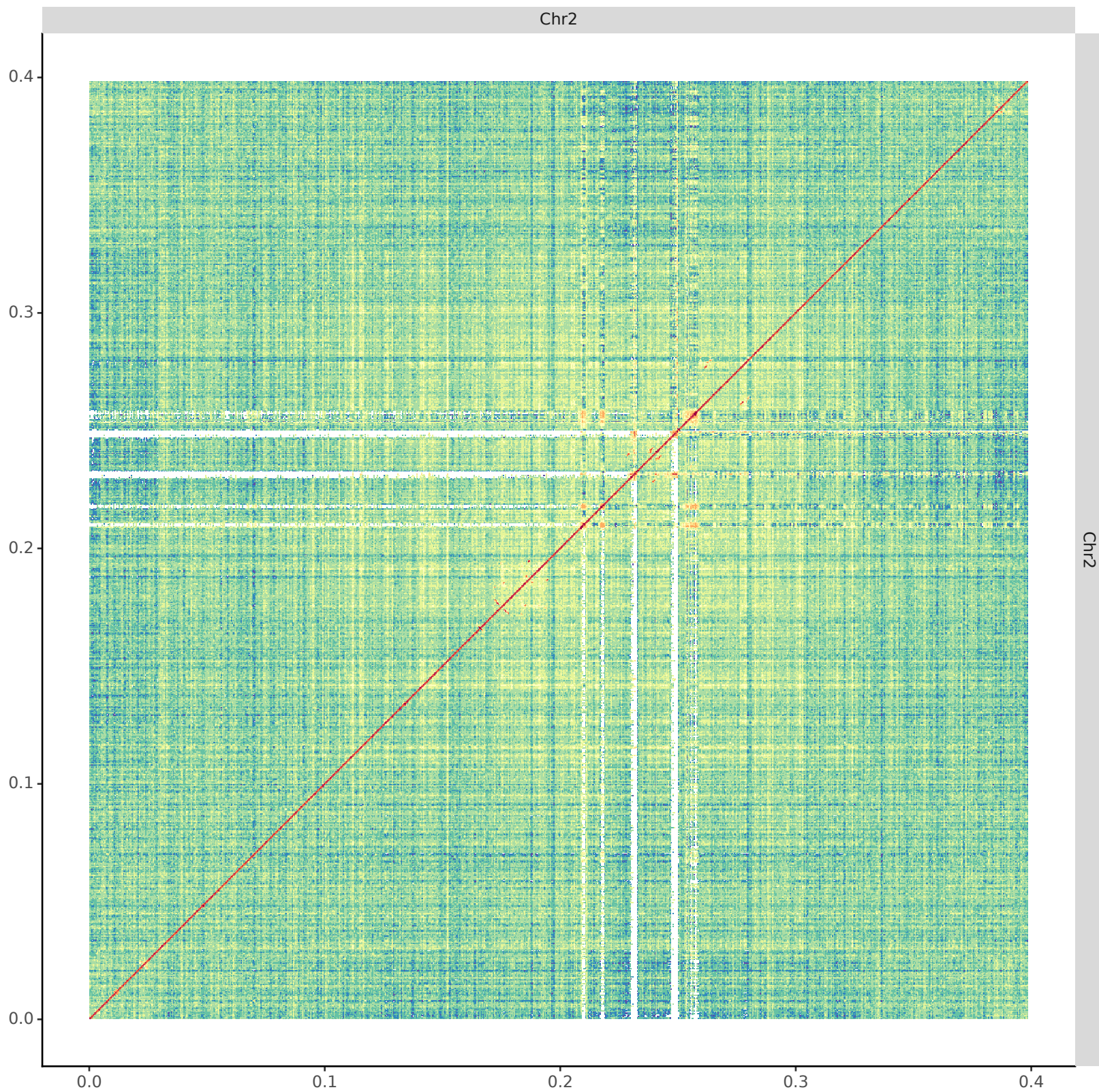

Supplement: Supplementary file 1 — Text S1. (a, b) Extraction of genomic DNA (gDNA) and (b) library preparation for Oxford Nanopore Technology (ONT) sequencing performed at the University of Göttingen. Text S2. Determining the optimal DNA sequence alignment for phylogenetic analyses. Text S3. Identification of tandem repeats (TRs) and transposable elements (TEs), and protein‐coding genes. Text S4. Detailed results of plastome‐based phylogenies in Ranunculaceae. Text S5. Impact of using frozen libraries for ONT DNA sequencing. Figure S1. Gel electrophoresis of gDNA extractions from 17th December 2021 using 1 kb DNA Ladder (New England Biolabs, Ipswich, MA, USA; 500 bp–10 kb) as size standard. Figure S2. (a–d) Maximum‐likelihood phylogeny based on min0 (no filtering), min50, min70, and min90 alignments of 306 plastomes (taxa) of the plant family Ranunculaceae. Figure S3. (a, b) Maximum‐likelihood phylogeny based on 306 plastomes (292 taxa) of the plant family Ranunculaceae. Figure S4. Maximum‐likelihood phylogeny based on 306 plastome sequences (292 taxa) and the min90 alignment of the plant family Ranunculaceae. Figure S5. Whole genome alignment analysis of (a) all available mitogenome sequences in Ranunculaceae, and (b) of the assembled Illumina‐ONT and ‐PacBio genome sequences of Ranunculus cassubicifolius (LH040). Figure S6. Concatenation‐based phylogeny of 10 mitogenome sequences and 42 genes of Ranunculaceae (see Figure 3b for the coalescent‐based phylogeny). Figure S7. Hi‐C contact map. Figure S8. (a–h) ModDotPlots of pseudochromosomes 1–8 of the final PacBio genome assembly (Table 1, ‘Nuclear Genome’). Figure S9. Detection of ancient whole genome duplication (WGD) events in Ranunculus cassubicifolius. Figure S10. BUSCO assessments (PacBio 25×) for different genome assembly strategies of the diploid sexual species Ranunculus cassubicifolius. Table S1. Selected (a) plastome and (b) mitogenome sequences from NCBI. Table S2. RNA‐seq data of 37 Ranunculaceae individuals from SRA/NCBI used for Ranunc [file TPJ-123-0-s001.zip › tpj70390-sup-0006-FigureS8/Figure_S8_ah_ModDotPlots_PDFs/Chr2_FULL.pdf]

./Chr3

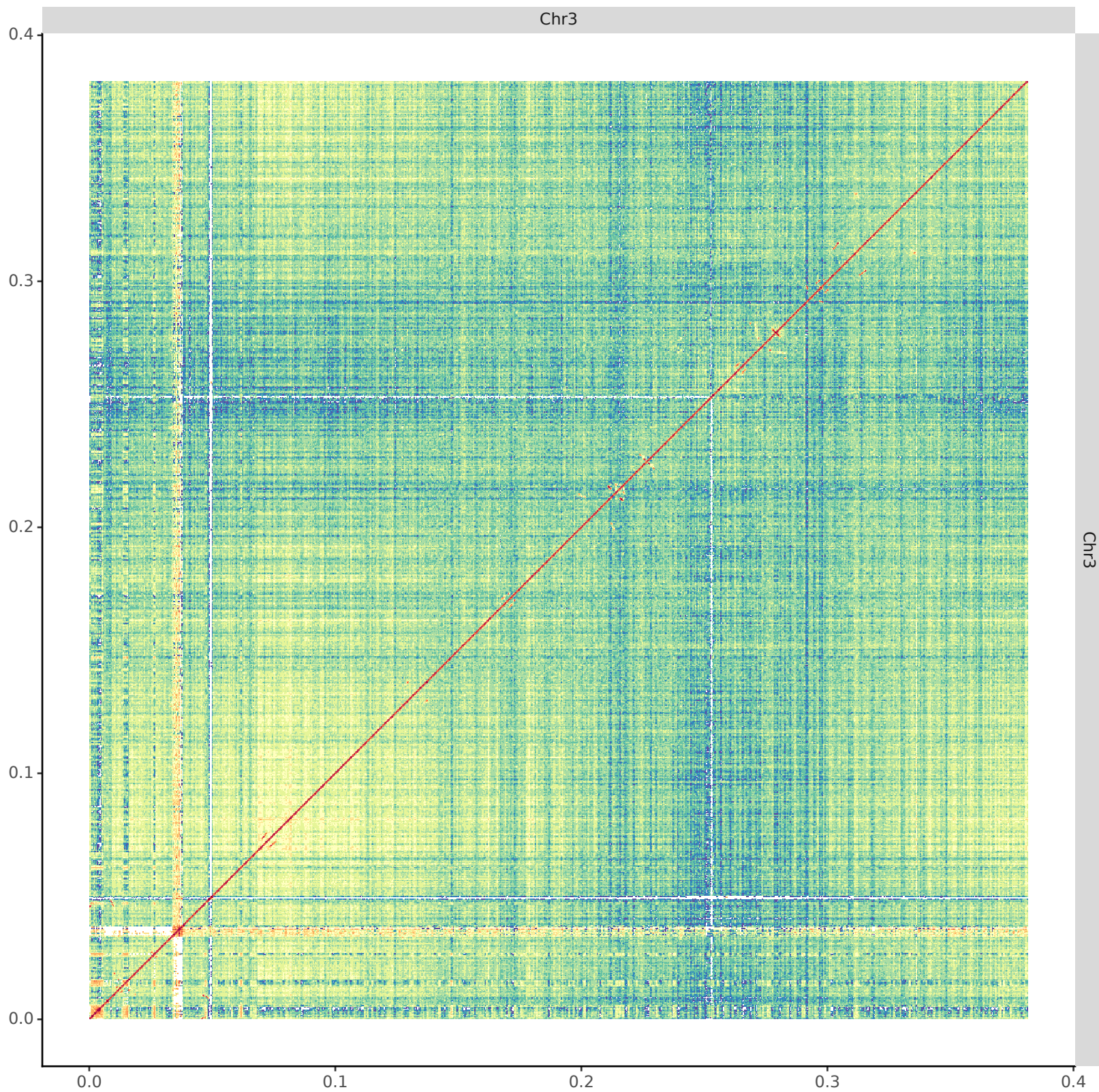

Supplement: Supplementary file 1 — Text S1. (a, b) Extraction of genomic DNA (gDNA) and (b) library preparation for Oxford Nanopore Technology (ONT) sequencing performed at the University of Göttingen. Text S2. Determining the optimal DNA sequence alignment for phylogenetic analyses. Text S3. Identification of tandem repeats (TRs) and transposable elements (TEs), and protein‐coding genes. Text S4. Detailed results of plastome‐based phylogenies in Ranunculaceae. Text S5. Impact of using frozen libraries for ONT DNA sequencing. Figure S1. Gel electrophoresis of gDNA extractions from 17th December 2021 using 1 kb DNA Ladder (New England Biolabs, Ipswich, MA, USA; 500 bp–10 kb) as size standard. Figure S2. (a–d) Maximum‐likelihood phylogeny based on min0 (no filtering), min50, min70, and min90 alignments of 306 plastomes (taxa) of the plant family Ranunculaceae. Figure S3. (a, b) Maximum‐likelihood phylogeny based on 306 plastomes (292 taxa) of the plant family Ranunculaceae. Figure S4. Maximum‐likelihood phylogeny based on 306 plastome sequences (292 taxa) and the min90 alignment of the plant family Ranunculaceae. Figure S5. Whole genome alignment analysis of (a) all available mitogenome sequences in Ranunculaceae, and (b) of the assembled Illumina‐ONT and ‐PacBio genome sequences of Ranunculus cassubicifolius (LH040). Figure S6. Concatenation‐based phylogeny of 10 mitogenome sequences and 42 genes of Ranunculaceae (see Figure 3b for the coalescent‐based phylogeny). Figure S7. Hi‐C contact map. Figure S8. (a–h) ModDotPlots of pseudochromosomes 1–8 of the final PacBio genome assembly (Table 1, ‘Nuclear Genome’). Figure S9. Detection of ancient whole genome duplication (WGD) events in Ranunculus cassubicifolius. Figure S10. BUSCO assessments (PacBio 25×) for different genome assembly strategies of the diploid sexual species Ranunculus cassubicifolius. Table S1. Selected (a) plastome and (b) mitogenome sequences from NCBI. Table S2. RNA‐seq data of 37 Ranunculaceae individuals from SRA/NCBI used for Ranunc [file TPJ-123-0-s001.zip › tpj70390-sup-0006-FigureS8/Figure_S8_ah_ModDotPlots_PDFs/Chr3_FULL.pdf]

./Chr4

Chr4

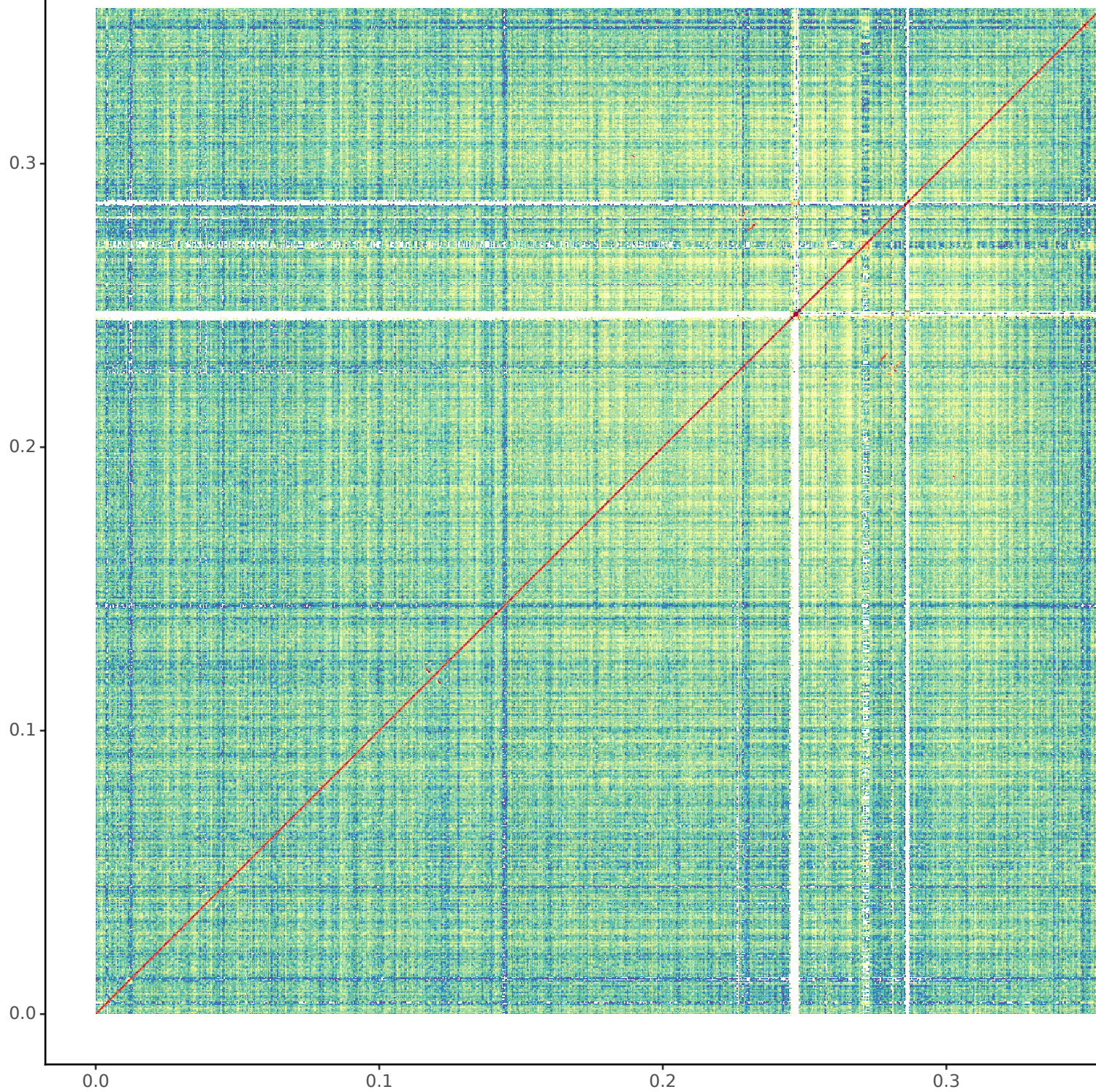

Chr4

Supplement: Supplementary file 1 — Text S1. (a, b) Extraction of genomic DNA (gDNA) and (b) library preparation for Oxford Nanopore Technology (ONT) sequencing performed at the University of Göttingen. Text S2. Determining the optimal DNA sequence alignment for phylogenetic analyses. Text S3. Identification of tandem repeats (TRs) and transposable elements (TEs), and protein‐coding genes. Text S4. Detailed results of plastome‐based phylogenies in Ranunculaceae. Text S5. Impact of using frozen libraries for ONT DNA sequencing. Figure S1. Gel electrophoresis of gDNA extractions from 17th December 2021 using 1 kb DNA Ladder (New England Biolabs, Ipswich, MA, USA; 500 bp–10 kb) as size standard. Figure S2. (a–d) Maximum‐likelihood phylogeny based on min0 (no filtering), min50, min70, and min90 alignments of 306 plastomes (taxa) of the plant family Ranunculaceae. Figure S3. (a, b) Maximum‐likelihood phylogeny based on 306 plastomes (292 taxa) of the plant family Ranunculaceae. Figure S4. Maximum‐likelihood phylogeny based on 306 plastome sequences (292 taxa) and the min90 alignment of the plant family Ranunculaceae. Figure S5. Whole genome alignment analysis of (a) all available mitogenome sequences in Ranunculaceae, and (b) of the assembled Illumina‐ONT and ‐PacBio genome sequences of Ranunculus cassubicifolius (LH040). Figure S6. Concatenation‐based phylogeny of 10 mitogenome sequences and 42 genes of Ranunculaceae (see Figure 3b for the coalescent‐based phylogeny). Figure S7. Hi‐C contact map. Figure S8. (a–h) ModDotPlots of pseudochromosomes 1–8 of the final PacBio genome assembly (Table 1, ‘Nuclear Genome’). Figure S9. Detection of ancient whole genome duplication (WGD) events in Ranunculus cassubicifolius. Figure S10. BUSCO assessments (PacBio 25×) for different genome assembly strategies of the diploid sexual species Ranunculus cassubicifolius. Table S1. Selected (a) plastome and (b) mitogenome sequences from NCBI. Table S2. RNA‐seq data of 37 Ranunculaceae individuals from SRA/NCBI used for Ranunc [file TPJ-123-0-s001.zip › tpj70390-sup-0006-FigureS8/Figure_S8_ah_ModDotPlots_PDFs/Chr4_FULL.pdf]

./Chr5

Chr5

0.3

0.2

0.1

0.0

0.0

0.1

0.2

0.3

Chr5

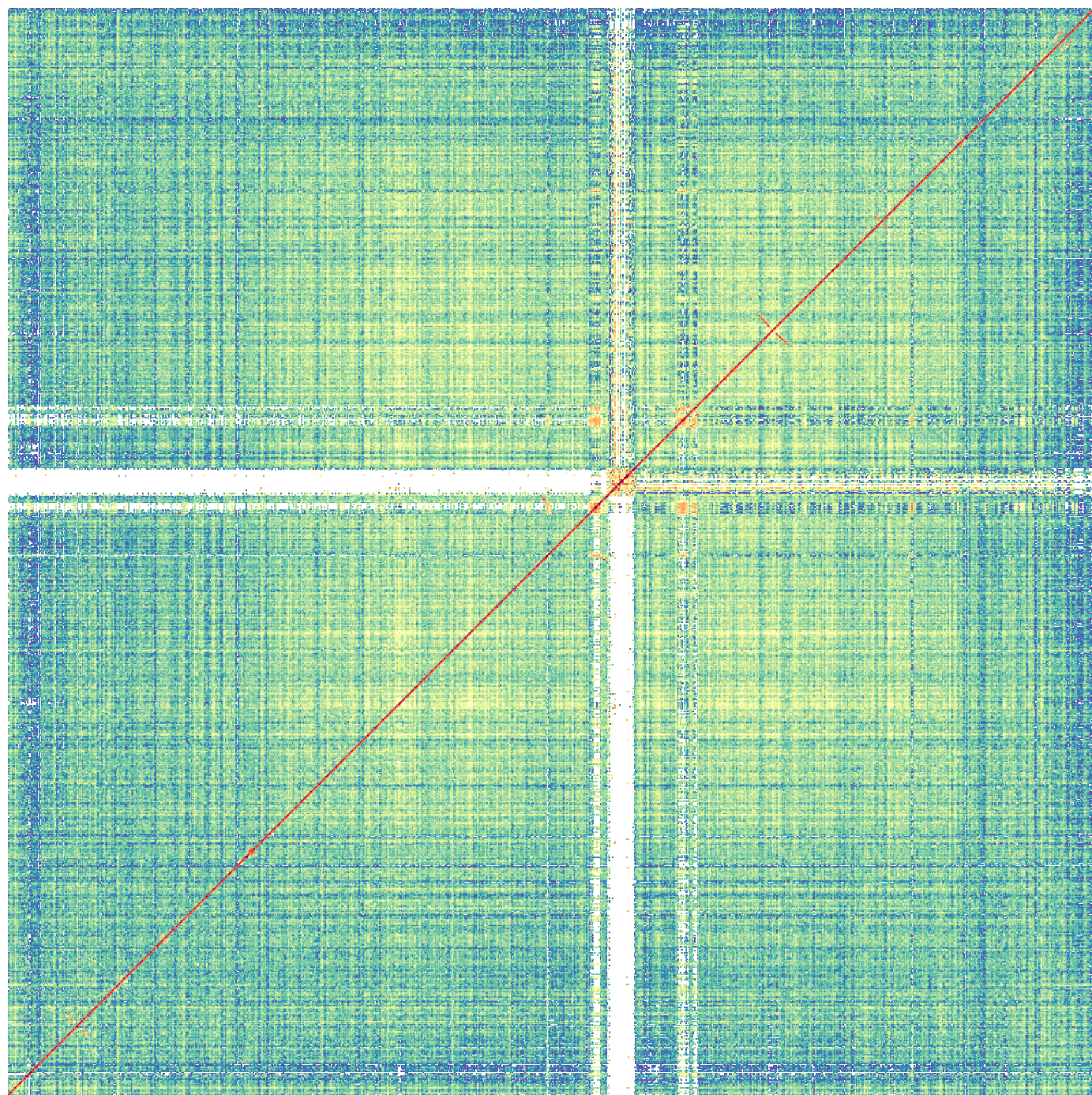

Supplement: Supplementary file 1 — Text S1. (a, b) Extraction of genomic DNA (gDNA) and (b) library preparation for Oxford Nanopore Technology (ONT) sequencing performed at the University of Göttingen. Text S2. Determining the optimal DNA sequence alignment for phylogenetic analyses. Text S3. Identification of tandem repeats (TRs) and transposable elements (TEs), and protein‐coding genes. Text S4. Detailed results of plastome‐based phylogenies in Ranunculaceae. Text S5. Impact of using frozen libraries for ONT DNA sequencing. Figure S1. Gel electrophoresis of gDNA extractions from 17th December 2021 using 1 kb DNA Ladder (New England Biolabs, Ipswich, MA, USA; 500 bp–10 kb) as size standard. Figure S2. (a–d) Maximum‐likelihood phylogeny based on min0 (no filtering), min50, min70, and min90 alignments of 306 plastomes (taxa) of the plant family Ranunculaceae. Figure S3. (a, b) Maximum‐likelihood phylogeny based on 306 plastomes (292 taxa) of the plant family Ranunculaceae. Figure S4. Maximum‐likelihood phylogeny based on 306 plastome sequences (292 taxa) and the min90 alignment of the plant family Ranunculaceae. Figure S5. Whole genome alignment analysis of (a) all available mitogenome sequences in Ranunculaceae, and (b) of the assembled Illumina‐ONT and ‐PacBio genome sequences of Ranunculus cassubicifolius (LH040). Figure S6. Concatenation‐based phylogeny of 10 mitogenome sequences and 42 genes of Ranunculaceae (see Figure 3b for the coalescent‐based phylogeny). Figure S7. Hi‐C contact map. Figure S8. (a–h) ModDotPlots of pseudochromosomes 1–8 of the final PacBio genome assembly (Table 1, ‘Nuclear Genome’). Figure S9. Detection of ancient whole genome duplication (WGD) events in Ranunculus cassubicifolius. Figure S10. BUSCO assessments (PacBio 25×) for different genome assembly strategies of the diploid sexual species Ranunculus cassubicifolius. Table S1. Selected (a) plastome and (b) mitogenome sequences from NCBI. Table S2. RNA‐seq data of 37 Ranunculaceae individuals from SRA/NCBI used for Ranunc [file TPJ-123-0-s001.zip › tpj70390-sup-0006-FigureS8/Figure_S8_ah_ModDotPlots_PDFs/Chr5_FULL.pdf]

./Chr6

Chr6

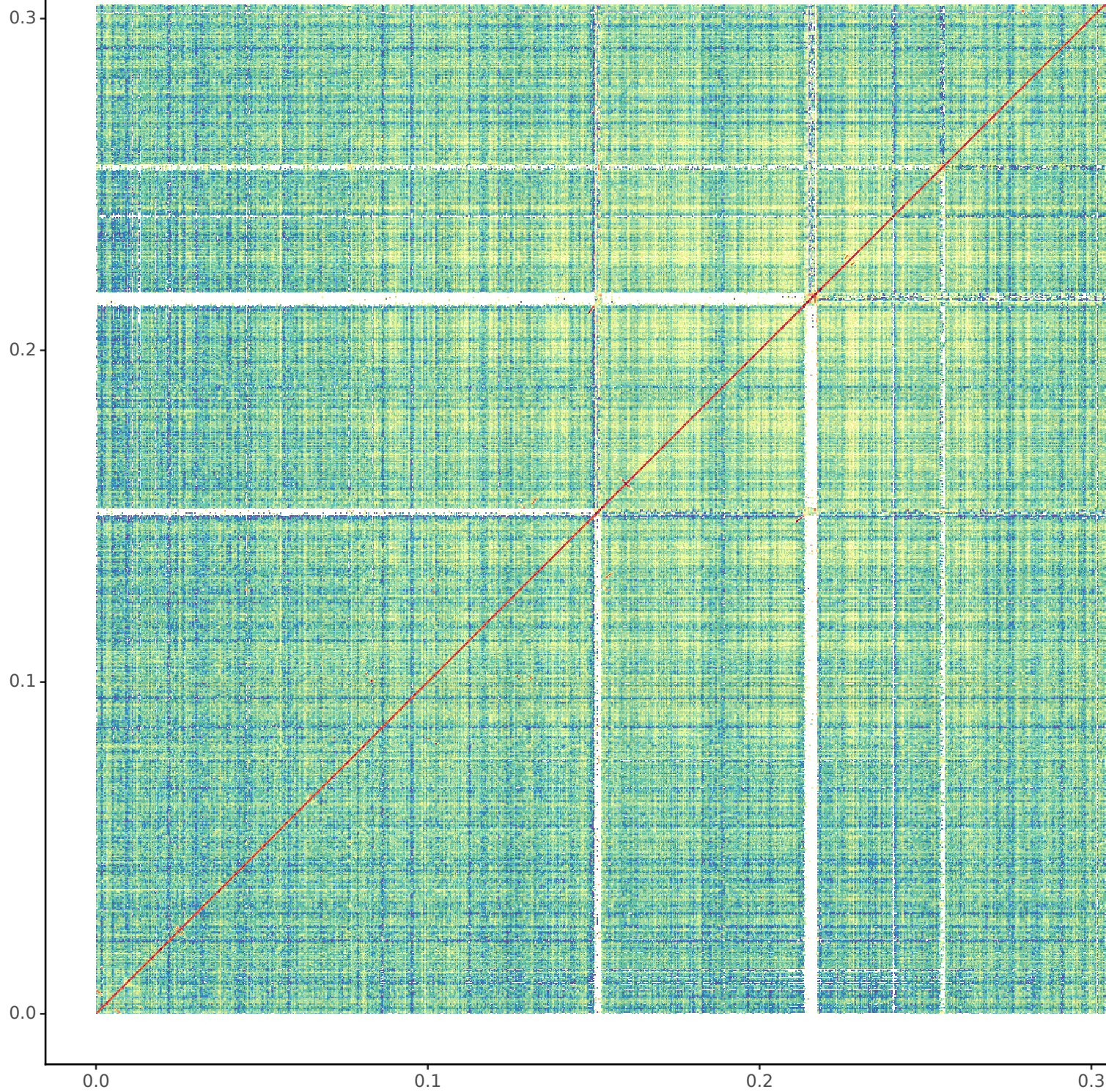

Supplement: Supplementary file 1 — Text S1. (a, b) Extraction of genomic DNA (gDNA) and (b) library preparation for Oxford Nanopore Technology (ONT) sequencing performed at the University of Göttingen. Text S2. Determining the optimal DNA sequence alignment for phylogenetic analyses. Text S3. Identification of tandem repeats (TRs) and transposable elements (TEs), and protein‐coding genes. Text S4. Detailed results of plastome‐based phylogenies in Ranunculaceae. Text S5. Impact of using frozen libraries for ONT DNA sequencing. Figure S1. Gel electrophoresis of gDNA extractions from 17th December 2021 using 1 kb DNA Ladder (New England Biolabs, Ipswich, MA, USA; 500 bp–10 kb) as size standard. Figure S2. (a–d) Maximum‐likelihood phylogeny based on min0 (no filtering), min50, min70, and min90 alignments of 306 plastomes (taxa) of the plant family Ranunculaceae. Figure S3. (a, b) Maximum‐likelihood phylogeny based on 306 plastomes (292 taxa) of the plant family Ranunculaceae. Figure S4. Maximum‐likelihood phylogeny based on 306 plastome sequences (292 taxa) and the min90 alignment of the plant family Ranunculaceae. Figure S5. Whole genome alignment analysis of (a) all available mitogenome sequences in Ranunculaceae, and (b) of the assembled Illumina‐ONT and ‐PacBio genome sequences of Ranunculus cassubicifolius (LH040). Figure S6. Concatenation‐based phylogeny of 10 mitogenome sequences and 42 genes of Ranunculaceae (see Figure 3b for the coalescent‐based phylogeny). Figure S7. Hi‐C contact map. Figure S8. (a–h) ModDotPlots of pseudochromosomes 1–8 of the final PacBio genome assembly (Table 1, ‘Nuclear Genome’). Figure S9. Detection of ancient whole genome duplication (WGD) events in Ranunculus cassubicifolius. Figure S10. BUSCO assessments (PacBio 25×) for different genome assembly strategies of the diploid sexual species Ranunculus cassubicifolius. Table S1. Selected (a) plastome and (b) mitogenome sequences from NCBI. Table S2. RNA‐seq data of 37 Ranunculaceae individuals from SRA/NCBI used for Ranunc [file TPJ-123-0-s001.zip › tpj70390-sup-0006-FigureS8/Figure_S8_ah_ModDotPlots_PDFs/Chr6_FULL.pdf]

./Chr7

Chr7

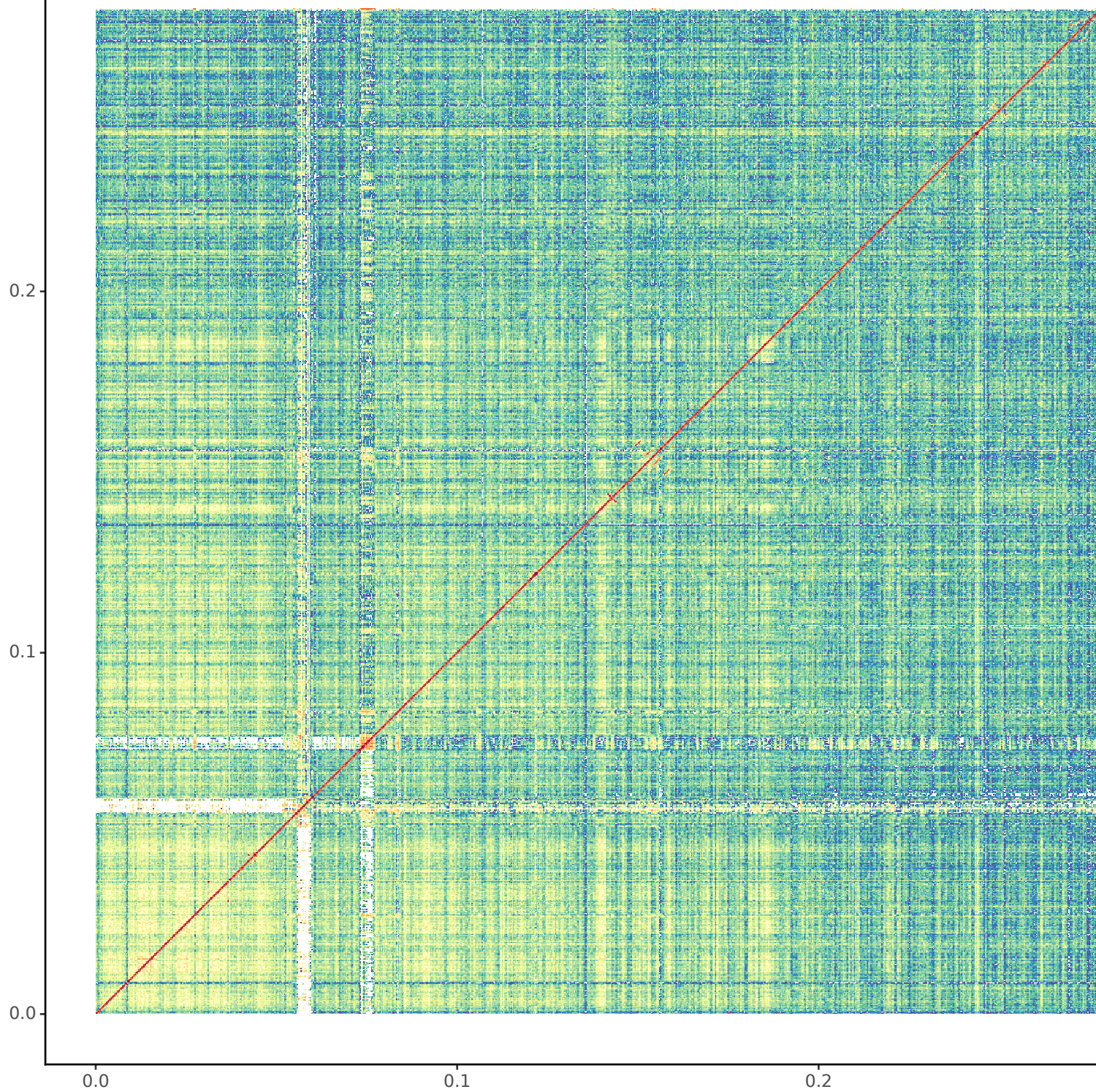

Chr7

Supplement: Supplementary file 1 — Text S1. (a, b) Extraction of genomic DNA (gDNA) and (b) library preparation for Oxford Nanopore Technology (ONT) sequencing performed at the University of Göttingen. Text S2. Determining the optimal DNA sequence alignment for phylogenetic analyses. Text S3. Identification of tandem repeats (TRs) and transposable elements (TEs), and protein‐coding genes. Text S4. Detailed results of plastome‐based phylogenies in Ranunculaceae. Text S5. Impact of using frozen libraries for ONT DNA sequencing. Figure S1. Gel electrophoresis of gDNA extractions from 17th December 2021 using 1 kb DNA Ladder (New England Biolabs, Ipswich, MA, USA; 500 bp–10 kb) as size standard. Figure S2. (a–d) Maximum‐likelihood phylogeny based on min0 (no filtering), min50, min70, and min90 alignments of 306 plastomes (taxa) of the plant family Ranunculaceae. Figure S3. (a, b) Maximum‐likelihood phylogeny based on 306 plastomes (292 taxa) of the plant family Ranunculaceae. Figure S4. Maximum‐likelihood phylogeny based on 306 plastome sequences (292 taxa) and the min90 alignment of the plant family Ranunculaceae. Figure S5. Whole genome alignment analysis of (a) all available mitogenome sequences in Ranunculaceae, and (b) of the assembled Illumina‐ONT and ‐PacBio genome sequences of Ranunculus cassubicifolius (LH040). Figure S6. Concatenation‐based phylogeny of 10 mitogenome sequences and 42 genes of Ranunculaceae (see Figure 3b for the coalescent‐based phylogeny). Figure S7. Hi‐C contact map. Figure S8. (a–h) ModDotPlots of pseudochromosomes 1–8 of the final PacBio genome assembly (Table 1, ‘Nuclear Genome’). Figure S9. Detection of ancient whole genome duplication (WGD) events in Ranunculus cassubicifolius. Figure S10. BUSCO assessments (PacBio 25×) for different genome assembly strategies of the diploid sexual species Ranunculus cassubicifolius. Table S1. Selected (a) plastome and (b) mitogenome sequences from NCBI. Table S2. RNA‐seq data of 37 Ranunculaceae individuals from SRA/NCBI used for Ranunc [file TPJ-123-0-s001.zip › tpj70390-sup-0006-FigureS8/Figure_S8_ah_ModDotPlots_PDFs/Chr7_FULL.pdf]

./Chr8

Chr8

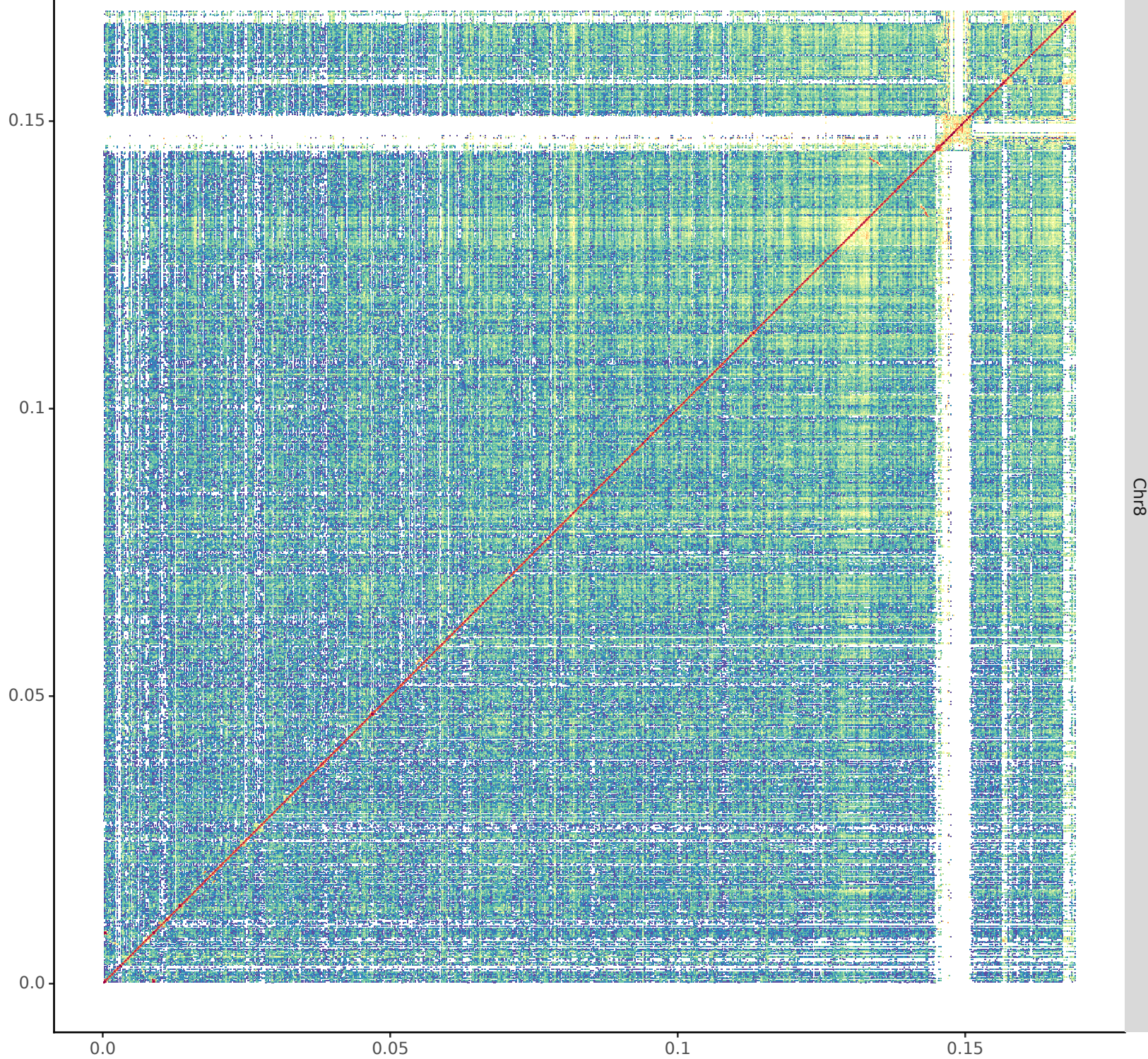

Chr8

Supplement: Supplementary file 1 — Text S1. (a, b) Extraction of genomic DNA (gDNA) and (b) library preparation for Oxford Nanopore Technology (ONT) sequencing performed at the University of Göttingen. Text S2. Determining the optimal DNA sequence alignment for phylogenetic analyses. Text S3. Identification of tandem repeats (TRs) and transposable elements (TEs), and protein‐coding genes. Text S4. Detailed results of plastome‐based phylogenies in Ranunculaceae. Text S5. Impact of using frozen libraries for ONT DNA sequencing. Figure S1. Gel electrophoresis of gDNA extractions from 17th December 2021 using 1 kb DNA Ladder (New England Biolabs, Ipswich, MA, USA; 500 bp–10 kb) as size standard. Figure S2. (a–d) Maximum‐likelihood phylogeny based on min0 (no filtering), min50, min70, and min90 alignments of 306 plastomes (taxa) of the plant family Ranunculaceae. Figure S3. (a, b) Maximum‐likelihood phylogeny based on 306 plastomes (292 taxa) of the plant family Ranunculaceae. Figure S4. Maximum‐likelihood phylogeny based on 306 plastome sequences (292 taxa) and the min90 alignment of the plant family Ranunculaceae. Figure S5. Whole genome alignment analysis of (a) all available mitogenome sequences in Ranunculaceae, and (b) of the assembled Illumina‐ONT and ‐PacBio genome sequences of Ranunculus cassubicifolius (LH040). Figure S6. Concatenation‐based phylogeny of 10 mitogenome sequences and 42 genes of Ranunculaceae (see Figure 3b for the coalescent‐based phylogeny). Figure S7. Hi‐C contact map. Figure S8. (a–h) ModDotPlots of pseudochromosomes 1–8 of the final PacBio genome assembly (Table 1, ‘Nuclear Genome’). Figure S9. Detection of ancient whole genome duplication (WGD) events in Ranunculus cassubicifolius. Figure S10. BUSCO assessments (PacBio 25×) for different genome assembly strategies of the diploid sexual species Ranunculus cassubicifolius. Table S1. Selected (a) plastome and (b) mitogenome sequences from NCBI. Table S2. RNA‐seq data of 37 Ranunculaceae individuals from SRA/NCBI used for Ranunc [file TPJ-123-0-s001.zip › tpj70390-sup-0006-FigureS8/Figure_S8_ah_ModDotPlots_PDFs/Chr8_FULL.pdf]

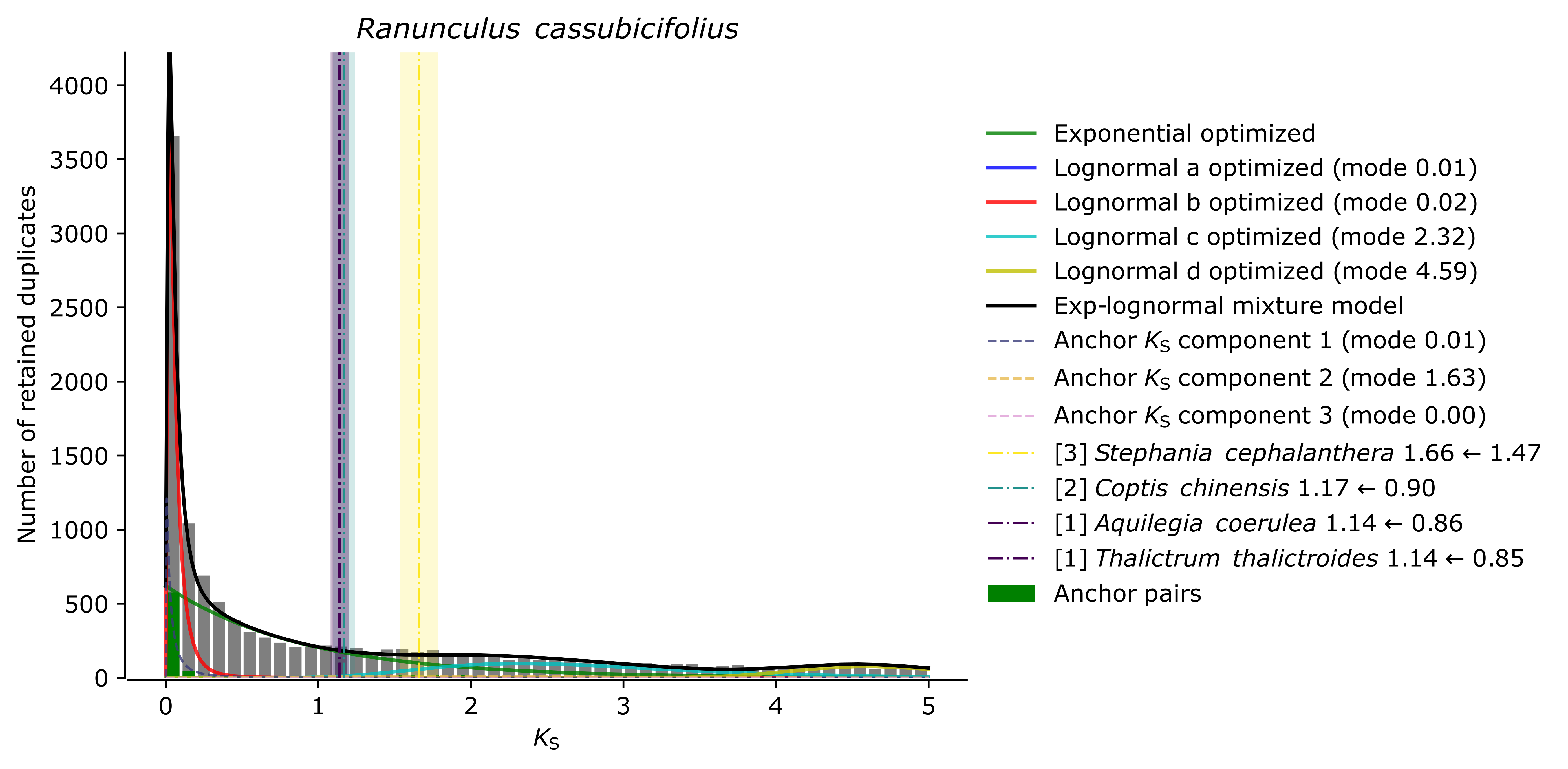

Supplement: Supplementary file 1 — Text S1. (a, b) Extraction of genomic DNA (gDNA) and (b) library preparation for Oxford Nanopore Technology (ONT) sequencing performed at the University of Göttingen. Text S2. Determining the optimal DNA sequence alignment for phylogenetic analyses. Text S3. Identification of tandem repeats (TRs) and transposable elements (TEs), and protein‐coding genes. Text S4. Detailed results of plastome‐based phylogenies in Ranunculaceae. Text S5. Impact of using frozen libraries for ONT DNA sequencing. Figure S1. Gel electrophoresis of gDNA extractions from 17th December 2021 using 1 kb DNA Ladder (New England Biolabs, Ipswich, MA, USA; 500 bp–10 kb) as size standard. Figure S2. (a–d) Maximum‐likelihood phylogeny based on min0 (no filtering), min50, min70, and min90 alignments of 306 plastomes (taxa) of the plant family Ranunculaceae. Figure S3. (a, b) Maximum‐likelihood phylogeny based on 306 plastomes (292 taxa) of the plant family Ranunculaceae. Figure S4. Maximum‐likelihood phylogeny based on 306 plastome sequences (292 taxa) and the min90 alignment of the plant family Ranunculaceae. Figure S5. Whole genome alignment analysis of (a) all available mitogenome sequences in Ranunculaceae, and (b) of the assembled Illumina‐ONT and ‐PacBio genome sequences of Ranunculus cassubicifolius (LH040). Figure S6. Concatenation‐based phylogeny of 10 mitogenome sequences and 42 genes of Ranunculaceae (see Figure 3b for the coalescent‐based phylogeny). Figure S7. Hi‐C contact map. Figure S8. (a–h) ModDotPlots of pseudochromosomes 1–8 of the final PacBio genome assembly (Table 1, ‘Nuclear Genome’). Figure S9. Detection of ancient whole genome duplication (WGD) events in Ranunculus cassubicifolius. Figure S10. BUSCO assessments (PacBio 25×) for different genome assembly strategies of the diploid sexual species Ranunculus cassubicifolius. Table S1. Selected (a) plastome and (b) mitogenome sequences from NCBI. Table S2. RNA‐seq data of 37 Ranunculaceae individuals from SRA/NCBI used for Ranunc [file TPJ-123-0-s001.zip › tpj70390-sup-0007-FigureS9/Figure_S9a_interspecies_KS_Plot_WGD.png]

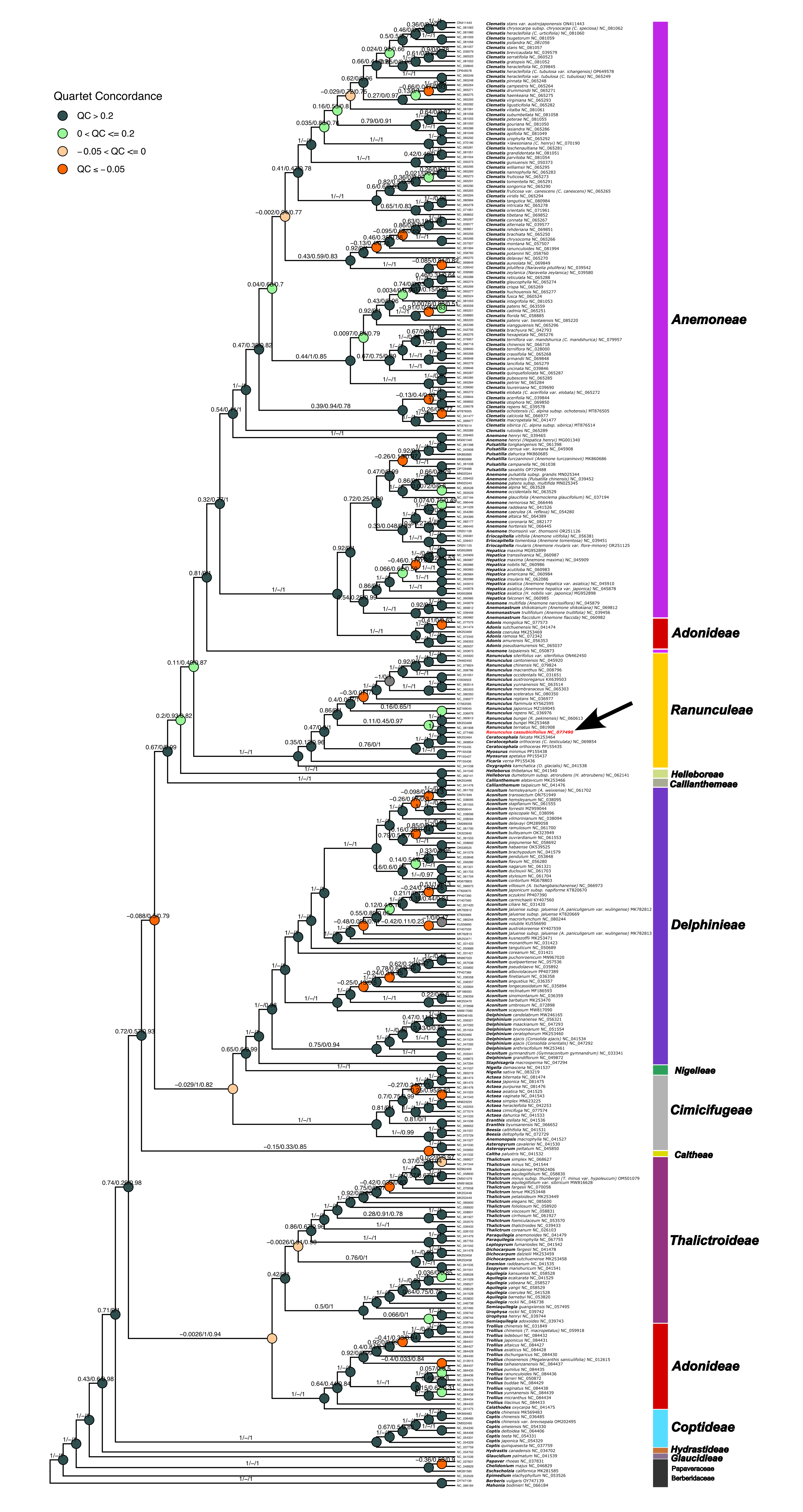

Supplement: Supplementary file 1 — Text S1. (a, b) Extraction of genomic DNA (gDNA) and (b) library preparation for Oxford Nanopore Technology (ONT) sequencing performed at the University of Göttingen. Text S2. Determining the optimal DNA sequence alignment for phylogenetic analyses. Text S3. Identification of tandem repeats (TRs) and transposable elements (TEs), and protein‐coding genes. Text S4. Detailed results of plastome‐based phylogenies in Ranunculaceae. Text S5. Impact of using frozen libraries for ONT DNA sequencing. Figure S1. Gel electrophoresis of gDNA extractions from 17th December 2021 using 1 kb DNA Ladder (New England Biolabs, Ipswich, MA, USA; 500 bp–10 kb) as size standard. Figure S2. (a–d) Maximum‐likelihood phylogeny based on min0 (no filtering), min50, min70, and min90 alignments of 306 plastomes (taxa) of the plant family Ranunculaceae. Figure S3. (a, b) Maximum‐likelihood phylogeny based on 306 plastomes (292 taxa) of the plant family Ranunculaceae. Figure S4. Maximum‐likelihood phylogeny based on 306 plastome sequences (292 taxa) and the min90 alignment of the plant family Ranunculaceae. Figure S5. Whole genome alignment analysis of (a) all available mitogenome sequences in Ranunculaceae, and (b) of the assembled Illumina‐ONT and ‐PacBio genome sequences of Ranunculus cassubicifolius (LH040). Figure S6. Concatenation‐based phylogeny of 10 mitogenome sequences and 42 genes of Ranunculaceae (see Figure 3b for the coalescent‐based phylogeny). Figure S7. Hi‐C contact map. Figure S8. (a–h) ModDotPlots of pseudochromosomes 1–8 of the final PacBio genome assembly (Table 1, ‘Nuclear Genome’). Figure S9. Detection of ancient whole genome duplication (WGD) events in Ranunculus cassubicifolius. Figure S10. BUSCO assessments (PacBio 25×) for different genome assembly strategies of the diploid sexual species Ranunculus cassubicifolius. Table S1. Selected (a) plastome and (b) mitogenome sequences from NCBI. Table S2. RNA‐seq data of 37 Ranunculaceae individuals from SRA/NCBI used for Ranunc [file TPJ-123-0-s001.zip › tpj70390-sup-0004-FigureS4.png]

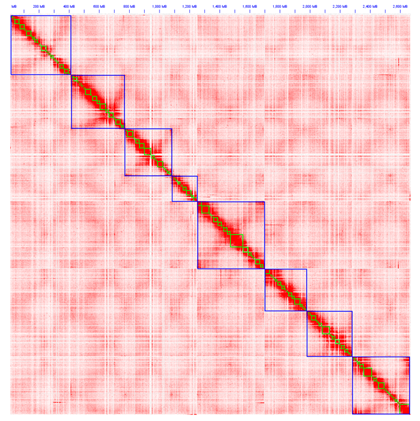

Supplement: Supplementary file 1 — Text S1. (a, b) Extraction of genomic DNA (gDNA) and (b) library preparation for Oxford Nanopore Technology (ONT) sequencing performed at the University of Göttingen. Text S2. Determining the optimal DNA sequence alignment for phylogenetic analyses. Text S3. Identification of tandem repeats (TRs) and transposable elements (TEs), and protein‐coding genes. Text S4. Detailed results of plastome‐based phylogenies in Ranunculaceae. Text S5. Impact of using frozen libraries for ONT DNA sequencing. Figure S1. Gel electrophoresis of gDNA extractions from 17th December 2021 using 1 kb DNA Ladder (New England Biolabs, Ipswich, MA, USA; 500 bp–10 kb) as size standard. Figure S2. (a–d) Maximum‐likelihood phylogeny based on min0 (no filtering), min50, min70, and min90 alignments of 306 plastomes (taxa) of the plant family Ranunculaceae. Figure S3. (a, b) Maximum‐likelihood phylogeny based on 306 plastomes (292 taxa) of the plant family Ranunculaceae. Figure S4. Maximum‐likelihood phylogeny based on 306 plastome sequences (292 taxa) and the min90 alignment of the plant family Ranunculaceae. Figure S5. Whole genome alignment analysis of (a) all available mitogenome sequences in Ranunculaceae, and (b) of the assembled Illumina‐ONT and ‐PacBio genome sequences of Ranunculus cassubicifolius (LH040). Figure S6. Concatenation‐based phylogeny of 10 mitogenome sequences and 42 genes of Ranunculaceae (see Figure 3b for the coalescent‐based phylogeny). Figure S7. Hi‐C contact map. Figure S8. (a–h) ModDotPlots of pseudochromosomes 1–8 of the final PacBio genome assembly (Table 1, ‘Nuclear Genome’). Figure S9. Detection of ancient whole genome duplication (WGD) events in Ranunculus cassubicifolius. Figure S10. BUSCO assessments (PacBio 25×) for different genome assembly strategies of the diploid sexual species Ranunculus cassubicifolius. Table S1. Selected (a) plastome and (b) mitogenome sequences from NCBI. Table S2. RNA‐seq data of 37 Ranunculaceae individuals from SRA/NCBI used for Ranunc [file TPJ-123-0-s001.zip › tpj70390-sup-0005-FigureS7.png]
